# Strategies for Understanding and Reducing the *Plasmodium vivax* and *Plasmodium ovale* Hypnozoite Reservoir in Papua New Guinean Children: A Randomised Placebo-Controlled Trial and Mathematical Model

**DOI:** 10.1371/journal.pmed.1001891

**Published:** 2015-10-27

**Authors:** Leanne J. Robinson, Rahel Wampfler, Inoni Betuela, Stephan Karl, Michael T. White, Connie S. N. Li Wai Suen, Natalie E. Hofmann, Benson Kinboro, Andreea Waltmann, Jessica Brewster, Lina Lorry, Nandao Tarongka, Lornah Samol, Mariabeth Silkey, Quique Bassat, Peter M. Siba, Louis Schofield, Ingrid Felger, Ivo Mueller

**Affiliations:** 1 Vector Borne Diseases Unit, Papua New Guinea Institute of Medical Research, Madang and Maprik, Papua New Guinea; 2 Population Health and Immunity Division, Walter and Eliza Hall Institute of Medical Research, Parkville, Victoria, Australia; 3 Department of Medical Biology, University of Melbourne, Melbourne, Victoria, Australia; 4 Molecular Diagnostics Unit, Swiss Tropical and Public Health Institute, Basel, Switzerland; 5 University of Basel, Basel, Switzerland; 6 MRC Centre for Outbreak Analysis and Modelling, Imperial College London, London, United Kingdom; 7 Australian Institute of Tropical Health and Medicine, James Cook University, Cairns, Queensland, Australia; 8 School of Veterinary and Biomedical Sciences, James Cook University, Townsville, Queensland, Australia; 9 ISGlobal, Barcelona Centre for International Health Research (CRESIB), Hospital Clínic–University of Barcelona, Barcelona, Spain; Mahidol-Oxford Tropical Medicine Research Unit, THAILAND

## Abstract

**Background:**

The undetectable hypnozoite reservoir for relapsing *Plasmodium vivax* and *P*. *ovale* malarias presents a major challenge for malaria control and elimination in endemic countries. This study aims to directly determine the contribution of relapses to the burden of *P*. *vivax* and *P*. *ovale* infection, illness, and transmission in Papua New Guinean children.

**Methods and Findings:**

From 17 August 2009 to 20 May 2010, 524 children aged 5–10 y from East Sepik Province in Papua New Guinea (PNG) participated in a randomised double-blind placebo-controlled trial of blood- plus liver-stage drugs (chloroquine [CQ], 3 d; artemether-lumefantrine [AL], 3 d; and primaquine [PQ], 20 d, 10 mg/kg total dose) (261 children) or blood-stage drugs only (CQ, 3 d; AL, 3 d; and placebo [PL], 20 d) (263 children). Participants, study staff, and investigators were blinded to the treatment allocation. Twenty children were excluded during the treatment phase (PQ arm: 14, PL arm: 6), and 504 were followed actively for 9 mo. During the follow-up time, 18 children (PQ arm: 7, PL arm: 11) were lost to follow-up. Main primary and secondary outcome measures were time to first *P*. *vivax* infection (by qPCR), time to first clinical episode, force of infection, gametocyte positivity, and time to first *P*. *ovale* infection (by PCR). A basic stochastic transmission model was developed to estimate the potential effect of mass drug administration (MDA) for the prevention of recurrent *P*. *vivax* infections. Targeting hypnozoites through PQ treatment reduced the risk of having at least one qPCR-detectable *P*. *vivax* or *P*. *ovale* infection during 8 mo of follow-up (*P*. *vivax*: PQ arm 0.63/y versus PL arm 2.62/y, HR = 0.18 [95% CI 0.14, 0.25], *p <* 0.001; *P*. *ovale*: 0.06 versus 0.14, HR = 0.31 [95% CI 0.13, 0.77], *p* = 0.011) and the risk of having at least one clinical *P*. *vivax* episode (HR = 0.25 [95% CI 0.11, 0.61], *p* = 0.002). PQ also reduced the molecular force of *P*. *vivax* blood-stage infection in the first 3 mo of follow-up (PQ arm 1.90/y versus PL arm 7.75/y, incidence rate ratio [IRR] = 0.21 [95% CI 0.15, 0.28], *p <* 0.001). Children who received PQ were less likely to carry *P*. *vivax* gametocytes (IRR = 0.27 [95% CI 0.19, 0.38], *p <* 0.001). PQ had a comparable effect irrespective of the presence of *P*. *vivax* blood-stage infection at the time of treatment (*p* = 0.14). Modelling revealed that mass screening and treatment with highly sensitive quantitative real-time PCR, or MDA with blood-stage treatment alone, would have only a transient effect on *P*. *vivax* transmission levels, while MDA that includes liver-stage treatment is predicted to be a highly effective strategy for *P*. *vivax* elimination. The inclusion of a directly observed 20-d treatment regime maximises the efficiency of hypnozoite clearance but limits the generalisability of results to real-world MDA programmes.

**Conclusions:**

These results suggest that relapses cause approximately four of every five *P*. *vivax* infections and at least three of every five *P*. *ovale* infections in PNG children and are important in sustaining transmission. MDA campaigns combining blood- and liver-stage treatment are predicted to be a highly efficacious intervention for reducing *P*. *vivax* and *P*. *ovale* transmission.

**Trial registration:**

ClinicalTrials.gov NCT02143934

## Abstract 2

Ivo Mueller and colleagues conduct a randomized controlled trial of blood-only or blood- plus liver-stage malaria drugs to identify the contribution of relapses to the burden of *P*. *vivax* and *P*. *ovale* in children in Papua New Guinea, and model the potential impact of mass-drug administration to prevent recurrent *P*. *vivax* infections.

## Editors' Summary

**Background:**

Malaria is a mosquito-borne parasitic disease caused by *Plasmodium falciparum*, *P*. *vivax*, *P*. *ovale*, and *P*. *malariae*. Although *P*. *falciparum* is responsible for most of the 600,000 malaria deaths that occur every year, *P*. *vivax* is the most common, most widely distributed cause of malaria. All malaria parasites have a complex life cycle. When infected mosquitoes bite people, they inject “sporozoites,” a parasitic form that replicates in the liver. After 8–9 days, the liver releases “merozoites,” which invade red blood cells, where they replicate rapidly before bursting out and infecting more red blood cells. This increase in the parasitic burden causes malaria’s recurring flu-like symptoms and can cause organ damage and death. Infected red blood cells also release “gametocytes,” which infect mosquitoes when they take a blood meal. In the mosquito, gametocytes multiply and develop into sporozoites, thus completing the parasite’s life cycle. Malaria can be prevented by controlling the mosquitoes that spread malaria and by avoiding mosquito bites. Treatment with anti-malarial drugs, which is essential to prevent potentially fatal complications, also decreases malaria transmission.

**Why Was This Study Done?:**

Malaria control programs have greatly reduced the global malaria burden, but the ability of *P*. *vivax* and *P*. *ovale* to persist undetected in the liver as “hypnozoites” (another parasitic form) is hindering malaria control and elimination efforts. Hypnozoites can cause malaria relapses months or years after a primary infection and are not cleared by anti-malarial treatment unless the treatment includes primaquine, a drug that has to be given for 7–14 days and that causes hemolysis (red blood cell death) in people who have glucose-6-phosphate dehydrogenase (G6PD) deficiency. Here, the researchers determine the contribution that relapses make to the burden of *P*. *vivax* and *P*. *ovale* infection, illness, and transmission among Papua New Guinean children by undertaking a randomized placebo-controlled trial of a primaquine treatment regimen. The researchers also use mathematical modeling to investigate the effect of mass drug administration (MDA) on the occurrence of *P*. *vivax* relapses. A randomized placebo-controlled trial compares the outcomes of individuals randomly chosen to receive an active treatment or a dummy (placebo) treatment; MDA aims to control malaria by treating entire at-risk populations with anti-malarial drugs.

**What Did the Researchers Do and Find?:**

The researchers enrolled 524 children aged 5–10 years (none of whom were G6PD deficient) living in a region of Papua New Guinea where *P*. *falciparum* and *P*. *vivax* are hyperendemic (always present at high levels). Half the children received a blood-stage plus liver-stage anti-malarial treatment regimen (primaquine arm); the rest received a blood-stage plus placebo anti-malarial treatment regimen (placebo arm). Compared to children in the placebo arm, children in the primaquine arm had a reduced risk of having at least one *P*. *vivax* or *P*. *ovale* infection detected using PCR (a highly sensitive molecular technique) during eight months of follow-up and a reduced risk of having at least one clinical *P*. *vivax* episode. Children in the primaquine arm were also less likely to carry *P*. *vivax* gametocytes. Finally, by feeding the trial data into a mathematical transmission model, the researchers predicted that MDA with blood-stage treatment alone, or mass screening and treatment with blood-stage treatment alone or blood- plus liver-stage treatment, would have only a transient effect on *P*. *vivax* transmission levels, whereas MDA that includes blood-plus liver-stage treatment would be an effective strategy for *P*. *vivax* elimination.

**What Do These Findings Mean?:**

From the trial results, the researchers estimate that, among children living in a malaria-hyperendemic region of Papua New Guinea, relapses cause about four of every five *P*. *vivax* infections and three of every five *P*. *ovale* infections. Thus, *P*. *vivax* and *P*. *ovale* relapses are important in sustaining malaria transmission in this population. Notably, mathematical modeling predicts that MDA campaigns that combine blood- and liver-stage treatment are likely to be a highly effective strategy for *P*. *vivax* elimination. These findings may not be generalizable to populations with lower malaria transmission levels. Also, because the trial used a 20-day drug regimen to maximize the clearance of hypnozoites and because people would need to be tested for G6PD deficiency before starting primaquine treatment, the chosen treatment regimen may not be applicable to real-world MDA programs. Nevertheless, these findings highlight the importance of developing new anti-hypnozoite drugs and MDA programs that target areas and risk groups with confirmed local transmission of *P*. *vivax* to achieve global malaria control and elimination.

**Additional Information:**

This list of resources contains links that can be accessed when viewing the PDF on a device or via the online version of the article at http://dx.doi.org/10.1371/journal.pmed.1001891.

- Information is available from the World Health Organization on malaria (in several languages); the World Malaria Report 2014 provides details of the current global malaria situation, including information on malaria in Papua New Guinea; the 2015 guidelines for the treatment of malaria are available
- The US Centers for Disease Control and Prevention provides information on malaria (in English and Spanish), including information on different malaria parasites, mass drug administration, and personal stories about malaria
- Information is available from the Roll Back Malaria Partnership on the global control of malaria
- The Malaria Vaccine Initiative has a fact sheet on P. vivax malaria
- The Scientists Against Malaria collaboration applies modern drug design and modeling techniques to developing new treatments against malaria; its website includes information about many aspects of malaria
- MedlinePlus provides links to additional information on malaria (in English and Spanish)
- Wikipedia has a page on mass drug administration (note that Wikipedia is a free online encyclopedia that anyone can edit; available in several languages)
- More information about this trial is available

## Introduction

Renewed intensification of global malaria control efforts over the last 15 years have been successful in significantly reducing the global burden of malaria, with many countries in the Asia-Pacific and the Americas seeing a reduction of >90% in the number of clinical cases [1]. As a consequence, 34 countries are actively attempting to eliminate malaria, and many others are considering doing so in the near future [2]. In 2013, political leaders in Central American and East Asian countries, representing >60% of the global population, declared their intention to eliminate malaria in their regions by 2020 and 2030, respectively [3,4]. In parallel to the reduction in overall incidence, a pronounced shift in species composition has been observed, with *Plasmodium vivax* now the predominant *Plasmodium* species in the vast majority of countries outside Africa [2], accounting for 90%–100% of clinical cases in countries such as Guatemala, Brazil, Solomon Islands, and Vanuatu [1].

Despite a significant reduction in *P*. *vivax* malaria in the last 20 years, *P*. *vivax* has several biological characteristics that enable it to evade existing control and elimination efforts, which are mainly directed against *P*. *falciparum* blood stages [5,6]. First and foremost is the ability of *P*. *vivax* to relapse weeks, months, and years after a primary infection, via a poorly understood reactivation of dormant hypnozoite stages in the liver [7]. These stages cannot be detected with currently available diagnostic tools and are not cleared upon treatment with routinely administered anti-malarial drugs, unless primaquine (PQ)—a drug that requires at least 7–14 d of administration and can cause severe haemolysis in people with glucose-6-phosphate dehydrogenase (G6PD) deficiency—is added to the treatment [8].

In *P*. *vivax*–endemic regions, hypnozoites constitute a reservoir of diverse *P*. *vivax* strains that will cause blood-stage infections at a later point in time [9,10]. They are therefore likely not only to account for a high number of *P*. *vivax* blood-stage infections but also to contribute a high number of concurrently circulating parasite clones in the blood. Despite recent advances in the molecular detection and genotyping of *P*. *vivax* parasites [11], it is not yet possible to determine whether an infection detected in the blood of an individual originated from a new sporozoite inoculation or is a relapse from a hypnozoite. *P*. *vivax* produces gametocytes rapidly and continuously over the course of an infection [10], and even low-density infections are thus potentially infectious. If all clones, relapse-derived and newly acquired, produce gametocytes concurrently, these infections can potentially be transmitted together, and the chance for sexual recombination in the mosquito is greatly increased, thus contributing to the maintenance of high *P*. *vivax* genetic diversity even at low transmission levels [12–14].

*P*. *vivax* is thus considered one of the major challenges for elimination of malaria outside Africa [15]. Better data and tools are urgently required to estimate the *P*. *vivax* hypnozoite reservoir, quantify the relapse burden, better understand potential relapse triggers, and develop the most appropriate public health intervention strategies [15–17].

In malaria-endemic areas of Papua New Guinea (PNG), where four of the five human *Plasmodium* species coexist, *P*. *vivax* predominates as the cause of infection and illness in young children [18,19] and is gradually replaced by *P*. *falciparum* as the main cause of disease in older children and adults [20], although *P*. *vivax* infections remain common throughout childhood and into adulthood, with a prevalence of 13%–36% in cross-sectional surveys conducted in PNG between 2005 and 2010 [21–24]. *P*. *ovale* and *P*. *malariae* are much less common, with a 2010 survey revealing a prevalence of 0.1% and 1.3%, respectively (by quantitative real-time PCR [qPCR]), and are mostly observed in mixed-species infections [22,25]. The PNG standard anti-malarial treatment is artemether-lumefantrine (AL), which acts against the blood stage of the parasite but does not eliminate hypnozoites. In the absence of a nationwide, cost-effective strategy of screening for G6PD, PQ treatment for clearing liver stages, although recommended for G6PD-normal patients, is rarely given. Consequently, relapses are expected to contribute significantly to the high burden and limited seasonality of *P*. *vivax* in PNG children [18,26]. A previous study in PNG children aged 1–5 y observed that presumptive artesunate (7 d) and PQ (14 d, partially supervised) mass treatment to clear hypnozoites reduced the risk of *P*. *vivax* clinical episodes by 28% (*p* = 0.042) compared to only blood-stage treatment and by 33% (*p* = 0.015) compared to no treatment [27]. Although the study used a suboptimal treatment regimen, and thus substantially underestimated the hypnozoite burden, it did highlight the significant challenge relapses pose to successful control and eventual elimination of *P*. *vivax* malaria in PNG and provides a strong rationale for conducting a more comprehensive clinical trial with an in-depth molecular diagnostics component.

Intensified national control efforts have seen the prevalence of light microscopy (LM)–detectable *P*. *vivax* malaria parasites in the general population decrease from 17% in 2006 to 8% in 2010 [25] and to 0.5% in 2014 [28]. Despite these gains, a large reservoir of individuals infected with submicroscopic *P*. *vivax* persists. In a 2010 survey, *P*. *vivax* prevalence by qPCR was 12.8%. Of these infections, 89.6% were asymptomatic, 53.8% were submicroscopic, and 48.9% included *P*. *vivax* gametocytes [25]. Similarly high rates of persistent asymptomatic *P*. *vivax* infection and gametocyte carriage were also found in surveys in Thailand [29,30] and Brazil [31], which have seen substantial recent reductions in transmission.

Mass screening and treatment (MSAT) and mass drug administration (MDA) with artemisinin-based combination therapies have been advocated as important tools to reduce the asymptomatic *P*. *falciparum* reservoir [32,33]. These interventions are also likely to be of great importance for *P*. *vivax* elimination. Significant questions remain, however, none greater than how to best attack the undetectable hypnozoite reservoir.

To address these critical questions we have conducted a randomised double-blind placebo-controlled trial of a highly efficacious PQ treatment regimen in PNG children aged 5–10 y, using detailed molecular diagnostics to directly measure the contribution of relapses to the burden of *P*. *vivax* and *P*. *ovale* infection, disease, and transmission. By using these data in mathematical models, we further estimate the potential effect of MDA with treatment regimens that are part of first-line policy and regimens currently under investigation in clinical trials [34,35] on the prevention of recurrent *P*. *vivax* infections, providing critical evidence-based recommendations for policy-makers.

## Methods

### Ethics Statement

The protocol (S1 Text) received ethical clearance from the PNG Institute of Medical Research Institutional Review Board (0908), the PNG Medical Advisory Committee (09.11), and the Ethics Committee of Basel (237/11) and was conducted in full accordance with the Declaration of Helsinki. The study was retrospectively registered at ClinicalTrials.gov (NCT02143934) on 20 May 2014 due to the fact that it was designed and perceived by the investigating team as a treatment to re-infection cohort study where randomisation of PQ treatment was being done in order to modify exposure and allow a detailed investigation of relapses rather than as a trial to assess the efficacy of this specific treatment schedule. However, following further review, it was decided that the study did indeed fulfil the generally accepted definitions of a clinical trial, and the study was then retrospectively registered at ClinicalTrials.gov. All authors affirm that other trials involving PQ that they are involved in are registered at ClinicalTrials.gov (NCT01837992; NCT02364583).

### Study Site, Design, and Participants

The study was conducted from 17 August 2009 to 20 May 2010 in five village clusters (13 hamlets) of the Albinama and Balif areas of Maprik District, East Sepik Province, PNG, where both *P*. *falciparum* and *P*. *vivax* are hyperendemic and *P*. *vivax* is responsible for the majority of malaria infection and disease in the first 3 y of life [18,22,36]. Malaria transmission is moderately seasonal, peaking in the early wet season from December to March [26]. Health services for the area are provided by the Albinama health sub-centre, the Balif aid post, and a system of village-based health workers operating in all study villages.

**Fig 1:**
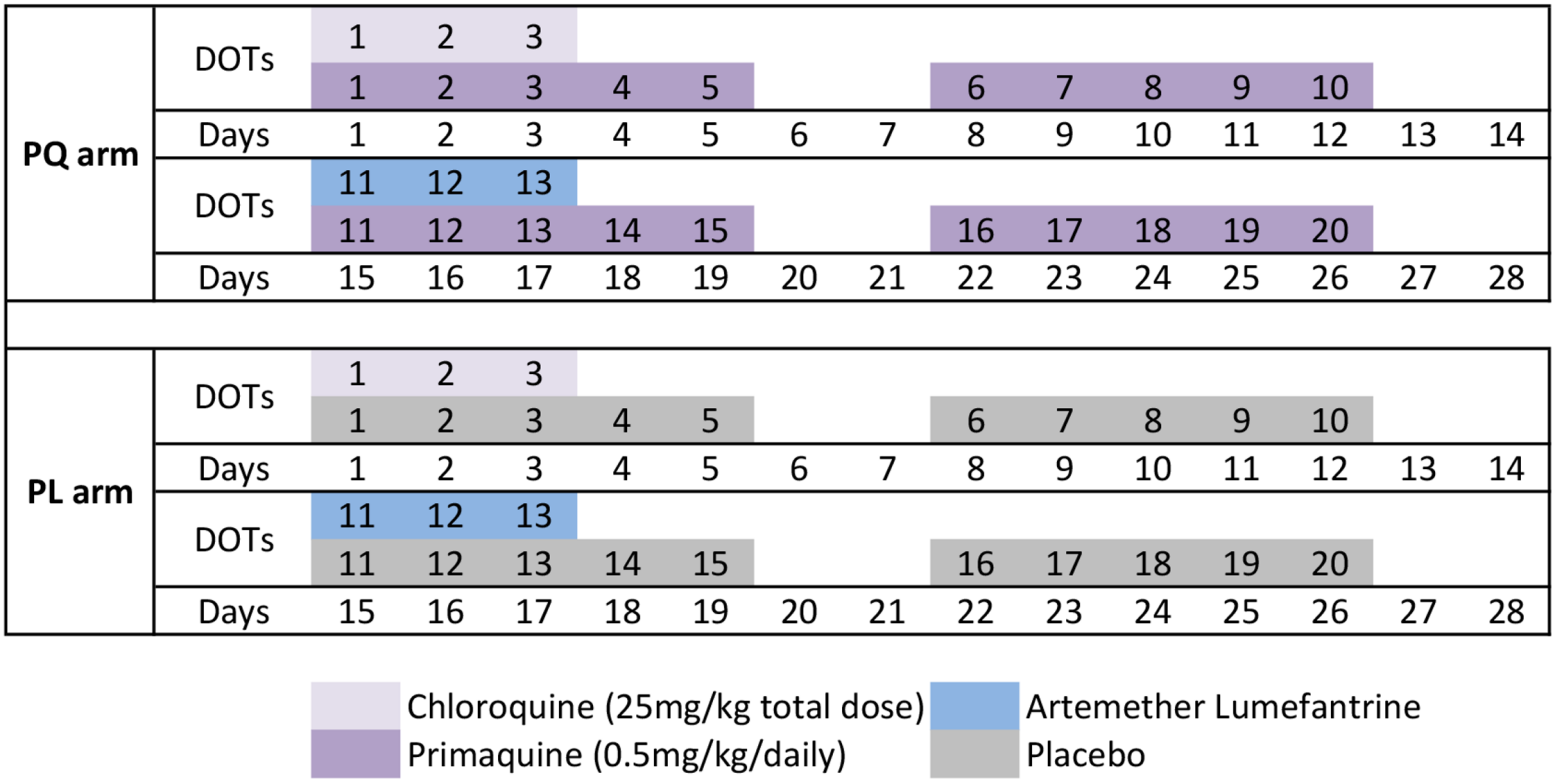
Drug administration schedule. The drug regimen for the PQ and PL treatment arms was administered with direct observation on 20 d (Monday to Friday) over a 4-wk (26-d) period.

Between 17 August and 11 September 2009, 529 children aged 5 to 10 y whose parents provided written informed consent for their participation were screened for inclusion into this parallel double-blind placebo-controlled trial. Children were enrolled if they fulfilled the following criteria: (i) aged 5–10 y (± 3 mo in children without known date of birth, (ii) enrolled at selected elementary schools and permanent residents of the area, (iii) no disability, (iv) no chronic illness, (v) no known allergy to study drugs, (vi) haemoglobin > 50 g/l, (vii) no severe malnutrition (defined by the PNG national guidelines as weight-for-age nutritional Z score < 60th percentile), and (viii) no G6PD deficiency. The inclusion criteria were amended during the study to allow children who were aged 5–6 y but who were not yet enrolled in elementary school to participate in the study. This was necessary to ensure we adequately covered the desired age range of 5–10 y and reached the required sample size of 525 without expanding the geographical area. Five children were excluded on the basis of G6PD deficiency, and 524 were block randomised using a 1:1 allocation ratio to receive 20 d of directly observed treatment (DOT) over 4 wk (26 d) of either (i) chloroquine (CQ) (DOTs 1–3), AL (Coartem) (DOTs 11–13), and PQ (DOTs 1–20; 0.5 mg/kg) or (ii) CQ (DOTs 1–3), AL (DOTs 11–13), and placebo (PL) (DOTs 1–20) (Fig 1). This treatment regimen was deliberately chosen to maximise efficacy, and the dose of each drug was timed such that there would be minimal residual drug by day 0 of the follow-up period (baseline). In order to achieve this, a 4-wk wash-out period was required for CQ, and the 20 d of PQ DOTs were scheduled Monday to Friday of these 4 wk (Fig 1), for ease of direct supervision of every dose. AL was administered because there is documented CQ resistance in PNG, and the PNG national treatment guidelines for *P*. *vivax* are to use AL plus PQ. The administration of AL on DOTs 11–13 (days 15–17) was deliberate so that it wouldn’t interfere with CQ and so that drug levels would have reduced to zero by baseline. The intention was not to trial this unconventional drug regimen as a treatment for implementation, but rather to devise a maximally effective treatment to ensure radical cure in half of the cohort and thus allow a detailed investigation of relapses. Participants, field teams, and investigators were all blinded with respect to treatment allocation.

### Randomisation, Blinding, and Treatment Allocation

After enrolment, children were randomly allocated to the PQ or PL treatment group using a pre-assigned list. Randomisation lists were prepared by an independent statistician using Microsoft Excel and consisted of ID assignments in blocks of six, each block comprising a list of the same six letters in random order. The independent statistician assigned three randomly selected letters to the PQ drug containers and the three other letters to the PL drug containers. The coding document was held by the statistician until completion of the trial. The PQ and PL tablets were identical in size, shape, and colour. The entire study team and principal investigators remained fully blinded for the entire study period.

### Clinical Procedures and Follow-Up

Clinical assessment at enrolment included screening for symptoms of febrile illness, a detailed history of bednet use and recent illness/anti-malarial treatment, and collection of a finger-prick blood sample for assessment of G6PD deficiency using the visual, tube-based G6PD assay (Dojindo Laboratories, Japan), haemoglobin measurement, and later immunological and molecular studies. Children who were febrile were tested using a malaria rapid diagnostic test (RDT) (CareStart Malaria pLDH/HRP2 Combo; AccessBio, US); children with a positive test were treated with AL during DOTs 1–3. DOT 1 was administered at the end of the enrolment visit, with the subsequent 19 DOTs administered daily Monday–Friday over the subsequent 4 wk (Fig 1). All DOTs were supervised by a member of the clinical field team and were co-administered with food and well tolerated [37]. Safety monitoring was performed and reported previously [27]. No specific safety monitoring was conducted during post-intervention follow-up.

**Fig 2:**
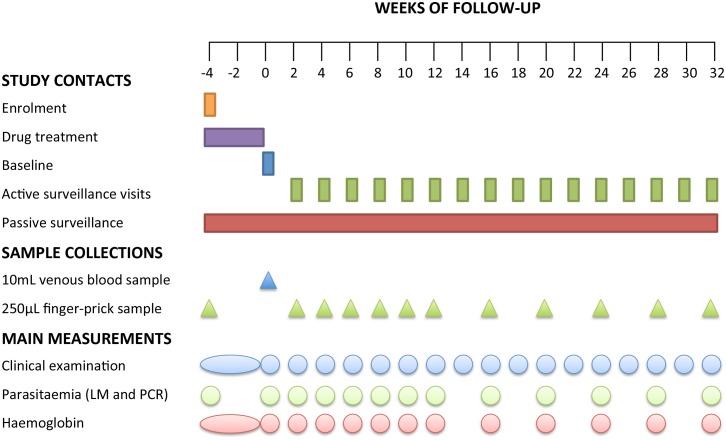
Study design and follow-up schedule. After the drug treatment period, children were actively monitored for infection and illness every 2 wk for the first 12 wk. From week 14 to 32, children were actively monitored for illness every 2 wk and for infection every 4 wk.

Three days after the final DOT (i.e., 4 wk after enrolment), a venous sample was collected and defined as baseline (time point day 0 of the follow-up period). Children were then actively monitored for the presence of febrile symptoms every fortnight for 8 mo. To ensure the capture of information related to any episodes of illness in the 2 wk between visits, passive surveillance measures were implemented at local health centres and aid posts and via the village health worker network. In all symptomatic children, *Plasmodium* spp. infection was initially confirmed using RDT and a 250-μl finger-prick blood sample was collected for confirmation of infection by LM and qPCR. Only symptomatic children who tested positive by RDT and/or LM were treated with AL. PQ was not re-administered for RDT- or LM-confirmed *P*. *vivax* malaria episodes during follow-up. All other illness episodes detected were referred to the local health centre and treated in accordance with PNG treatment guidelines. Finger-prick blood samples were also collected from all children every 2 wk for the first 12 wk and every 4 wk thereafter during the follow-up period (Fig 2). Children were considered lost to follow-up if they permanently relocated outside of the study area or withdrew from the study.

### Laboratory Methods

All blood samples collected during active and passive surveillance were examined by LM and qPCR. Blood films were examined independently by two skilled microscopists, blind to allocated treatment, for 200 thick-film fields (1,000× magnification) before being declared *Plasmodium*-negative. Parasite density was calculated from the number of parasites per 200–500 leucocytes (depending on parasite density) and an assumed leucocyte density of 8,000/μl [38]. Slides discrepant for positivity/negativity, species determination, or density (>2 log difference) were adjudicated by a WHO-certified level 1 (expert) microscopist. Slides were scored as LM-positive for an individual *Plasmodium* species if the species was detected independently by at least two microscopists and/or if subsequent qPCR diagnosis confirmed the presence of the species. Densities were calculated as the geometric mean densities of all positive reads.

Venous blood samples were separated into plasma, peripheral blood mononuclear cells and red cell pellets, which were stored at −80°C, in liquid nitrogen, and at −20°C, respectively. Finger-prick blood samples were separated into plasma and red cell pellets and stored at −80 and −20°C, respectively. DNA was extracted using the FavorPrep 96-Well Genomic DNA Extraction Kit (Favorgen, Taiwan) from the red cell pellet fraction of all samples. *Plasmodium* spp. infections were detected using a generic qPCR to detect all four species, after which species-specific (*P*. *falciparum*, *P*. *vivax*, *P*. *malariae*, and *P*. *ovale*) qPCRs were performed on *Plasmodium*-positive samples [39,40]. The *P*. *ovale* PCR detects both *P*. *ovale curtisi* and *P*. *ovale wallikeri*. In addition, samples positive for *P*. *vivax* by qPCR were tested for gametocytes by *Pvs25* quantitative reverse transcription PCR (qRT-PCR) [39], and individual *P*. *vivax* clones were genotyped by capillary electrophoresis using the molecular marker *msp1F3* [41] to determine the number of genetically distinct *P*. *vivax* blood-stage clones acquired per individual per year at risk, i.e., the molecular force of blood-stage infection (_mol_FOB) [42].

### Statistical Analysis and Modelling

As the primary objective of the study was to determine relapse frequencies, the analysis was by modified intention to treat, excluding children who received fewer than 14 DOTs but including those that received 14–20 DOTs and had incomplete follow-up. For analysis purposes, a clinical episode of *P*. *vivax* or *P*. *falciparum* malaria was defined as febrile illness (current or previous 48 h) plus the presence of *P*. *vivax* or *P*. *falciparum* parasites by LM (any density). The primary trial endpoint was pre-defined as time to first *P*. *vivax* infection after baseline by qPCR. Secondary endpoints included time to first *P*. *vivax* infection after baseline by LM; time to first *P*. *vivax* clinical episode; time to first *P*. *vivax* gametocyte positivity; incidence rate of clinical *P*. *vivax* episodes; incidence rate of genetically distinct *P*. *vivax* infections (_mol_FOB); incidence rate of *P*. *vivax* gametocyte positivity; time to first *P*. *falciparum*, *P*. *malariae*, and *P*. *ovale* infection by qPCR; and time to first *P*. *falciparum* clinical episode. A minimum sample size of 250 children per arm was calculated using hazard-rate-based calculations (log-rank tests) based on the effect (30% reduction in incidence risk in PQ group) observed in [27] with an α–error of 5% and a power of 80% and assuming a 30% increase in time to first infection following additional distributions of long-lasting insecticide-treated nets. Sample sizes were increased by 5% to account for children receiving fewer than 14 doses of PQ or PL.

**Fig 3:**
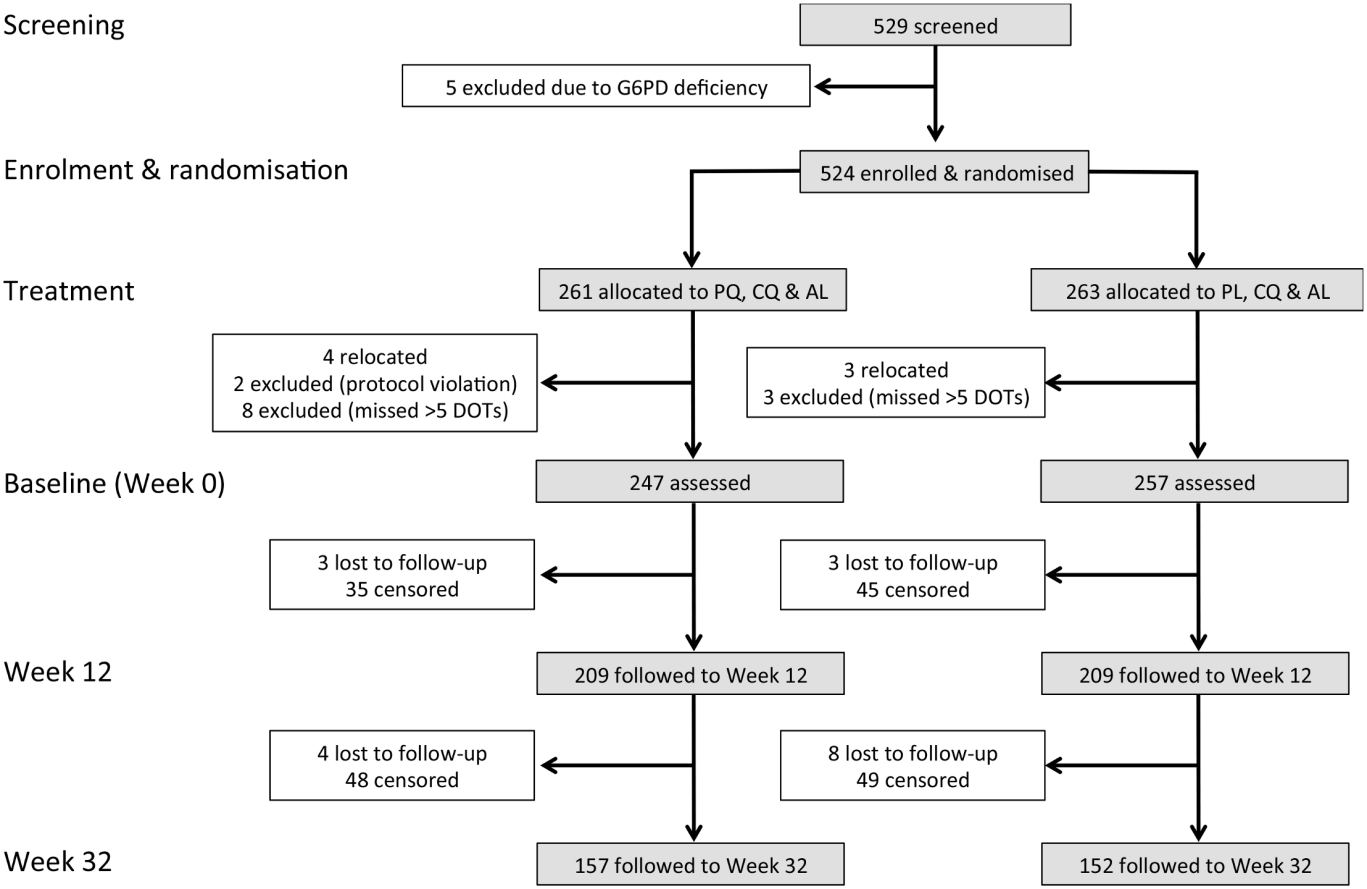
Consort diagram: study design, randomisation, and retention of study participants during follow-up. In the analysis of time to first infection and clinical episode, children were censored on the last day before the first of two consecutive missed clinical visits.

Time to first *Plasmodium* infection or clinical episode and its association with treatment and covariates were modelled using Cox regression, and the proportional hazards (PH) assumption was checked using the test based on the Schoenfeld residuals. For these analyses, time at risk was censored on the last day before the first of two consecutively missed clinical follow-up visits, resulting in the censoring of 177 children (Fig 3). Kaplan-Meier estimates were computed for each endpoint by *Plasmodium* species and method of *Plasmodium* diagnosis. The log-rank test was used to test for differences between survival curves. In all survival analyses, children were considered “at risk” until they reached the endpoint of interest, withdrew, were lost to follow-up, were censored, or completed the study. Village membership was fitted as a fixed effect in the Cox PH model using the following equation:

$$h\left(t\right)\;=h_{0}\left(t\right)\text{ exp}\left(\beta_{\text{1}}x_{1}+{\;\beta }_{2}x_{2}+{\;\beta }_{3}x_{3}+{\;\beta }_{4}x_{4}\right)$$ (1)

where *x*_1_ = treatment, *x*_2_ = age, *x*_3_ = village, and *x*_4_ = infection status by the same *Plasmodium* species at enrolment.

Negative binomial regression (NB reg) models were used to calculate the incidence rate of clinical episodes, _mol_FOB, and *P*. *vivax* gametocyte positivity. In these models, time at risk was calculated for individual children based on the number of attended versus missed visits. If a child was not seen for a consecutive time period of 108 d during the follow-up period, time at risk was censored at the last attended follow-up visit or passive surveillance contact. This resulted in 74 children being censored (44 in PL arm and 30 in PQ arm) rather than the 177 shown in Fig 3. In addition, for the molecular analyses, time at risk was reduced for children who were not seen for consecutive intervals of 42 d by subtracting the days of the missed intervals from the overall individual time at risk. As per PNG treatment guidelines, treatment during the follow-up period was given only if a clinical episode was detected. A potential competing risk could exist if treatment was administered for a clinical episode with one species before the first event for a heterologous species endpoint had occurred. However, since the incidence rate of clinical episodes was very low, this occurred very rarely. It should also be noted that the times at risk for the analyses of different endpoints were not adjusted for the post-treatment prophylactic effects of the aforementioned treatments. Clinical malaria episode incidence rate ratios (IRRs) were derived from models adjusted for treatment, age, and *P*. *vivax* positivity by PCR at enrolment, while the models for _mol_FOB and *P*. *vivax* gametocyte positivity were further adjusted for village of residence. Differences between treatment groups at enrolment were investigated using chi-square and Fisher’s exact tests for categorical characteristics and Student’s *t*-test for normally distributed continuous variables. All tests were two-tailed, and the confidence level was set at 95%. All analyses were performed using R version 3.0.3. [43] and/or Stata version 12 [44].

The effects of MDA and MSAT programmes with anti-malarial drugs currently recommended as first-line treatment on the dynamics of *P*. *vivax* and *P*. *falciparum* transmission were investigated using a mathematical model. The model simulated the impact of a 3-d course of either dihydroartemisinin-piperaquine (DHA-PIP) or CQ to clear blood stages [45], and a 14-d PQ regimen to clear liver stages. The model also simulated the impact of tafenoquine plus CQ treatment, a highly promising short-course anti-relapse therapy that is currently undergoing phase 3 clinical trials [34]. A classical Ross-Macdonald model [46,47] was used to describe the qualitative dynamics of *P*. *falciparum* following treatment of “at risk” populations with drugs for clearing blood-stage infections. This model was extended to incorporate relapse infections of *P*. *vivax* and the effects of PQ treatment for the clearance of liver-stage hypnozoites [48]. In brief, individuals in a population can be susceptible to blood-stage infection with no liver-stage hypnozoites (*S*_0_), infected with blood-stage parasites but no hypnozoites (*I*_0_), susceptible to blood-stage infection but carrying hypnozoites (*S*_L_), or infected with both blood-stage parasites and hypnozoites (*I*_L_). Full details of the deterministic differential equations describing the mathematical model and parameter definitions are provided in S1 Table. The model was also implemented in a stochastic framework with population size 5,000 to capture stochastic variation and the potential for the elimination of transmission.

Details of all methods used in this study are given in S1–S3 Text, S1 Table, and S1 and S2 Figs, and all datasets are available in the Dryad repository: http://dx.doi.org/10.5061/dryad.m1n03 [49].

## Results

### Enrolment and Baseline Characteristics of Children

Of the 524 G6PD-normal children who were randomised to receive PQ/CQ/AL (PQ arm) or PL/CQ/AL (PL arm), a total of 504 children aged 4.8–10.5 y completed at least 14 d of DOT with PQ or PL and remained in the study at baseline (day 0 of follow-up) and were thus actively and passively monitored for 32 wk post-baseline (Fig 3).

No significant differences in demographic characteristics or infection status were observed at enrolment between the treatment groups (Table 1). At baseline (~4 wk after enrolment and commencement of drug treatment), four children were *P*. *vivax*–positive by qPCR (two each in the PL and PQ arms), two children in the PL arm were *P*. *falciparum*–positive, and one of these was also *P*. *vivax*–positive. These children therefore did not contribute to time at risk and were thus excluded from the respective analyses of time to first infection. No *Plasmodium* infections were detected by LM at baseline. During follow-up, 81.6% (2,308/2,827) of scheduled visits were attended by children in the PL arm, and 82.7% (2,248/2,717) in the PQ arm. The average number of all study contacts during follow-up did not differ between the treatment arms (PL arm: 14.0, PQ arm: 14.4, Student’s *t*-test, *p* = 0.25).

**Table 1:** Demographic and clinical characteristics of the cohort at enrolment, classified by allocated treatment.

Characteristic	Total (*n* = 504)	PL Arm (*n* = 257)	PQ Arm (*n* = 247)	*p*-Value
**Male sex**	49.2%	49.8%	48.6%	0.78
**Age (years)**	7.6 ± 1.6	7.7 ± 1.5	7.5 ± 1.6	0.40
**Weight (kilogrammes)**	19.7 ± 3.4	19.7 ± 3.2	19.8 ± 3.6	0.82
**Village**				0.57
Albinama	22.8%	23.0%	22.7%	
Amahup	26.0%	27.3%	24.7%	
Balanga	10.9%	12.5%	9.3%	
Balif	25.6%	24.5%	26.7%	
Bolumita	14.7%	12.8%	16.6%	
**Bednet use**	93.3%	93.4%	93.1%	0.91
**Infection by qPCR** *				
*P*. *vivax*	47.4%	44.8%	50.2%	0.22
*P*. *falciparum*	23.8%	23.7%	23.9%	0.97
*P*. *ovale*	3.4%	1.9%	4.9%	0.07
*P*. *malariae*	14.3%	13.2%	15.4%	0.49
Non–*P*. *vivax*	12.9%	14.0%	11.7%	0.45
**Fever**	14.9%	15.2%	14.6%	0.85
**Haemoglobin (g/l)**	108 ± 13	109 ± 13	108 ± 13	0.78

Data are percentage or mean ± standard deviation. Analysis is by chi-square and Fisher’s exact tests for categorical characteristics and Student’s *t*-test for normally distributed continuous variables.

*Prevalence includes single- and mixed-species infections.

### Risk of *P*. *vivax* Infection during Follow-Up

**Fig 4:**
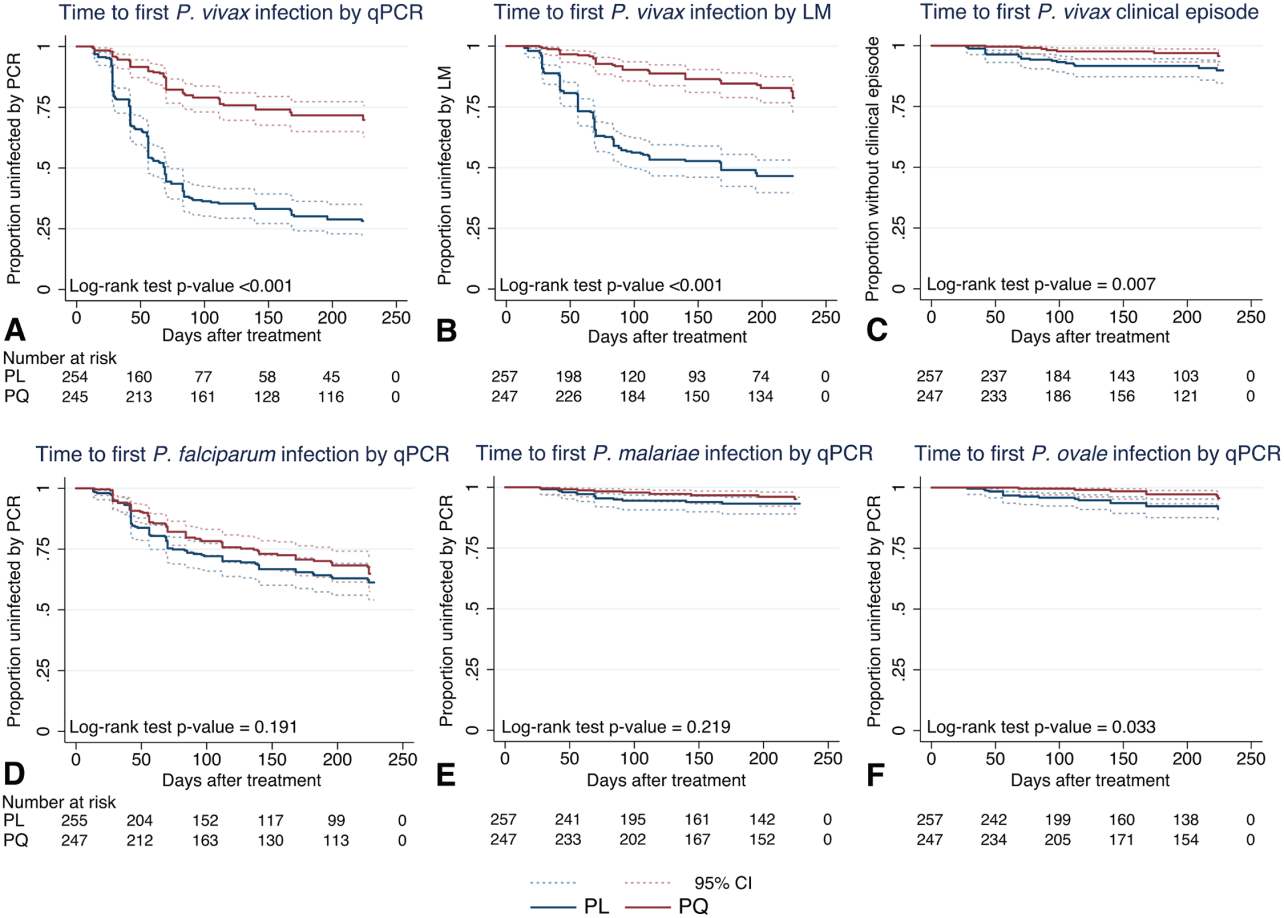
Time to first *Plasmodium* spp. infection and *P*. *vivax* clinical episode. Kaplan-Meier plots showing the time to first (or only) (A) *P*. *vivax* infection by qPCR, (B) *P*. *vivax* infection by LM, (C) *P*. *vivax* clinical episode, (D) *P*. *falciparum* infection by qPCR, (E) *P*. *malariae* infection by qPCR, and (F) *P*. *ovale* infection by qPCR, in the two treatment arms. Dashed lines represent the respective 95% confidence intervals.

The time to first or only *P*. *vivax* blood-stage infection (as detected by qPCR) differed significantly between the two treatment arms: only 25.5% (63/247) of children who received PQ experienced at least one new *P*. *vivax* infection, compared to 65.0% (167/257) in the PL arm (Cox PH, *p <* 0.001; Table 2; Fig 4A). qPCR-positive, recurrent *P*. *vivax* blood-stage infections were detected rapidly in the PL arm, with 31.5% (80/254) of children infected by day 42 compared to only 8.2% (20/245) in the PQ arm (Fig 4A). As expected, LM-positive infections were less common in both arms and were observed later during follow-up (Fig 4B).

**Table 2:** Incidence of first (or only) *P*. *vivax*/*P*. *ovale* re-infections, *P*. *vivax* clinical malaria episodes, and *P*. *vivax* gametocyte positivity in treatment groups during the entire 8-mo follow-up period and during the first 3 mo of follow-up.

Outcome	PL Arm			PQ Arm			Cox Model Estimate*	
	Number of Events	PYR	Incidence Risk	Number of Events	PYR	Incidence Risk	HR (95% CI)	*p*-Value
***P*. *vivax* infections by qPCR**								
Months 0–8	167	63.6	2.62	63	100.6	0.63	0.18 (0.14, 0.25)	<0.001
Months 0–3	152	43.4	3.50	48	54.9	0.87	0.17 (0.12, 0.24)	<0.001
***P*. *vivax* infections by LM**								
Months 0–8	122	83.4	1.46	40	111.7	0.36	0.16 (0.11, 0.24)	<0.001
Months 0–3	104	51.3	2.03	22	58.3	0.38	0.12 (0.07, 0.19)	<0.001
***P*. *vivax* clinical malaria episodes**								
Months 0–8	21	109.0	0.19	7	112.5	0.06	0.25 (0.11, 0.61)	0.002
Months 0–3	15	61.2	0.25	4	60.2	0.07	0.19 (0.06, 0.58)	0.004
***P*. *vivax* gametocyte positivity**								
Months 0–8	99	93.3	1.06	35	112.9	0.31	0.25 (0.17, 0.37)	<0.001
Months 0–3	70	53.9	1.30	16	58.9	0.27	0.18 (0.10, 0.30)	<0.001
***P*. *ovale* infections by qPCR**								
Months 0–8	17	118.0	0.14	7	120.6	0.06	0.31 (0.13, 0.77)	0.011
Months 0–3	10	61.7	0.16	1	60.3	0.02	0.08 (0.01, 0.67)	0.019

*P. *vivax* Cox PH regression model estimates with adjustment for treatment, age, village, and *P*. *vivax* infection status at enrolment. *P*. *ovale* Cox PH regression model estimates adjusted for treatment, age, village, and *P*. *ovale* infection status at enrolment. The number of children considered to be at risk on day 0 was 257 in the PL arm and 247 in the PQ arm, except for *P*. *vivax* infections by PCR, where corresponding numbers at risk were 254 and 245, respectively, since infections on day 0 were considered treatment failures and hence excluded.

HR, hazard ratio; PYR, person-years at risk.

Clearance of liver stages through PQ treatment resulted in an 82%–84% reduction in the risk of recurrent *P*. *vivax* blood-stage infections diagnosed by qPCR (hazard ratio [HR] = 0.18 [95% CI 0.14, 0.25], Cox PH, *p <* 0.001) and LM (HR = 0.16 [95% CI 0.11, 0.24], Cox PH, *p <* 0.001) compared to PL (Table 2). This increased to an 83%–88% reduction in risk when only the first 3 mo of follow-up were considered (qPCR: HR = 0.17 [95% CI 0.12, 0.24], Cox PH, *p <* 0.001; LM: HR = 0.12 [95% CI 0.07, 0.19], Cox PH, *p <* 0.001; Table 2).

There was considerable heterogeneity in the prevalence and risk of *P*. *vivax* infection across the study villages. The risk of first *P*. *vivax* infection (by qPCR and LM) differed significantly among children living in different villages, with the highest risk in Bolumita (qPCR: HR = 4.70 [95% CI 3.14, 7.02], Cox PH, *p <* 0.001; LM: HR = 4.62 [95% CI 2.92, 7.30], Cox PH, *p <* 0.001, reference village Albinama) and Balanga (qPCR: HR = 2.33 [95% CI 1.53, 3.55], Cox PH, *p <* 0.001; LM: HR = 1.70 [95% CI 1.03, 2.81], Cox PH, *p* = 0.04, reference village Albinama). There was, however, no interaction between village and treatment effect. The risk of first *P*. *vivax* blood-stage infection diagnosed by qPCR after treatment was not significantly associated with age (qPCR: HR = 0.95 [95% CI 0.87, 1.03], Cox PH, *p* = 0.19); however, there was a 15% reduction in the risk of having at least one LM-patent *P*. *vivax* infection with each additional year of life (LM: HR = 0.85 [95% CI 0.77, 0.95], Cox PH, *p* = 0.003). As with village, there was no interaction between age and treatment effect.

When all *P*. *vivax* infections in a child were genotyped to identify new infections in the context of ongoing multiple clone infections, PQ treatment was associated with a 77% reduction in the incidence of genetically distinct *P*. *vivax* blood-stage clones (_mol_FOB) compared to PL (PQ arm: _mol_FOB = 1.62/y, PL arm: _mol_FOB = 4.74/y, IRR = 0.23 [95% CI 0.18, 0.31], NB reg, *p <* 0.001; Table 3). The effect of the PQ treatment on _mol_FOB was substantially larger in the first 3 mo (IRR = 0.21 [95% CI 0.15, 0.28], *p <* 0.001) than in months 4–8 (IRR = 0.34 [95% CI 0.24, 0.48], NB reg, *p <* 0.001; Table 3).

**Table 3:** Incidence rate of *P*. *vivax* clinical malaria of any density, genetically distinct *P*. *vivax* blood-stage clones, and gametocyte positivity in treatment groups during the entire 8-mo follow-up period and separately for months 0–3 and 4–8 of follow-up.

Outcome	PL Arm			PQ Arm			Model Estimate*	
	Number of Events	PYR	Incidence Rate	Number of Events	PYR	Incidence Rate	IRR (95% CI)	*p*-Value
***P*. *vivax* clinical malaria episode—any density**								
Months 0–8	23	139.8	0.16	11	139.6	0.08	0.43 (0.21, 0.90)	0.026
Months 0–3	15	55.9	0.30	5	54.3	0.09	0.31 (0.11, 0.86)	0.025
Months 4–8	7	83.0	0.08	5	83.1	0.06	0.59 (0.17, 2.08)	0.410
**Genetically distinct *P*. *vivax* blood-stage clones (**_**mol**_ **FOB)**								
Months 0–8	653	122.1	4.74	196	120.6	1.62	0.23 (0.18, 0.31)	<0.001
Months 0–3	430	55.5	7.75	102	53.7	1.90	0.21 (0.15, 0.28)	<0.001
Months 4–8	221	66.5	3.32	94	66.0	1.42	0.34 (0.24, 0.48)	<0.001
***P*. *vivax* gametocyte positivity**								
Months 0–8	202	122.1	1.65	63	120.6	0.52	0.27 (0.19, 0.38)	<0.001
Months 0–3	101	55.5	1.82	20	53.7	0.37	0.18 (0.11, 0.30)	<0.001
Months 4–8	100	66.5	1.50	42	66.0	0.64	0.37 (0.24, 0.57)	<0.001

*The time at risk is calculated independently for each individual for each time range, with applicable study censoring for either clinical or PCR detection. Clinical malaria episode IRRs are derived from a NB reg adjusted for treatment, age, and *P*. *vivax* positivity by PCR at enrolment. Other IRRs are derived from a NB reg model adjusted for treatment, age, village, and *P*. *vivax* positivity by PCR at enrolment. The number of children considered at risk on day 0 was 245 in the PL arm and 253 in the PQ arm. The number of children considered at risk on study day 96 was 207 in the PQ arm and 205 in the PL arm.

PYR, person-years at risk.

In addition, PQ treatment was associated with a 75% reduction in the hazard of becoming positive for *P*. *vivax* gametocytes by *Pvs25* qRT-PCR compared to PL (HR = 0.25 [95% CI 0.17, 0.37], Cox PH, *p <* 0.001; Table 2). The incidence rate of *P*. *vivax* gametocytes (defined as the number of samples positive for *Pvs25* qRT-PCR during follow-up) was more strongly reduced in the PQ arm compared to the PL arm in the first 3 mo (IRR = 0.18 [95% CI 0.11, 0.30], *p <* 0.001) than in the subsequent 5 mo (months 4–8: IRR = 0.37 [95% CI 0.24, 0.57], NB reg, *p <* 0.001; Table 3).

Irrespective of treatment group, clinical *P*. *vivax* malaria episodes were rare, with only 28 children experiencing one or more clinical *P*. *vivax* episodes during the 32-wk follow-up period (incidence risk: 0.19/child/y in PL arm and 0.06/child/y in PQ arm; Table 2). The clearance of hypnozoites by PQ treatment was associated with an 81% reduction compared to PL in the hazard of experiencing a *P*. *vivax* clinical episode of any density in the first 3 mo of follow-up (HR = 0.19 [95% CI 0.06, 0.58], Cox PH, *p* = 0.004; Table 2; Fig 4C) and a 75% reduction in the entire follow-up period (HR = 0.25 [95% CI 0.11, 0.61], Cox PH, *p* = 0.002; Table 2).

### Risk of Non–*P*. *vivax* Malaria Infection during Follow-Up

Although the number of *P*. *ovale* infections diagnosed by PCR in either arm was low (incidence risk: both arms: 0.10 infections/child/y, PQ arm: 0.06, PL arm: 0.14), PQ treatment was associated with a 92% reduction compared to PL in the risk of *P*. *ovale* blood-stage infection diagnosed by qPCR in the first 3 mo of follow-up (HR = 0.08 [95% CI 0.01, 0.67], Cox PH, *p* = 0.019) and a 69% reduction in the entire 8-mo of follow-up period (HR = 0.31 [95% CI 0.13, 0.77], Cox PH, *p* = 0.011; Fig 4F; Table 2).

There was no significant association between PQ treatment and time to first (or only) *P*. *falciparum* blood-stage infection by qPCR (Cox PH, *p* = 0.104; Fig 4D; Table 4) or LM (Cox PH, *p* = 0.706; Table 4), and no effect of PQ treatment on time to first (or only) *P*. *falciparum* clinical episode during the 8 mo of follow-up (Cox PH, *p* = 0.333; Table 4). There was no significant association between PQ treatment and time to first (or only) *P*. *malariae* blood-stage infection by qPCR in the first 3 mo of follow-up (HR = 0.36 [95% CI 0.13, 1.02], Cox PH, *p* = 0.055) or during the entire follow-up period (HR = 0.55 [95% CI 0.24, 1.26], Cox PH, *p* = 0.157; Fig 4E; Table 4).

**Table 4:** Incidence of first (or only) *P*. *falciparum/P*. *malariae* re-infection and *P*. *falciparum* clinical malaria in treatment groups in the entire follow-up period.

Outcome (Months 0–8)	PL Arm			PQ Arm			Cox Model Estimate*	
	Number of Events	PYR	Incidence Risk	Number of Events	PYR	Incidence Risk	HR (95% CI)	*p*-Value
*P*. *falciparum* infections by PCR	85	96.0	0.88	72	101.0	0.71	0.77 (0.56, 1.06)	0.104
*P*. *falciparum* infections by LM	52	108.8	0.48	53	107.1	0.50	0.93 (0.63, 1.37)	0.706
*P*. *falciparum* clinical malaria episodes	21	109.4	0.19	27	105.4	0.26	1.33 (0.75, 2.36)	0.333
*P*. *malariae* infections by PCR	15	118.2	0.13	9	119.4	0.07	0.55 (0.24, 1.26)	0.157

*P. *falciparum* Cox PH regression model estimates adjusted for treatment, age, village, and *P*. *falciparum* infection status at enrolment. *P*. *malariae* Cox PH regression model estimates adjusted for treatment, age, village, and *P*. *malariae* infection status at enrolment. The number of children considered to be at risk on day 0 was 257 in the PL arm and 247 in the PQ arm, except for *P*. *falciparum* infections by PCR, where corresponding numbers at risk were 255 and 247, respectively, since infections on day 0 were considered treatment failures and hence excluded.

PYR, person-years at risk.

### Implications for Malaria Control and Elimination Strategies

**Fig 5:**
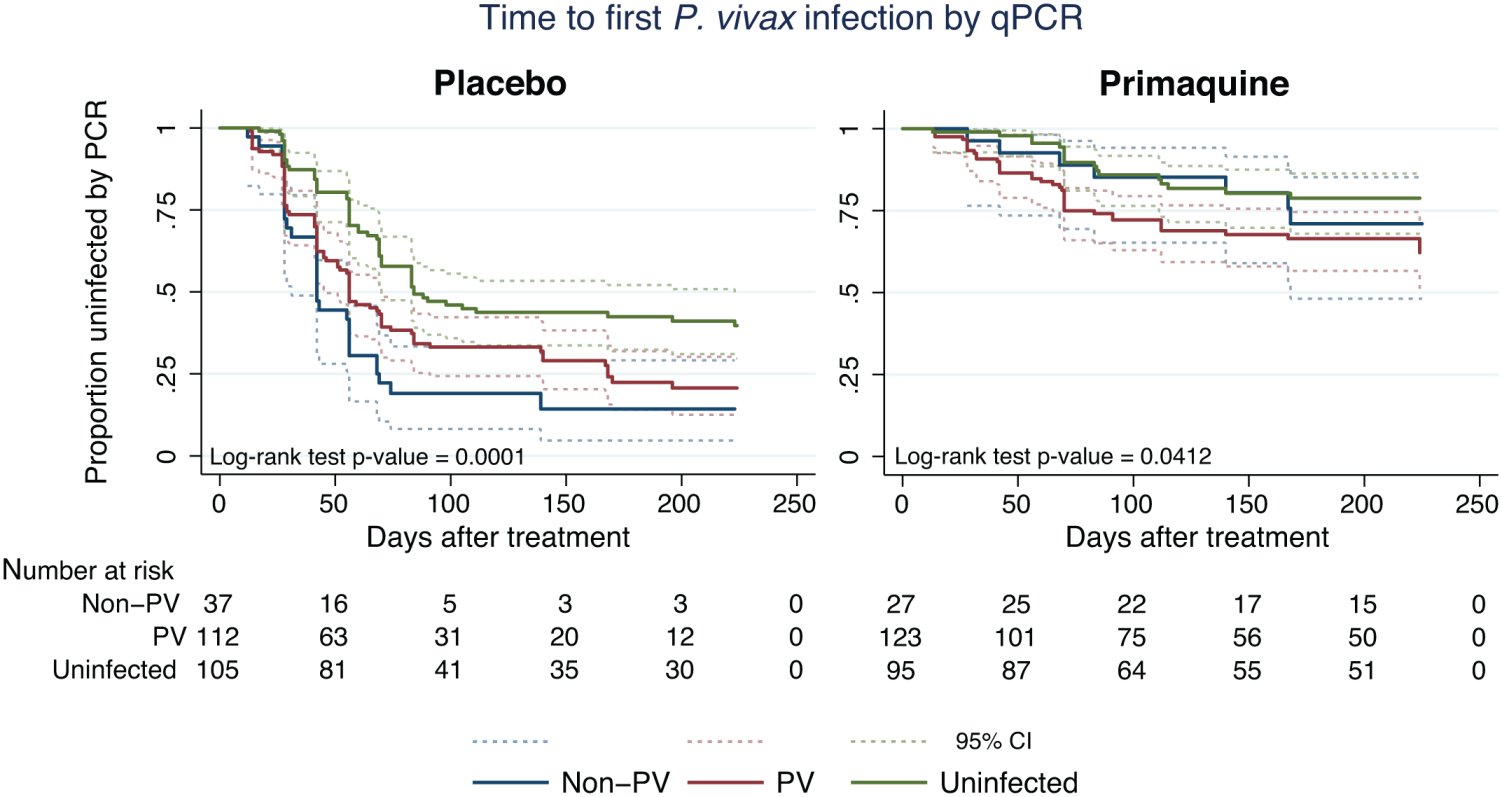
Kaplan-Meier plots showing the time to first (or only) *P*. *vivax* infection by qPCR in the PL and PQ arms, stratified by *Plasmodium* infection status at enrolment. Dashed lines represent the respective 95% confidence intervals. PV, *P*. *vivax*.

MSAT interventions target only individuals with detectable blood-stage parasitaemia. In the current cohort, 47.4% (239/504) of children had a *P*. *vivax* infection (single or mixed species) at enrolment, and another 12.9% (65/504) were positive with non–*P*. *vivax* blood-stage infections (Table 1). In the absence of PQ treatment, children with no patent infections at enrolment were significantly less rapidly re-infected with *P*. *vivax* (55.2% [58/105] became infected during 8 mo of follow-up) than those that had patent *P*. *vivax* (70.5% [79/112]) or *P*. *falciparum/P*. *malariae/P*. *ovale* infections (81.1% [30/37], log-rank test, *p* < 0.001; Fig 5; Table 5). In the PQ group, children with no patent infections were also less likely to be re-infected (17.9% [17/95]) than those in the other two groups (*P*. *vivax* patent infection: 31.7% [39/123], non–*P*. *vivax* patent infection: 25.9% [7/27], log-rank test, *p* = 0.0412; Fig 3; Table 5), indicating that children with no patent infections were more likely to live in low-transmission areas. PQ treatment was therefore equally efficient in preventing recurrent *P*. *vivax* infections in children with *P*. *vivax*, non–*P*. *vivax*, or no blood-stage infection at enrolment (Cox regression, adjusted for age and village of residence: likelihood ratio = 3.87, df = 2, *p* = 0.14 for treatment-by-infection status interaction).

**Table 5:** *P*. *vivax* re-infection by qPCR in treatment groups during the entire 8 mo follow-up period and during the first 3 mo of follow-up, stratified by *P*. *vivax* infection status at enrolment.

Infection Status at Enrolment	PL Arm			PQ Arm			Cox Model Estimate*	
	Number of Events	PYR	Incidence Risk	Number of Events	PYR	Incidence Risk	HR (95% CI)	*p*-Value
**Non–*P*. *vivax* infection (*n* = 65)**								
Months 0–8	30	6.3	4.78	7	12.7	0.55	0.11 (0.04, 0.26)	<0.001
Months 0–3	29	5.1	5.73	4	6.6	0.61	0.08 (0.03, 0.24)	<0.001
***P*. *vivax* infection (*n* = 239)**								
Months 0–8	79	24.9	3.18	39	46.4	0.84	0.21 (0.14, 0.32)	<0.001
Months 0–3	71	18.0	3.95	32	26.3	1.22	0.21 (0.14, 0.33)	<0.001
**Uninfected (*n* = 200)**								
Months 0–8	58	32.4	1.79	17	41.4	0.41	0.19 (0.11, 0.34)	<0.001
Months 0–3	52	20.4	2.55	12	22.0	0.55	0.15 (0.08, 0.29)	<0.001

*Cox PH regression model estimates adjusted for treatment, age, village, and *P*. *vivax* infection status at enrolment. The number of children considered to be at risk on day 0 was 254 in the PL arm and 245 in the PQ arm.

PYR, person-years at risk.

**Fig 6:**
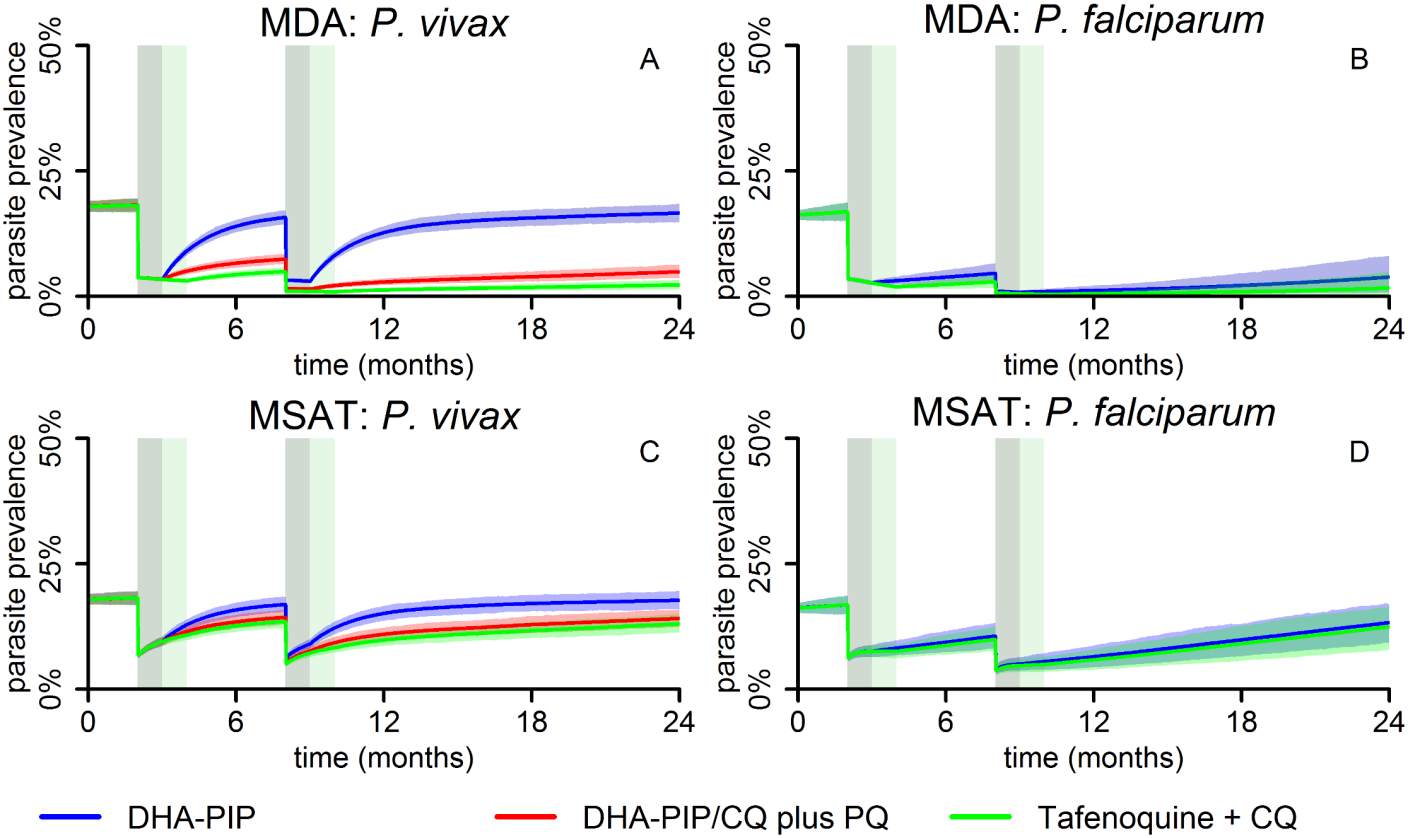
Mathematical-model-based predictions of impact of MDA and MSAT with either blood-stage drugs only or blood- plus liver-stage drugs on the population prevalence of *P*. *vivax* and *P*. *falciparum* infections. The effect of two rounds (6 mo apart) of MDA (A and B) or MSAT (C and D) with anti-malarial drugs at 80% coverage on *P*. *vivax* (A and C) and *P*. *falciparum* (B and D) blood-stage parasite prevalence, as predicted by a stochastic model in a human population of size 5,000. The lines represent the mean of 1,000 repeat simulations, and the shaded areas represent the envelopes containing 95% of stochastic simulations. The grey and green shaded bars denote the duration of prophylactic protection for DHA-PIP/CQ and tafenoquine, respectively, after each treatment round. DHA-PIP and CQ were assumed to be administered as part of a 3-d regimen, providing prophylaxis for 1 mo. PQ was assumed to be administered as part of a 14-d regimen, providing prophylaxis for 15 d. Tafenoquine was assumed to be administered via a single dose, providing prophylaxis for 2 mo.

The potential effect of MDA and MSAT with either blood-stage drugs only or blood- plus liver-stage drugs on the population prevalence of *P*. *vivax* and *P*. *falciparum* infections was further investigated using a basic stochastic transmission model. Administration of blood-stage drugs (DHA-PIP or CQ) was assumed to clear existing blood-stage infections and provide a 4-wk period of prophylactic protection against new infections, tafenoquine plus CQ was assumed to clear blood- and liver-stage infections and provide 8 wk of causal prophylactic protection (for full model details please refer to S1 Table). Fig 6 illustrates the predicted qualitative dynamics of *P*. *falciparum* and *P*. *vivax* transmission following two rounds of MDA (Fig 6A and 6B) or MSAT (Fig 6C and 6D) interventions with 80% coverage, separated by 6 mo. The interventions are predicted to cause a sharp decline in prevalence, followed by a gradual return to pre-intervention levels.

MDA is predicted to achieve much larger reductions in prevalence than MSAT, mostly due to the proportion of infections missed by the MSAT programme because of imperfect diagnostic sensitivity, but also because of the prophylactic protection in treated but uninfected individuals under the MDA programme.

The initial reductions achieved by each of the interventions against *P*. *falciparum* and *P*. *vivax* are the same; however, when only blood-stage drugs are administered, a rapid rebound in *P*. *vivax* prevalence is predicted due to the hypnozoite reservoir, which is left unaffected by the treatment. Notably, *P*. *vivax* levels are predicted to return to pre-intervention levels within 6 mo post-intervention for both the MDA and MSAT interventions (blue curves in Fig 6A and 6C), in contrast to *P*. *falciparum*, where, following an MDA programme, prevalence is predicted to remain below pre-intervention levels for a sustained period of time (Fig 6B).

In the stochastic transmission model, only the addition of 8-aminoquinolines to a regimen of blood-stage drugs targets the hypnozoite reservoir and prevents the rapid resurgence of *P*. *vivax* blood-stage infections caused by relapses. Consequently, interventions with PQ are shown to result in a sustained reduction of the *P*. *vivax* burden (red curves in Fig 6A and 6C). Especially in the case of MDA (red curve, Fig 6A), *P*. *vivax* parasite prevalence is predicted to remain very low (<10%) during the modelled 16 mo post-intervention. Tafenoquine is estimated to be more effective than PQ at reducing prevalence because of the higher level of efficacy and the longer duration of causal prophylaxis [34].

## Discussion

By randomising children to receive either a blood- and liver-stage treatment of PQ/CQ/AL (PQ arm) or a blood-stage only treatment of PL/CQ/AL (PL arm), and thereby selectively removing hypnozoites from half of the children in the cohort, this study confirms that relapses from long-lasting liver stages account for four out of five *P*. *vivax* infections and three out of five *P*. *ovale* infections in PNG children aged 5–10 y living in an area with hyperendemic transmission. A transmission model estimated that MSAT with blood- and liver-stage treatment or MDA with blood-stage treatment alone would have only a transient effect on *P*. *vivax* transmission levels, while MDA with blood- and liver-stage treatment is predicted to be a highly effective strategy for *P*. *vivax* elimination.

The risk of having at least one PCR-detectable *P*. *vivax* infection during 8 mo of follow-up was reduced by 82% by PQ treatment. This reduction is substantially larger than the 44% reduction observed in an earlier study [27] that used a 30% lower dose of PQ combined with 7 d of artesunate, confirming that this earlier treatment regimen was not fully effective in preventing relapses. PQ also reduced the incidence of genetically distinct blood-stage infections (_mol_FOB) [42] in the first 3 mo of follow-up by a similar amount (79%), indicating that relapses accounted for ~4/5 of all *P*. *vivax* infections in PNG children. Although the effect of the PQ treatment decreased with time since treatment, the incidence of new infections in the PQ arm was nevertheless still reduced by more than half (66%) after 3 mo of follow–up. This “wash-out” of the PQ effect is likely due to relapses activating rapidly in PNG [7] and the hypnozoite reservoir being replenished through continued exposure to new infected mosquito bites. The relatively sustained effect of the PQ treatment does however indicate that even in tropical *P*. *vivax* strains, a substantial proportion of hypnozoites remain dormant for three or more months.

Children who were not treated with PQ were also approximately four times more often positive for *P*. *vivax* gametocytes than children who were treated with PQ. Since even low-density *P*. *vivax* infections can infect mosquitoes [50–52], it is thus likely that relapsing infections are the primary source of *P*. *vivax* transmission.

PNG children acquire immunity to *P*. *vivax* rapidly [18], and, as shown in earlier studies in the same age group [20], clinical *P*. *vivax* episodes are rare. Nevertheless, PQ reduced the incidence of clinical *P*. *vivax* episodes by 69% in the first 3 mo after treatment, indicating that relapses can cause clinical episodes even in individuals with moderate levels of acquired immunity. Several studies have shown that relapses are often genetically distinct from the initial clinical episode [53,54]. These genetically diverse relapses may either be meiotic siblings [55] or originate from a previous (mosquito) bite and are likely to result in a higher risk of clinical illness in relapsing infection than that seen in malaria therapy patients infected twice with the same strain [10].

PQ treatment reduced the risk of PCR-detectable *P*. *ovale* infections by 92% in the first 3 mo of follow-up and by 69% in the entire follow-up period. This confirms that relapses account for a considerable portion of infections from this less prevalent species of relapsing malaria, and likely also sustain *P*. *ovale* transmission. This is relevant not only in PNG, but also in sub-Saharan Africa, where *P*. *ovale* prevalence can reach 4%–10% (by LM) in areas of west and central Africa [56]. We observed no significant association of PQ treatment with risk of PCR-detectable infections from the non-relapsing species *P*. *falciparum*. In the PQ arm, there was a non-significant (*p* = 0.055; only 24 total events) reduction in the risk of *P*. *malariae* infections in the first 3 mo of follow-up compared to the PL arm. Although *P*. *malariae* is not thought to form hypnozoites in the liver, chronic or relapsing infections of *P*. *malariae* up to 20 y after a person has left an endemic area have been documented [57,58]. Although it is currently not known how and where *P*. *malariae* infections can remain dormant for such extensive periods of time, our data suggest that such longer-lived *P*. *malariae* stages may be susceptible to PQ treatment. Larger clinical trials involving both symptomatic and asymptomatic *P*. *malariae* infections would be required to confirm the prevention of recurrent *P*. *malariae* infections by PQ treatment.

Given the very large contribution of relapses to the burden of *P*. *vivax* and *P*. *ovale* infection, illness, and transmission, it is essential that all *P*. *vivax*– and *P*. *ovale*–infected individuals receive therapy that is effective against both blood stages and relapses. PNG national standard treatment guidelines recommend treating confirmed *P*. *vivax* or *P*. *ovale* cases with 0.25 mg/kg PQ for 14 d; however, this recommendation is not being consistently implemented due to lack of access to point-of-care tests to screen for G6PD deficiency.

Interestingly, PQ was not only effective in preventing recurrent *P*. *vivax* infection in children who had PCR-detectable *P*. *vivax* infections but was equally effective in children with non–*P*. *vivax* infections and even those without any *Plasmodium* spp. infections at enrolment. All currently available malaria diagnostic tests identify only active blood-stage infections. They can thus not identify people who have *P*. *vivax* hypnozoites in their livers but are free of blood-stage infection. The rapid appearance of *P*. *vivax* infections and significant reduction in rates of recurrent infections in PQ-treated qPCR-negative children indicate that the number of PNG children without blood-stage infections who carry *P*. *vivax* hypnozoites is comparable to the number of children with *P*. *vivax* infections. The presence of active *P*. *vivax* blood-stage infection is thus a poor predictor for the risk of *P*. *vivax* relapse.

The rapid recurrence of *P*. *vivax* infections after treatment of *P*. *falciparum* infections has been observed in numerous *P*. *falciparum* in vivo drug efficacy trials, both in PNG [45,59] and elsewhere [60]. It has therefore been argued that anti-relapse therapy should be administered for all patients with malaria in regions of *P*. *vivax* co-endemicity [60]. Our results clearly support this recommendation and also extend it to asymptomatic parasite carriers.

With the renewed drive to eliminate malaria, the role of asymptomatic and/or submicroscopic infections in sustaining malaria transmission has become a major focus [15,61,62]. An increasing number of studies show that these infections contribute significantly to both *P*. *falciparum* and *P*. *vivax* transmission at all levels of endemicity [50–52,63,64]. As first noted by Robert Koch in 1900 during studies in then German New Guinea, the control of these infections is essential if elimination is to be achieved rapidly [65].

MSAT and MDA with artemisinin-based combination therapies are two interventions aimed at reducing the asymptomatic reservoir [32,66,67]. Although the simple model implemented in the present study agrees with the findings from more detailed *P*. *falciparum* models [67] that an MSAT programme with a highly sensitive diagnostic test such as qPCR can effectively reduce *P*. *falciparum* transmission, field trials have shown that MSAT with a less sensitive diagnostic test such as an RDT has limited or no effect on transmission [68–70]. As mass screening by PCR is difficult to implement, focalised MDA may be a more practical approach [71].

Our modelling predicted that MSAT will have only limited effectiveness for *P*. *vivax* even if conducted with a sensitive molecular diagnostic test and including an anti-liver-stage treatment since it will not target the blood-stage-negative population harbouring hypnozoites. MDA, on the other hand, is predicted to be highly effective in reducing the burden of future *P*. *vivax* infections but only if conducted with anti-blood- and anti-liver-stage treatment. Thus, effective control of *P*. *vivax* with anti-malarial drugs will require the inclusion of a treatment to attack the hypnozoite reservoir and will require mass administration regardless of the presence of blood-stage infections to target the undetectable parasite reservoir.

Currently, PQ is the only licensed drug with activity on the hypnozoite stage capable of preventing relapses [72]. However, due to its association with haemolysis in individuals with G6PD deficiency [72,73] and its long dosing schedule (up to 14 d), this drug is not in widespread use in many endemic areas, and WHO currently advises against PQ treatment without prior G6PD deficiency testing. In the absence of a reliable and affordable point-of-care G6PD test, the routine use of PQ for treatment thus remains a challenge in many endemic regions. The challenges of implementing MDA with PQ are even larger, although not insurmountable [74]. A new generation of G6PD tests are being developed [75,76], and the arrival of tafenoquine, a long-acting 8-aminoquinoline that can be given as a single dose [34,76], will not only make MDA logistically simpler, but our modelling also predicts that MDA with tafenoquine will be more effective than regimens using PQ. Nevertheless, the development of alternative anti-hypnozoite treatments remains an important research priority for the elimination of *P*. *vivax* [16].

The treatment schedule used in the present study was optimised for the best possible anti-hypnozoite activity. The results from the clinical trial are therefore not directly indicative of the effectiveness of an MDA programme with standard 14-d PQ dosing. As a consequence, we assumed a significantly lower PQ efficacy (75%) in our model than we found in our study. Similarly, our results refer to the fast-relapsing *P*. *vivax* strains that are found in relatively highly endemic areas in the southwest Pacific, southeast Asia, and parts of Amazonia [77]. Further studies in areas with lower transmission levels and longer relapse intervals will be required to determine the generalisability of our finding of relapses causing 80% of infections and to extend the model predictions to other geographical regions and transmission settings.

In conclusion, this study demonstrates that relapsing infections are the overwhelming source (i.e., ~80%) of not only *P*. *vivax* blood-stage infections in children but also clinical episodes, and that they contribute substantially to maintaining transmission. Given the very ambitious timelines set by political leaders of *P*. *vivax*–endemic regions and limited funding, it is essential that scarce resources available for both *P*. *vivax* research and elimination be optimally allocated. The development of novel anti-hypnozoite drugs and interventions that can specifically target the hypnozoite reservoir are of highest priority. However, by predicting that MSAT programmes will not be effective in reducing the burden of *P*. *vivax* in affected populations, the models presented here also suggest that it is not worthwhile investigating or implementing MSAT programmes in *P*. *vivax*–endemic countries. Instead, efforts should now be directed towards the development of approaches for MDA programmes targeting areas and risk groups with confirmed local transmission.

## Supporting Information

S1 Fig Schematic representation of the *P*. *vivax* transmission model and the associated system of differential equations. *S*_0_ denotes fully susceptible humans, *I*_0_ denotes individuals with blood-stage infection, *S*_L_ denotes individuals with liver-stage infection with hypnozoites, and *I*_L_ denotes individuals with blood-stage infection and liver-stage infection with hypnozoites.
(TIF)

S2 Fig The effect of two rounds of MDA and MSAT with anti-malarial drugs on *P*. *vivax* and *P*. *falciparum* blood-stage parasite prevalence, as predicted by the deterministic model. The grey and green shaded bars denote the duration of prophylactic protection for DHA-PIP/CQ and tafenoquine, respectively, after each treatment round. DHA-PIP and CQ were assumed to be administered as part of a 3-d regimen providing prophylaxis for 1 mo. PQ was assumed to be administered as part of a 14-d regimen providing prophylaxis for 15 d. Tafenoquine was assumed to be administered via a single dose providing prophylaxis for 2 mo.
(TIF)

S1 Table Description of parameters for the malaria transmission model. (PDF)

S1 Text Trial protocol. (PDF)

S2 Text CONSORT checklist. (PDF)

S3 Text Overview of the malaria transmission model. (PDF)

## Abbreviations

AL: artemether-lumefantrine
CQ: chloroquine
DHA-PIP: dihydroartemisinin-piperaquine
DOT: directly observed treatment
G6PD: glucose-6-phosphate dehydrogenase
HR: hazard ratio
IRR: incidence rate ratio
LM: light microscopy
MDA: mass drug administration
_mol_FOB: molecular force of blood-stage infection
MSAT: mass screening and treatment
NB reg: negative binomial regression
PH: proportional hazards
PL: placebo
PNG: Papua New Guinea
PQ: primaquine
qPCR: quantitative real-time PCR
qRT-PCR: quantitative reverse transcription PCR
RDT: rapid diagnostic test

## References

[1] World Health Organization. World malaria report 2014. Geneva: World Health Organization; 2014.

[2] Cotter C, Sturrock H, Hsiang M, Liu J, Phillips A, Hwang J, et al The changing epidemiology of malaria elimination: new strategies for new challenges. Lancet. 2013;382:900–911. 10.1016/S0140-6736(13)60310-4 23594387 PMC10583787

[3] Pan American Health Organization, World Health Organization. Central America and Hispaniola seek to eliminate malaria by 2025. 21 5 2013 Geneva: World Health Organization.

[4] Asia Pacific Leaders Malaria Alliance. East Asia Summit adopts unprecedented regional malaria goal. 14 11 2014 Mandaluyong City (Philippines): Asia Pacific Leaders Malaria Alliance.

[5] Mueller I, Galinski MR, Baird JK, Carlton JM, Kochar DK, Alonso PL, et al Key gaps in the knowledge of *Plasmodium vivax*, a neglected human malaria parasite. Lancet Infect Dis. 2009;9:555–566. 10.1016/S1473-3099(09)70177-X 19695492

[6] Baird JK. Eliminating malaria—all of them. Lancet. 2010;376:1883–1885. 10.1016/S0140-6736(10)61494-8 21035840

[7] White NJ. Determinants of relapse periodicity in *Plasmodium vivax* malaria. Malar J. 2011;10:297 10.1186/1475-2875-10-297 21989376 PMC3228849

[8] Carson P, Flanagan C, Ickes C, Alving A. Enzymatic deficiency in primaquine-sensitive erythrocytes. Science. 1956;124:484–485. 13360274 10.1126/science.124.3220.484-a

[9] White NJ, Imwong M. Relapse. Adv Parasitol. 2012;80:113–150. 10.1016/B978-0-12-397900-1.00002-5 23199487

[10] Mckenzie FE, Jeffery GM, Collins WE. *Plasmodium vivax* blood-stage dynamics. J Parasitol. 2008;88:521–535. 10.1645/0022-3395(2002)088[0521:PVBSD]2.0.CO;2 PMC2504326 12099421

[11] Barry AE, Waltmann A, Koepfli C, Barnadas C, Mueller I. Uncovering the transmission dynamics of *Plasmodium vivax* using population genetics. Pathog Glob Health. 2015;109:142–152. 10.1179/2047773215Y.0000000012 25891915 PMC4455355

[12] Liu Y, Auburn S, Cao J, Trimarsanto H, Zhou H, Gray K-A, et al Genetic diversity and population structure of *Plasmodium vivax* in central China. Malar J. 2014;13:262 10.1186/1475-2875-13-262 25008859 PMC4094906

[13] Gray K-A, Dowd S, Bain L, Bobogare A, Wini L, Shanks GD, et al Population genetics of *Plasmodium falciparum* and *Plasmodium vivax* and asymptomatic malaria in Temotu Province, Solomon Islands. Malar J. 2013;12:429 10.1186/1475-2875-12-429 24261646 PMC4222835

[14] Gunawardena S, Ferreira M, Kapilananda G, Wirth D, Karunaweera N. The Sri Lankan paradox: high genetic diversity in *Plasmodium vivax* populations despite decreasing levels of malaria transmission. Parasitology. 2014;141:880–890. 10.1017/S0031182013002278 24533989 PMC7485621

[15] Lin JT, Saunders DL, Meshnick SR. The role of submicroscopic parasitemia in malaria transmission: what is the evidence ? Trends Parasitol. 2014;30:183–190. 10.1016/j.pt.2014.02.004 24642035 PMC4049069

[16] malERA Consultative Group on Drugs. A research agenda for malaria eradication: drugs. PLoS Med. 2011;8:e1000402 10.1371/journal.pmed.1000402 21311580 PMC3026688

[17] World Health Organization. *Plasmodium vivax* control & elimination: development of global strategy and investment case. Geneva: World Health Organization; 2012.

[18] Lin E, Kiniboro B, Gray L, Dobbie S, Robinson L, Laumaea A, et al Differential patterns of infection and disease with *P*. *falciparum* and *P*. *vivax* in young Papua New Guinean children. PLoS ONE. 2010;5:e9047 10.1371/journal.pone.0009047 20140220 PMC2816213

[19] Senn N, Rarau P, Stanisic DI, Robinson L, Manong D, Salib M, et al Intermittent preventive treatment for malaria in Papua New Guinean infants exposed to *Plasmodium falciparum* and *P*. *vivax*: a randomized controlled trial. PLoS Med. 2012;9:e1001195 10.1371/journal.pmed.1001195 22479155 PMC3313928

[20] Michon P, Cole-Tobian JL, Dabod E, Schoepflin S, Igu J, Susapu M, et al The risk of malarial infections and disease in Papua New Guinean children. Am J Trop Med Hyg. 2007;76:997–1008. 17556601 PMC3740942

[21] Kasehagen LJ, Mueller I, McNamara DT, Bockarie MJ, Kiniboro B, Rare L, et al Changing patterns of *Plasmodium* blood-stage infections in the Wosera region of Papua New Guinea monitored by light microscopy and high throughput PCR diagnosis. Am J Trop Med Hyg. 2006;75:588–596. 17038678 PMC3728901

[22] Mueller I, Widmer S, Michel D, Maraga S, McNamara DT, Kiniboro B, et al High sensitivity detection of *Plasmodium* species reveals positive correlations between infections of different species, shifts in age distribution and reduced local variation in Papua New Guinea. Malar J. 2009;8:41 10.1186/1475-2875-8-41 19284594 PMC2657150

[23] Arnott A, Barnadas C, Senn N, Siba P, Mueller I, Reeder JC, et al High genetic diversity of *Plasmodium vivax* on the north coast of Papua New Guinea. Am J Trop Med Hyg. 2013;89:188–194. 10.4269/ajtmh.12-0774 23690553 PMC3748481

[24] Schultz L, Wapling J, Mueller I, Ntsuke PO, Senn N, Nale J, et al Multilocus haplotypes reveal variable levels of diversity and population structure of *Plasmodium falciparum* in Papua New Guinea, a region of intense perennial transmission. Malar J. 2010;9:336 10.1186/1475-2875-9-336 21092231 PMC3002378

[25] Koepfli C, Robinson LJ, Rarau P, Salib M, Sambale N, Wampfler R et al Blood-stage parasitaemia and age determine *Plasmodium falciparum* and *P*. *vivax* gametocytaemia in Papua New Guinea. PLoS ONE. 2015;10:e0126747 10.1371/journal.pone.0126747 25996916 PMC4440770

[26] Mueller I, Schoepflin S, Smith TA, Benton KL, Bretscher MT, Lin E, et al Force of infection is key to understanding the epidemiology of *Plasmodium falciparum* malaria in Papua New Guinean children. Proc Natl Acad Sci U S A. 2012;109:10030–10035. 10.1073/pnas.1200841109 22665809 PMC3382533

[27] Betuela I, Rosanas-Urgell A, Kiniboro B, Stanisic DI, Samol L, de Lazzari E, et al Relapses contribute significantly to the risk of *Plasmodium vivax* infection and disease in Papua New Guinean children 1–5 years of age. J Infect Dis. 2012;206:1771–1780. 10.1093/infdis/jis580 22966124

[28] Hetzel M, Pulford J, Gouda H, Hodge A, Siba P, Mueller I. The Papua New Guinea National Malaria Control Program: primary outcome and impact indicators, 2009–2014. Goroka: Papua New Guinea Institute of Medical Research; 2014.

[29] Mckenzie FE, Magill AJ, Forney JR, Lucas C, Erhart LM, Meara WPO, et al Gametocytemia in *Plasmodium vivax* and *Plasmodium falciparum* infections. J Parasitol. 2006;92:1281–1285. 17304807 10.1645/GE-911R.1 PMC2500222

[30] Douglas NM, Simpson JA, Phyo AP, Siswantoro H, Hasugian AR, Kenangalem E, et al Gametocyte dynamics and the role of drugs in reducing the transmission potential of *Plasmodium vivax* . J Infect Dis. 2013;208:801–812. 10.1093/infdis/jit261 23766527 PMC3733516

[31] Barbosa S, Gozze AB, Lima NF, Batista CL, Bastos MDS, Nicolete VC, et al Epidemiology of disappearing *Plasmodium vivax* malaria: a case study in rural Amazonia. PLoS Negl Trop Dis. 2014;8:e3109 10.1371/journal.pntd.0003109 25166263 PMC4148206

[32] Okell LC, Griffin JT, Kleinschmidt I, Hollingsworth TD, Churcher TS, White MJ, et al The potential contribution of mass treatment to the control of *Plasmodium falciparum* malaria. PLoS ONE. 2011;6:e20179 10.1371/journal.pone.0020179 21629651 PMC3101232

[33] Grueninger H, Hamed K. Transitioning from malaria control to elimination: the vital role of ACTs. Trends Parasitol. 2013;29:60–64. 10.1016/j.pt.2012.11.002 23228225

[34] Llanos-Cuentas A, Lacerda MV, Rueangweerayut R, Krudsood S, Gupta SK, Kochar SK, et al Tafenoquine plus chloroquine for the treatment and relapse prevention of *Plasmodium vivax* malaria (DETECTIVE): a multicentre, double-blind, randomised, phase 2b dose-selection study. Lancet. 2014;383:1049–1058. 10.1016/S0140-6736(13)62568-4 24360369

[35] Hanboonkunupakarn B, Ashley EA, Jittamala P, Tarning J, Pukrittayakamee S, Hanpithakpong W, et al Open-label crossover study of primaquine and dihydroartemisinin-piperaquine pharmacokinetics in healthy adult Thai subjects. Antimicrob Agents Chemother. 2014;58:7340–7346. 10.1128/AAC.03704-14 25267661 PMC4249579

[36] Genton B, D’Acremont VV, Rare L, Baea K, Reeder JC, Alpers MP, et al *Plasmodium vivax* and mixed infections are associated with severe malaria in children : a prospective cohort study from Papua New Guinea. PLoS Med. 2008;5:e127 10.1371/journal.pmed.0050127 18563961 PMC2429951

[37] Betuela I, Bassat Q, Kiniboro B, Robinson LJ, Rosanas-Urgell A, Stanisic D, et al Tolerability and safety of primaquine in Papua New Guinean children 1 to 10 years of age. Antimicrob Agents Chemother. 2012;56:2146–2149. 10.1128/aac.05566-11 22252800 PMC3318393

[38] Laman M, Laman M, Moore BR, Benjamin J, Padapu N, Tarongka N, et al Comparison of an assumed versus measured leucocyte count in parasite density calculations in Papua New Guinean children with uncomplicated malaria. Malar J. 2014;13:145 10.1186/1475-2875-13-145 24739250 PMC3991873

[39] Wampfler R, Mwingira F, Javati S, Robinson L, Betuela I, Siba P, et al Strategies for detection of *Plasmodium* species gametocytes. PLoS ONE. 2013;8:e76316 10.1371/journal.pone.0076316 24312682 PMC3848260

[40] Rosanas-Urgell A, Mueller D, Betuela I, Barnadas C, Iga J, Zimmerman PA, et al Comparison of diagnostic methods for the detection and quantification of the four sympatric *Plasmodium* species in field samples from Papua New Guinea. Malar J. 2010;9:361 10.1186/1475-2875-9-361 21156052 PMC3016373

[41] Koepfli C, Schoepflin S, Bretscher M, Lin E, Kiniboro B, Zimmerman PA, et al How much remains undetected? Probability of molecular detection of human *Plasmodia* in the field. PLoS ONE. 2011;6:e19010 10.1371/journal.pone.0019010 21552561 PMC3084249

[42] Koepfli C, Colborn KL, Kiniboro B, Lin E, Speed TP, Siba PM, et al A high force of *Plasmodium vivax* blood-stage infection drives the rapid acquisition of immunity in Papua New Guinean children. PLoS Negl Trop Dis. 2013;7:e2403 10.1371/journal.pntd.0002403 24040428 PMC3764149

[43] R Development Core Team. R: a language and environment for statistical computing. Vienna: R Found Statistical Computing; 2014.

[44] StataCorp. Stata release 12 [computer program]. College Station (Texas): StataCorp; 2011.

[45] Karunajeewa HA, Mueller I, Senn M, Lin E, Law I, Gomorrai PS, et al A trial of combination antimalarial therapies in children from Papua New Guinea. N Engl J Med. 2008;359:2545–2557. 10.1056/NEJMoa0804915 19064624

[46] Smith D, McKenzie F. Statics and dynamics of malaria infection in *Anopheles* mosquitoes. Malar J. 2004;3:13 15180900 10.1186/1475-2875-3-13 PMC449722

[47] Smith DL, Battle KE, Hay SI, Barker CM, Scott TW, McKenzie FE. Ross, Macdonald, and a theory for the dynamics and control of mosquito-transmitted pathogens. PLoS Pathog. 2012;8:e1002588 10.1371/journal.ppat.1002588 22496640 PMC3320609

[48] White MT, Karl S, Battle KE, Hay SI. Modelling the contribution of the hypnozoite reservoir to *Plasmodium vivax* transmission. Elife. 2014;3:1–19. 10.7554/eLife.04692 PMC4270097 25406065

[49] Robinson LJ, Wampfler R, Betuela I, Karl S, White MT, Li Wai Suen CSN, et al Data from: Strategies for understanding and reducing the *Plasmodium vivax* and *Plasmodium ovale* hypnozoite reservoir in Papua New Guinean children: A randomised placebo-controlled trial and mathematical model. Dryad Digital Repository. 10.1371/journal.pmed.1001891 PMC4624431 26505753

[50] Coleman R, Kumpitak C, Ponlawat A, Maneechai N, Phunkitchar V, Rachapaew N, et al Infectivity of asymptomatic *Plasmodium*-infected human populations to *Anopheles dirus* mosquitoes in western Thailand. J Med Entomol. 2004;41:201–208. 15061279 10.1603/0022-2585-41.2.201

[51] Sattabongkot J, Maneechai N, Rosenberg R. *Plasmodium vivax*: gametocyte infectivity of naturally infected Thai adults. Parasitology. 1991;102:27–31. 2038501 10.1017/s0031182000060303

[52] Bharti AR, Chuquiyauri R, Brouwer KC, Stancil J, Lin J, Llanos-cuentas A, et al Experimental infection of the neotropical malaria vector *Anopheles darlingi* by human patient-derived *Plasmodium vivax* in the Peruvian Amazon. Am J Trop Med Hyg. 2007;75:610–616. PMC1630632 17038681

[53] Imwong M, Boel ME, Pagornrat W, Pimanpanarak M, McGready R, Day NPJ, et al The first *Plasmodium vivax* relapses of life are usually genetically homologous. J Infect Dis. 2012;205:680–683. 10.1093/infdis/jir806 22194628 PMC3266132

[54] Chen N, Auliff A, Rieckmann K, Gatton M, Cheng Q. Relapses of *Plasmodium vivax* infection result from clonal hypnozoites activated at predetermined intervals. J Infect Dis. 2007;195:934–941. 10.1086/512242 17330782

[55] Bright AT, Manary MJ, Tewhey R, Arango EM, Wang T, Schork NJ, et al A high resolution case study of a patient with recurrent *Plasmodium vivax* infections shows that relapses were caused by meiotic siblings. PLoS Negl Trop Dis. 2014;8:e2882 10.1371/journal.pntd.0002882 24901334 PMC4046966

[56] Mueller I, Zimmerman PA, Reeder JC. *Plasmodium malariae* and *Plasmodium ovale*—the “bashful” malaria parasites. Trends Parasitol. 2007;23:278–283. 10.1016/j.pt.2007.04.009 17459775 PMC3728836

[57] White NJ. Malaria In: Cook GC, Zumla AI, editors. Manson’s tropical diseases. 21st ed. Philadelphia: W. B. Saunders; 2003 pp. 1205–1296.

[58] Siala E, Khalfaoui M, Bouratbine A, Hamdi S, Hili K, Aoun K. Relapse of *Plasmodium malariae* malaria 20 years after living in an endemic area. Presse Med. 2005;34:371–372. 15859572 10.1016/s0755-4982(05)83926-0

[59] Laman M, Moore BR, Benjamin JM, Yadi G, Bona C, Warrel J, et al Artemisinin-naphthoquine versus artemether-lumefantrine for uncomplicated malaria in Papua New Guinean children: an open-label randomized trial. PLoS Med. 2014;11:e1001773 10.1371/journal.pmed.1001773 25549086 PMC4280121

[60] Douglas NM, Nosten F, Ashley EA, Phaiphun L, van Vugt M, Singhasivanon P, et al *Plasmodium vivax* recurrence following falciparum and mixed species malaria: risk factors and effect of antimalarial kinetics. Clin Infect Dis. 2011;52:612–620. 10.1093/cid/ciq249 21292666 PMC3060895

[61] Karl S, Laman M, Koleala T, Ibam C, Kasian B, Drewei NN, et al Comparison of three methods for detection of gametocytes in Melanesian children treated for uncomplicated malaria. Malar J. 2014;13:1–8. 10.1186/1475-2875-13-319 25123055 PMC4139605

[62] Okell LC, Bousema T, Griffin JT, Ouédraogo AL, Ghani AC, Drakeley CJ. Factors determining the occurrence of submicroscopic malaria infections and their relevance for control. Nat Commun. 2012;3:1237 10.1038/ncomms2241 23212366 PMC3535331

[63] Schneider P, Bousema J, Gouagna L, Otieno S, van de Vegte-Bolmer M, Omar S, et al Submicroscopic *Plasmodium falciparum* gametocyte densities frequently result in mosquito infection. Am J Trop Med Hyg. 2007;76:470–474. 17360869

[64] Ouédraogo AL, Bousema T, Schneider P, de Vlas SJ, Ilboudo-Sanogo E, Cuzin-Ouattara N, et al Substantial contribution of submicroscopical *Plasmodium falciparum* gametocyte carriage to the infectious reservoir in an area of seasonal transmission. PLoS ONE. 2009;4:e8410 10.1371/journal.pone.0008410 20027314 PMC2793432

[65] Koch R. Professor Koch’s investigations on malaria: fourth report to the Colonial Department of the German Colonial Office. Br Med J. 1900;1:1597–1598. 20759083 PMC2506827

[66] Griffin JT, Hollingsworth TD, Okell LC, Churcher TS, White M, Hinsley W, et al Reducing *Plasmodium falciparum* malaria transmission in Africa: a model-based evaluation of intervention strategies. PLoS Med. 2010;7:e1000324 10.1371/journal.pmed.1000324 20711482 PMC2919425

[67] Crowell V, Briët OJT, Hardy D, Chitnis N, Maire N, Di Pasquale A, et al Modelling the cost-effectiveness of mass screening and treatment for reducing *Plasmodium falciparum* malaria burden. Malar J. 2013;12:4 10.1186/1475-2875-12-4 23286228 PMC3544609

[68] Tiono AB, Ouédraogo A, Ogutu B, Diarra A, Coulibaly S, Gansané A, et al A controlled, parallel, cluster-randomized trial of community-wide screening and treatment of asymptomatic carriers of *Plasmodium falciparum* in Burkina Faso. Malar J. 2013;12:79 10.1186/1475-2875-12-79 23442748 PMC3599538

[69] Tiono AB, Ouédraogo A, Diarra A, Coulibaly S, Soulama I, Konaté AT, et al Lessons learned from the use of HRP-2 based rapid diagnostic test in community-wide screening and treatment of asymptomatic carriers of *Plasmodium falciparum* in Burkina Faso. Malar J. 2014;13:30 10.1186/1475-2875-13-30 24467946 PMC3925413

[70] Halliday KE, Okello G, Turner EL, Njagi K, Mcharo C, Kengo J, et al Impact of intermittent screening and treatment for malaria among school children in Kenya: a cluster randomised trial. PLoS Med. 2014;11:e1001594 10.1371/journal.pmed.1001594 24492859 PMC3904819

[71] Mosha JF, Sturrock HJW, Greenhouse B, Greenwood B, Sutherland CJ, Gadalla N, et al Epidemiology of subpatent *Plasmodium falciparum* infection: implications for detection of hotspots with imperfect diagnostics. Malar J. 2013;12:221 10.1186/1475-2875-12-221 23815811 PMC3701503

[72] Baird JK, Hoffman SL. Primaquine therapy for malaria. Clin Infect Dis. 2004;39:1336–1345. 10.1086/424663 15494911

[73] Beutler E. G6PD deficiency. Blood. 1994;84:3613–3636. 7949118

[74] Kondrashin A, Baranova AM, Ashley EA, Recht J, White NJ, Sergiev VP. Mass primaquine treatment to eliminate vivax malaria: lessons from the past. Malar J. 2014;13:51 10.1186/1475-2875-13-51 24502194 PMC3931915

[75] Von Seidlein L, Auburn S, Espino F, Shanks D, Cheng Q, McCarthy J, et al Review of key knowledge gaps in glucose-6-phosphate dehydrogenase deficiency detection with regard to the safe clinical deployment of 8-aminoquinoline treatment regimens: a workshop report. Malar J. 2013;12:112 10.1186/1475-2875-12-112 23537118 PMC3616837

[76] Kim S, Nguon C, Guillard B, Duong S, Chy S, Sum S, et al Performance of the CareStartTM G6PD deficiency screening test, a point-of-care diagnostic for primaquine therapy screening. PLoS ONE. 2011;6:e28357 10.1371/journal.pone.0028357 22164279 PMC3229584

[77] Battle KE, Karhunen MS, Bhatt S, Gething PW, Howes RE, Golding N, et al Geographical variation in *Plasmodium vivax* relapse. Malar J. 2014;13:144 10.1186/1475-2875-13-144 24731298 PMC4021508

