# Supplementary figures and images for: Strategies for Understanding and Reducing the Plasmodium vivax and Plasmodium ovale Hypnozoite Reservoir in Papua New Guinean Children: A Randomised Placebo-Controlled Trial and Mathematical Model
Source: PLoS Med. 2015 Oct 27;12(10):e1001891. doi: 10.1371/journal.pmed.1001891 (PMC4624431; doi:10.1371/journal.pmed.1001891)

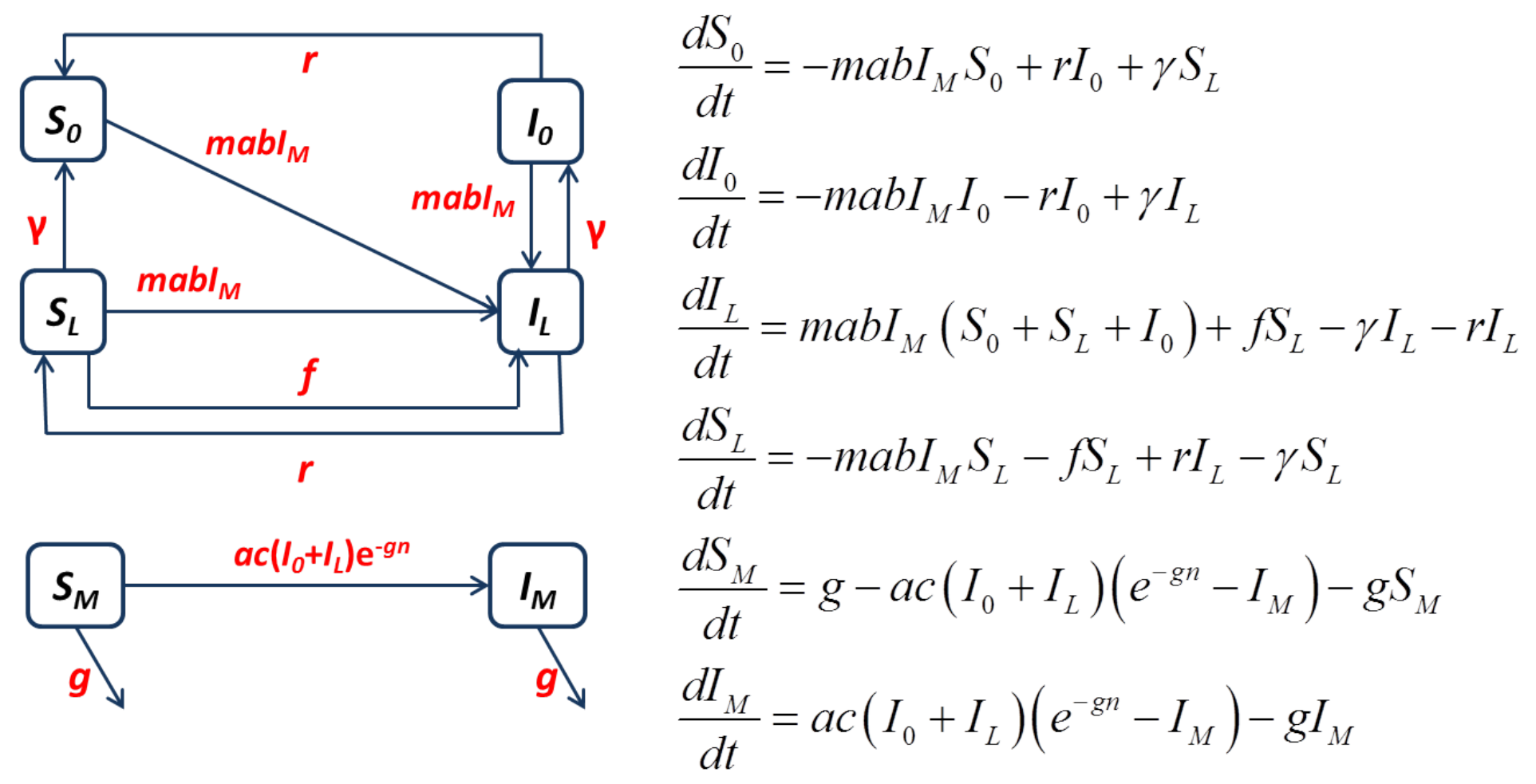

Supplement: S1 Fig — S 0 denotes fully susceptible humans, I 0 denotes individuals with blood-stage infection, S L denotes individuals with liver-stage infection with hypnozoites, and I L denotes individuals with blood-stage infection and liver-stage infection with hypnozoites. (TIF) [file pmed.1001891.s001.tif]

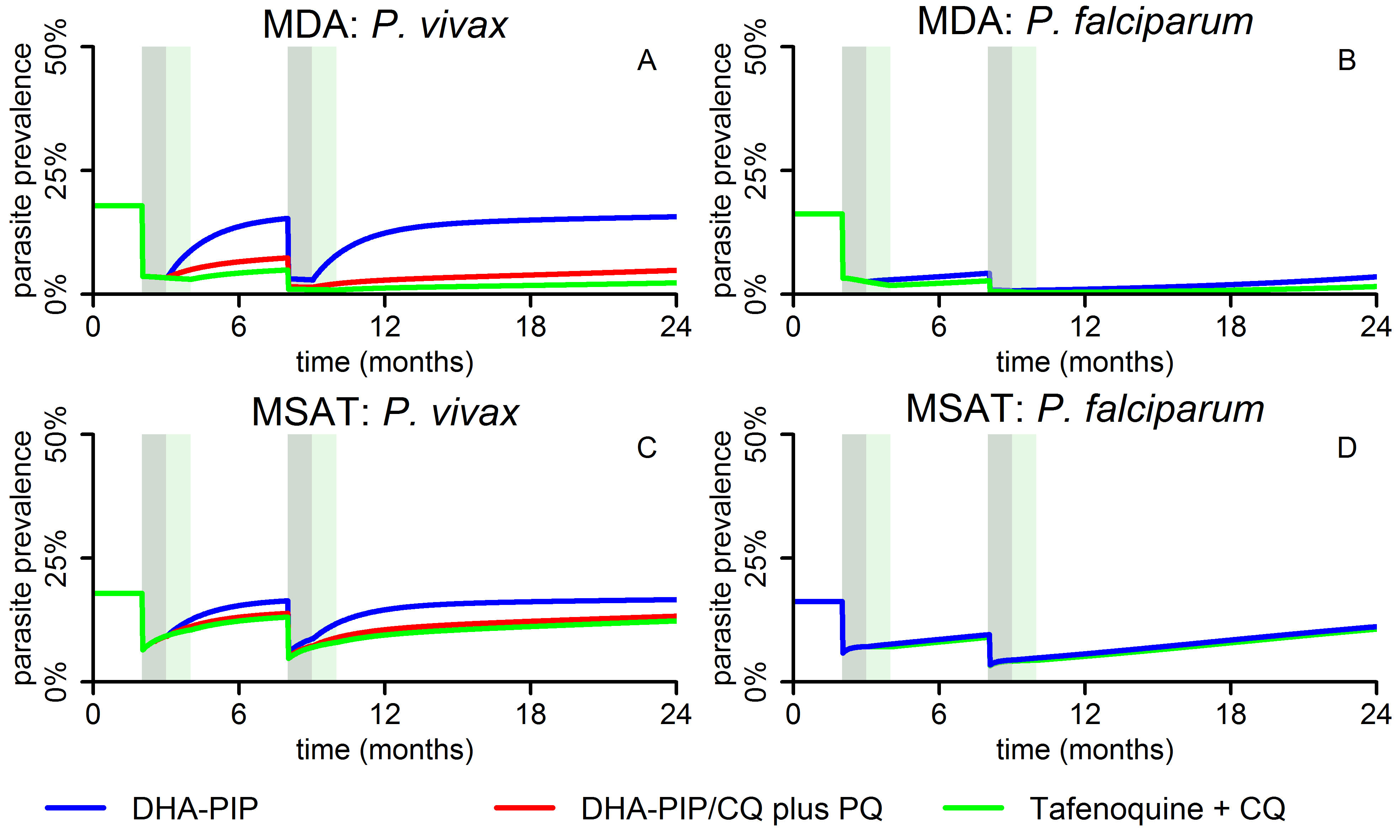

Supplement: S2 Fig — The grey and green shaded bars denote the duration of prophylactic protection for DHA-PIP/CQ and tafenoquine, respectively, after each treatment round. DHA-PIP and CQ were assumed to be administered as part of a 3-d regimen providing prophylaxis for 1 mo. PQ was assumed to be administered as part of a 14-d regimen providing prophylaxis for 15 d. Tafenoquine was assumed to be administered via a single dose providing prophylaxis for 2 mo. (TIF) [file pmed.1001891.s002.tif]
