# Supplementary material for: Strategies for Understanding and Reducing the Plasmodium vivax and Plasmodium ovale Hypnozoite Reservoir in Papua New Guinean Children: A Randomised Placebo-Controlled Trial and Mathematical Model
Source: PLoS Med. 2015 Oct 27;12(10):e1001891. doi: 10.1371/journal.pmed.1001891 (PMC4624431; doi:10.1371/journal.pmed.1001891)
Supplement: S1 Table — (PDF) [file pmed.1001891.s003.pdf]

S1 Table: Description of parameters for malaria transmission model.

Parameter	Description	Value	Reference
Mosquitoes			
a	mosquito biting frequency ($a = Q/\delta$)	0.21 day^{-1}	calculated
Q	human blood index (*An. farauti*)	0.64	[1]
δ	length of gonotrophic cycle	3 days	[2]
n	duration of sporogony in mosquito	12 days	[3]
g	mosquito death rate ⇔ 1/mosquito life expectancy	0.1 day^{-1}	[3]
c	transmission probability: human to mosquito (*An. darlingi)*	0.23	[4]
m	number of mosquitoes per human (*P. vivax*)	0.56 (0.511)[a]	calculated
m	number of mosquitoes per human (*P. falciparum)*	1.45 (1.362) [a]	calculated
Humans			
b	transmission probability: mosquito to human	0.5	[5]
r	rate of clearance of blood-stage infections	1/60 day^{-1}	[6]
f	relapse frequency (~ time to first relapse)	1/125 day^{-1}	[7]
γ	rate of hypnozoite clearance	1/500 day^{-1}	[8]
Treatment			
	coverage (5% pregnant women, 15% missed or refused)	80%	[d]
	diagnostic sensitivity (molecular PCR)	80%	[e]
	diagnostic specificity	95%	[e]
	duration of prophylactic protection (DHA-PIP/chloroquine)[b]	30 days	[9]
	duration of causal prophylactic protection (14 day PQ regimen)[c]	15 days	
	duration of prophylactic protection (tafenoquine)[c]	60 days	[10]
	primaquine effectiveness (5% G6PD deficient, 20% failure including 5% CYP 2D6 low metaboliser)	75%	[10]
	tafenoquine effectiveness (5% G6PD deficient, 5% failure including CYP 2D6 low metaboliser)	90%	[10]

[a]Estimated numbers of mosquitoes per human predicted to give an equilibrium parasite prevalence of 20% in the deterministic (stochastic) model. [b]Prophylactic prevention of new blood-stage infections. [c]Prophylactic prevention of new blood-stage and liver-stage infections. [d] Intervention coverage was set at 80%, based on the assumption that pregnant women, who can not be treated with primaquine, represent 5% of the population and that 15% of the population are either missed or refused to participate. This level of participation is comparable to that achieved in recent trials of mass drug administration for the elimination of yaws [11]. [e] The choice of 80% diagnostic sensitivity reflects both the limited detectability of *Plasmodium spp.* by standard PCR (~90% [12]) and the presence of infections with ultra-low parasites

densities that are below the limit of detection of standard PCR [13]. Although PCR-based diagnosis is highly specific, diagnostic specificity was assumed to be 95% to account for either non-specific amplifications and/or human errors.

References

1. Garrett-Jones C. The human blood index of malaria vectors in relation to epidemiological assessment. Bull World Heal Organ. 1964;30: 241–61.

2. Killeen GF, Mckenzie FE, Foy BD, Schieffelin C, Billingsley F, Beier JC. A simplified model for predicting malaria entomologic inoculation rates based on entomologic and parasitologic parameters relevant to control. Am J Trop Med Hyg. 2000;62: 535–544.

3. Gething PW, Van Boeckel TP, Smith DL, Guerra C a, Patil AP, Snow RW, et al. Modelling the global constraints of temperature on transmission of Plasmodium falciparum and *P. vivax.* Parasit Vectors. BioMed Central Ltd; 2011;4: 92. doi:10.1186/1756-3305-4-92

4. Bharti AR, Chuquiyauri R, Brouwer KC, Stancil J, Lin J, Llanos-cuentas A, et al. Experimental infection of the neotropical malaria vector *Anopheles darlingi* by human patient-derived *Plasmodium vivax* in the Peruvian Amazon. Am J Trop Med Hyg. 2007;75: 610–616.

5. Smith DL, Drakeley CJ, Chiyaka C, Hay SI. A quantitative analysis of transmission efficiency versus intensity for malaria. Nat Commun. 2010;1: 108. doi:10.1038/ncomms1107

6. Collins WE, Jeffery GM, Roberts JM. A retrospective examination of anemia during infection of humans with *Plasmodium vivax.* Am J Trop Med Hyg. 2003;68: 410–2. Available: http://www.ncbi.nlm.nih.gov/pubmed/12875288

7. Battle KE, Karhunen MS, Bhatt S, Gething PW, Howes RE, Golding N, et al. Geographical variation in *Plasmodium vivax* relapse. Malar J. Malaria Journal; 2014;13: 1–16. doi:10.1186/1475-2875-13-144

8. Malato Y, Naqvi S, Schürmann N, Ng R, Wang B, Zape J, et al. Fate tracing of mature hepatocytes in mouse liver homeostasis and regeneration. J Clin Invest. 2011;121: 4850–60. doi:10.1172/JCI59261DS1

9. Bergstrand M, Nosten F, Lwin K, Karlsson M, White N, Tarning J. Characterization of an in vivo concentration-effect relationship for piperaquine in malaria chemoprevention. Sci Transl Med. 6: 260ra147.

10. Llanos-Cuentas A, Lacerda M V, Rueangweerayut R, Krudsood S, Gupta SK, Kochar SK, et al. Tafenoquine plus chloroquine for the treatment and relapse prevention of *Plasmodium vivax* malaria (DETECTIVE): a multicentre, double-blind, randomised, phase 2b dose-selection study. Lancet. 2014;383: 1049–58. doi:10.1016/S0140-6736(13)62568-4

11. Mitjà O, Houinei W, Moses P, Kapa A, Paru R, Hays R, et al. Mass Treatment with Single-Dose Azithromycin for Yaws. N Engl J Med. 2015;372: 703–710. doi:10.1056/NEJMoa1408586

12. Koepfli C, Schoepflin S, Bretscher M, Lin E, Kiniboro B, Zimmerman P a, et al. How much remains undetected? Probability of molecular detection of human *Plasmodia* in the field. PLoS One. 2011/05/10 ed. 2011;6: e19010. doi:10.1371/journal.pone.0019010

13. Hofmann N, Mwingira F, Shekalaghe S, Robinson LJ. Ultra-Sensitive Detection of *Plasmodium falciparum* by Amplification of Multi-Copy Subtelomeric Targets. PLoS Med. 2015;12: 1–21. doi:10.1371/journal.pmed.1001788
