# Supplementary material for: Strategies for Understanding and Reducing the Plasmodium vivax and Plasmodium ovale Hypnozoite Reservoir in Papua New Guinean Children: A Randomised Placebo-Controlled Trial and Mathematical Model
Source: PLoS Med. 2015 Oct 27;12(10):e1001891. doi: 10.1371/journal.pmed.1001891 (PMC4624431; doi:10.1371/journal.pmed.1001891)
Supplement: S1 Text — (PDF) [file pmed.1001891.s004.pdf]

Protocol for

THE EFFECT OF LIVER AND BLOOD-STAGE TREATMENT ON SUBSEQUENT *PLASMODIUM* RE-INFECTION AND MORBIDITY

Principal Investigators: Inoni Betuela, Louis Schofield, Hernando del Portillo and Ivo Mueller

Protocol Authors: Inoni Betuela, Leanne Robinson and Ivo Mueller

PNG Institute of Medical Research, Papua New Guinea

Version Number:
Version 1.1

Day/Month/Year

11th January 2011

Statement of Compliance

The study will be carried out in accordance with Good Clinical Practice (GCP) as required by the following:

- ICH GCP E6
- Completion of Human Subjects Protection Training

SIGNATURE PAGE

The signature below documents the approval of this protocol and the attachments, and provides the necessary assurances that this trial will be conducted according to all stipulations of the protocol, including all statements regarding confidentiality and according to local legal and regulatory requirements and to the principles outlined in applicable United States federal regulations and ICH guidelines.

Principal Investigator – Ivo Mueller, Papua New Guinea Institute of Medical Research*:*

Signed: 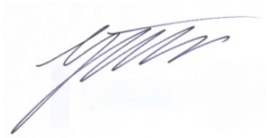 Date: 11.01.10

Principal Investigator – Inoni Betuela, Papua New Guinea Institute of Medical Research*:*

Signed: 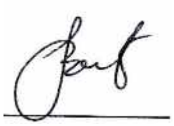 Date: 11.01.10

Table of Contents

Statement of Compliance ii
Signature Page iii
Protocol Summary ix

1 Key Roles 1
2 Background Information and Scientific Rationale 4
2.1 Background Information 4
2.2 Rationale 5
2.3 Potential Risks and Benefits 6
2.3.1 Potential Risks 6
2.3.2 Known Potential Benefits 7
3 AIMS & Objectives 9
4 Study Design 12
5 Study Population 14
5.1 Selection of the Study Population 14
5.2 Inclusion/Exclusion Criteria 15
6 Study Procedures/Evaluations 17
6.1 Study Procedures 17
6.1.1 Field Study Team 17
6.1.2 Enrollment and Pre-screening 17
6.1.3 Randomisation 18
6.1.4 Drug Treatment 18
6.1.5 Baseline measurements and sample collection 19
6.1.5.1 Participant Database and Study Visit Activities 19
6.1.6 Active Surveillance 20
6.1.7 Passive case detection 21
6.1.8 Assessment of Participant Health Status 22
6.1.8.1 Blood Smear Confirmation of RDT-based Diagnosis 23
6.1.8.2 Mobile IMR Clinics 23
6.1.8.3 Diagnosis and Treatment 23
6.1.9 Health Center Surveillance Records 24
6.1.9.1 Health Record Booklets 24
6.2 Laboratory Evaluations 24
6.2.1 Laboratory Evaluations/Assays 24
6.2.1.1 Diagnosis of Malaria Infection by Blood Smear 24
6.2.1.2 Sample Separation and DNA Extraction 25
6.2.1.3 Diagnosis of Malaria Infection by Polymerase Chain Reaction/Ligase Detection Reaction 25
6.2.1.4 Host Genetic Typing 25
6.2.1.5 Immunological and molecular evaluations 26

Table of Contents - *continued*

6.2.2 Specimen Collection, Preparation, Handling and Shipping 26
6.2.2.1 Instructions for Specimen Preparation, Handling, and Storage 26
6.2.2.2 Specimen Shipment 27
7 Study Schedule 28
7.1 Community Information 28
7.2 Enrolment/Screening & Randomisation 29
7.3 Drug treatment & Baseline Visit 29
7.4 Follow-up and Final Visits 30
7.4.1 Follow-up Visits 30
7.5 Early Termination Visit 30
7.6 Criteria for Discontinuation or Withdrawal of a Subject 30
8 Assessment of Outcome Measures 31
8.1 Specification of the Appropriate Outcome Measures 31
8.1.1 Primary Outcome Measures 31
8.1.2 Secondary Outcome Measures 31
9 Safety assessment and reporting 32
9.1 Definition of Adverse Event (AE) 32
9.2 Definition of Serious Adverse Event 32
9.3 Reporting Procedures 32
9.3.1 Serious Adverse Event Detection and Reporting 33
9.3.2 Type and Duration of the Follow-up of Subjects after Adverse Events 33
9.4 Halting Rules 33
10 Clinical Monitoring Structure 34
11 Statistical Considerations 35
11.1 Study Outcome Measures 35
11.1.1 Definitions of Malaria Infections 35
11.1.2 Primary Outcome Measures 35
11.1.2.1 Incidence of clinical *P. falciparum* episodes during the two 32 weeks of follow-up 35
11.1.2.2 Incidence of first or only *P. vivax* infection 36
11.1.2.3 Time to first *P. vivax* infection 36
11.1.3 Secondary Outcome Measures 36
11.2 Sample Size Considerations 36
11.2.1 Primary Outcome Measures 36
11.2.2 Secondary Outcome Measures 37
11.3 Analysis Plan 37
11.3.1 Primary Outcome Measures 37
11.3.1.1 Incidence of clinical *P. falciparum* episodes during follow-up 37
11.3.1.2 Incidence of first or only *P. vivax* infection 38
11.3.1.3 Time-to-first *P. vivax* infection 38

Table of Contents - *continued*

11.3.2 Secundary Outcome Measures ... 38
12 Access to Source Data/Documents ... 39
13 Quality Control and Quality Assurance ... 40
14 Ethics/Protection of Human Subjects ... 41
14.1 Declaration of Helsinki ... 41
14.2 Institutional Review Board ... 41
14.3 Informed Consent Process ... 41
14.4 Subject Confidentiality ... 43
14.5 Future Use of Stored Specimens ... 43
15 Data Handling and Record Keeping ... 45
15.1 Data Management Responsibilities ... 45
15.2 Data Capture Methods ... 45
15.3 Timing/Reports ... 46
15.4 Study Records Retention ... 46
15.5 Protocol Deviations ... 46
16 Publication Policy ... 48
17 Literature References ... 49
18 SUPPLEMENTS/APPENDICES ... 51

Protocol Summary

Title: Host and parasites factors contributing to risk of *Plasmodium* re-infection and morbidity in elementary school children in Maprik, East Sepik Province

Population: 500 children 5-10 years of age, who are residents of malaria-endemic villages near Maprik in the East Sepik Province, Papua New Guinea (PNG).

Number of Sites: 1 field site (4-8 elementary schools, Maprik District, PNG), 4 laboratory sites (Goroka & Madang PNG, Melbourne, Barcelona)

Study Duration: 9 months

Subject Duration: 9 months

Aims:

Aim 1: Compare the effect of liver and blood-stage pre-treatment with blood-stage pre-treatment only on the incidence of malarial infections and disease in children 5-10 years of age.

Aim 2: Measure naturally acquired immune responses to both *P. falciparum* and *P. vivax* and investigate their association with subsequent risk of *P. falciparum* and *P. vivax* infection and disease in children 5-10 years of age.

Objectives:

Primary Objectives:

- Determine the contribution of long-lasting liver-stages to risk of infection with *P. vivax* (Aim 1).
- Describe the dynamics of acquisition and loss of *P. falciparum* and *P. vivax* infections in semi-immune children (Aim 1)
- determine the extent of naturally acquired humoral and cellular immune responses to *P. falciparum* and *P. vivax* in children aged 5-10 years (Aim 2).
- determine the association of immune responses with protection against *P. falciparum* and *P.vivax* infection and disease in children aged 5-10 years (Aim 2).

Secondary Objectives:

- Determine the contribution of long-lasting liver-stages to risk of infection with *P. ovale* (Aim 1).
- Determine the contribution of long-lasting liver-stages to risk of clinical disease with *P. vivax* and *P. ovale* in semi-immune children (Aim 1).
- Investigate the relationship between presence of asexual and sexual stage *P. vivax* and *P. falciparum* parasites following radical cure. (Aim 1)
- Assess the effect of cumulative prior exposure to malaria on risk of acquiring new malarial infection and risk of developing clinical illness (Aim 1 & 2)
- evaluate the effect of clearing liver and blood-stage infections vs clearing blood-stage infections only on cellular and humoral immune responses. (Aim 2)
- compare cellular and humoral immune responses to *P. falciparum* and *P.vivax* in children aged 5-10 years from this region of PNG with those from children aged 5-14 years in the Mugil region of PNG. (Aim 2)

Schematic of Study Design: A study schedule diagram is provided in Appendix 1.

1 KEY ROLES

Individuals:

Principal Investigators:

Ivo Mueller, PhD,
PNGIMR,
P.O. Box 60,
Goroka, EHP 441
Papua New Guinea,
T: +675 732 2800 ext 231,
F: +675 732 1998,
e: ivomueller@fastmail.fm

Inoni Betula, MBBS, MMed,
PNGIMR,
P.O. Box 60,
Goroka, EHP 441
Papua New Guinea,
T: +675 732 2800 ext 231,
F: +675 732 1998,
e: Inoni.Betuela@pngimr.org.pr

Peter Siba, PhD,
PNGIMR,
P.O. Box 60,
Goroka, EHP 441
Papua New Guinea,
T: +675 732 2800
F: +675 732 1998,
e: Peter.Siba@pngimr.org.pg

Louis Schofield, PhD,
Walter & Eliza Hall Institute of Medical Research,
1G Royal Parade,
Parkville, VIC 3050,
Australia,
T: +613-9345-2474,
F: +613-9347-0852,
e: schofield@wehi.edu.au

Hernando del Portillo, PhD
Centre de Recerca en Salut Internacional de Barcelona (CRESIB)
Rosselló, 132, 4th floor
08036 Barcelona.
Spain
T: +34 932 275 706
F: +34 932 279 853
e: hernandoa.delportillo@cresib.cat

Co-Investigators

Leanne Robinson, PhD,
PNGIMR,
P.O. Box 378,
Madang, MAD 511
Papua New Guinea,
T: +675 852 2909
F: +675 852 3289,
e: robinson@wehi.edu.au

Anna Rosanas
PNGIMR,
P.O. Box 378,
Madang, MAD 511
Papua New Guinea,
T: +675 852 2909
F: +675 852 3289,
e: anna.rosanas@cresib.cat

Institutions:

PNG Institute of Medical Research (PNGIMR),
PO Box 60, Goroka
Papua New Guinea,
T: +675-732-2800 ext 231,

F: +675-732-1998,
e: ivomueller@fastmaill.fm.

Walter & Eliza Hall Institute of Medical Research,
1G Royal Parade,
Parkville, VIC 3050,
Australia,
T: +613-9345-2474,
F: +613-9347-0852,
e: schofield@wehi.edu.au

Centre de Recerca en Salut Internacional de Barcelona (CRESIB)
Rosselló, 132, 4th floor
08036 Barcelona.
Spain
T: +34 932 275 706
F: +34 932 279 853
e: hernandoa.delportillo@cresib.cat

Protocol Statisticians:

Ivo Mueller, PhD
PNGIMR
PO Box 60
Goroka, EHP 441
Papua New Guinea
T: +675 732 2800 ext.231
F: +675 732 1998
e: pngimr_ivo@datec.net.pg

Leanne Robinson, PhD,
PNGIMR,
P.O. Box 378,
Madang, MAD 511
Papua New Guinea,
T: +675 852 2909
F: +675 852 3289,
e: robinson@wehi.edu.au

Protocol Data Managers:

Thomas Adiguma, BSc,
PNG Institute of Medical Research,
P.O. Box 378,
Madang, MAD 511,
Papua New Guinea,
T: +675-852-2909,
F: +675-852-3289,
e: Thomas.Adiguma@pngimr.org.pg

Protocol Epidemiologist:

Ivo Mueller, PhD
PNGIMR
PO Box 60
Goroka, EHP 441
Papua New Guinea
T: +675 732 2800 ext.231
F: +675 732 1998
e: pngimr_ivo@datec.net.pg

Protocol Clinician:

Inoni Betula, MBBS, MMed,
PNGIMR,
P.O. Box 60,
Goroka, EHP 441
Papua New Guinea,
T: +675 732 2800 ext 231,
F: +675 732 1998,
e: Inoni.Betuela@pngimr.org.pr

Community Advisory Board Liaison:

Lawrence Rare
Field Operations Manager,
PNG Institute of Medical Research,
P.O. Box 400, Maprik, ESK 511,
Papua New Guinea
T: +675-858-1294,,
F: +675-858 1257,
e: lrare@pngimr.org.pg

Collaborating Investigators:

James Beeson, MBBS PhD,
Dept. of Infections & Immunity, Walter & Eliza Hall Institute of Medical Research,
1 G Royal Parade,
Parkville, VIC 3050,
Australia,
T: +61 3 9345 2555

F: +61 3 9347 0852,
e: beeson@wehi.edu.au

Peter Zimmerman, PhD
A. Prof of Medicine,
Center for Global Health and Diseases,
Case Western Reserve University,
CASE School of Medicine,
10900 Euclid Avenue,
Wolstein Research Building, 4th Floor,
Cleveland, Ohio 44106-7286,
T: 216-368-0508,
F: 216-368-4825,
e: peter.zimmerman@case.edu

Pedro Alonso, MD PhD
Centre de Recerca en Salut Internacional
de Barcelona (CRESIB)
Rosselló, 132, 4th floor
08036 Barcelona.
Spain
T: +34 932 275 706
F: +34 932 279 853
e: alonso@clinic.ub.es

Ingid Felger, PhD
Swiss Tropical Institute
Socinstrasse,
P.O. Box,
4002 Basel,
Switzerland
T: +41-61-284 81 11,
F: +41-61-284 81 01,
e: Ingrid.Felger@unibas.ch

2 BACKGROUND INFORMATION AND SCIENTIFIC RATIONALE

2.1 Background Information

Although the majority of malaria related mortality globally is attributed to *P. falciparum*, episodes of *P. vivax* account for over 50% of clinical malaria cases outside of Africa (1). Although it is often thought to be a relatively mild, non-life threatening disease, there is increasing recognition that *P. vivax* contributes to significant disease. In particular as there are increasing reports of severe and even fatal cases of *P. vivax* from Turkey (2), India (3), Brazil (Lacerda et al, pers comm.) and the Island of New Guinea(4-6). Due to this heightened awareness of the burden of *P. vivax* on affected populations, studies investigating the epidemiology of *P. falciparum* and *P. vivax* in regions of co-infection are again being seen as a health priority. As *P. vivax* is more easily transmissible than *P. falciparum* (1), and can relapse from long lasting liver-stages, control of *P. vivax* through vector or case management presents unique challenges. This can be seen in Thailand (7, 8) or Brazil(9) where after years of relatively efficient vector control and case management, *P. vivax* is now a more common cause of illness than *P. falciparum*. As such, studies specifically assessing the impact of pre-erythrocytic and blood-stage clearance on subsequent rates of infection and morbidity in children of different ages are urgently required in areas of high *P. vivax* transmission.

The PNGIMR recently conducted a longitudinal cohort study in children 1-3 years of age in a population near Maprik, East Sepik Province that is co-endemic for all 4 human *Plasmodium* species (MRAC 05.19). This study showed that *P. vivax* is the most prevalent malaria species in this region and contributes equally with *P. falciparum* to the burden of malarial disease in children < 3 years of age. In order to provide a baseline for the testing of both pre-erythrocytic and anti-bloodstage vaccines, the PNGIMR is in the process of completing a second study in the same cohort of children (aged 1-5 years) near Maprik. In that study children were randomised to either receive pre-treatment with artesunate and primaquine or a placebo. In addition, to these two vaccine baseline study arms a 3rd group of children received a blood stage drug (artesunate) but not a liver stage drug (primaquine) thus allowing the contribution of long lasting liver stages to the burden of infection and morbidity with *P. vivax* to be assessed. This study will not only provide the necessary baseline information for both future *P. vivax* pre-erythrocytic and blood stage vaccine trials but also allow us to determine correlates of immunity to *P. vivax* in children aged 1-5 years and to investigate the contribution of relapses to the burden of *P. vivax* in young children in an area of high *P. vivax* transmission.

However, the high level of morbidity observed in both cohorts resulted in participating children having to be treated with antimalarials very regularly during follow-up. This has made it difficult to study both the both contribution of liver-stages to burden of infection and the dynamic acquisition

and loss of malarial infections in these cohorts. In addition, the relatively low state of immunity in these young children limit investigation on immune correlates to those against clinical disease but impair our ability to determine correlates of immunity against both pre-erythrocytic and blood stage infections with either *P. falciparum* or *P. vivax*.

We therefore propose to conduct a further cohort study in slightly older children, aged 5-10 years, that will allow getting more detailed information regarding the importance of (long-lasting) liver-stages as well as naturally pre-erythrocytic and anti-blood stage immunity on subsequent rates of *Plasmodium* infection and disease in children aged 5-10 years. Such information will also be crucial for the informed design and testing of both pre-erythrocytic and erythrocytic vaccines and provide further insights correlates of (clinical) immunity to *P. falciparum* and *P. vivax i*n children aged 5-10 years in a region of high *P. falciparum* and *P. vivax* transmission. Last but not least, the study will provide an opportunity to study *P. falciparum* and *P. vivax* strain dynamics without interference of frequent antimalarial treatments.

A previous study in children aged 6-12 years, conducted on the North Coast of Madang Province, showed that by 6 years of age children have acquired almost complete clinical immunity to *P. vivax* but still experience a high burden of *P. falciparum* illness, indicating that immunity to *P. vivax* is acquired significantly faster than immunity to *P. falciparum* despite similar levels of transmission (10). In this study, acquisition of immunity was characterized by increasing control of high parasite densities via both humeral and cellular immune responses (11, 12). It will be important to confirm these observations in the proposed study and investigate possible reasons for the differing speed of immune acquisition and attempt to identify both humoral and cellular correlates of immunity to both *P. falciparum* and *P. vivax*.

The proposed study will randomize children to either receive pre-treatment with chloroquine/primaquine/Coartem or chloroquine/placebo/Coartem, followed by eight months of intensive follow-up. This will therefore allow us to determine the impact of radical pre- erythrocytic / erythrocytic clearance on the subsequent burden of infection and morbidity with *P. falciparum* and *P. vivax.* Thereby, this unique study design will thus not only allow us to determine the contribution of liver-stages to the burden of malarial infection and disease but also provides an opportunity to investigate how parasite diversity and pre-erythrocytic and anti-blood stage immunity combine to influence the risk of malaria in semi-immune children

2.2 Rationale

This study specifically seeks to obtain important epidemiological data on the effect of radical pre-erythrocytic and erythrocytic clearance on subsequent rates of *Plasmodium* infection and disease in children aged 5-10 years using a liver-stage/blood-stage treatment to re-infection study design. In addition to this epidemiological data, the study will assess the natural acquisition of cellular and

humoral immune responses to *P. falciparum* and *P. vivax*, thus assisting in the determination of correlates of clinical immunity to *P. falciparum* and *P. vivax* in PNG children aged 5-10 years. These data will not only be essential for development of future against *P. vivax* and *P falciparum* but provide invaluable insight into the contribution of long-lasting liver-stages to the force of infection with *P. vivax* that will contribute towards designing more rational approaches to the treatment of *P. vivax* both in the context of case management and future attempts at elimination.

2.3 Potential Risks and Benefits

2.3.1 Potential Risks

This study uses two types of anti-malarial drugs to remove blood and liver stages of malarial infections in the study children. A 3 day course of Coartem (arthemeter-lumefantrine (AL)) will be used to clear blood stages in both pre-treatment arms while primaquine (PQ) will be used to eradicate liver stages in the pre-erythrocytic pre-treatment arm. In addition, a 3-day course of chloroquine will be administered to all study children at the beginning of drug treatment to assist with the clearance of blood-stages and achieve effective clearance of hypnozoites, together with primaquine, in the pre-erythrocytic arm. AL and chloroquine are well tolerated in PNG children and the schedule and dosage used in the trial correspond to the national standard treatment guidelines. Primaquine has been extensively used in the past in PNG as a gametocidal drug as well as in a short course for eradication of *P. vivax* liver stages but is rarely used today. Primaquine is generally well tolerated and has been used for the eradication of *P. vivax* liver stages in children as young as 3 months in trials of mass drug administration for the eradiation of malaria in Vanuatu (13). In addition, in the recent cohort conducted in Ilaita 150 children 1-5 years were treated with 14 days of primaquine. Detailed monitoring of side effects revealed that drug was safe and very well tolerated (Betuela et al in prep). As the drug may lead to gastro-intestinal side effects (nausea etc) if not taken with food, we will give the drug with a bit of soft food prior to drug administration and actively monitor side effects during the entire duration of treatment. The use of primaquine is contraindicated in people with G-6-PD deficiency where treatment with 8-aminoquinolines may induce hemolytic anaemia. We will therefore screen all eligible study children for the presence of G-6-PD deficiency and children with less than 60% G-6-PD activity will be excluded from the study. Children with mild G-6-PD deficiency (60-80% activity) will be randomized to the two arms of the study.

Although the study objectives require frequent blood sampling, the volumes of blood collected do not represent a health risk to a normal child aged 5-10 years. Children with severe anaemia and/or severe malnutrition will however be excluded from participating in the study. Although remote, there is a small, finite risk of infection associated with finger-stick and venous blood draws. However, the Papua New Guinea Institute of Medical Research (PNGIMR) has conducted many studies involving routine finger-stick and venous blood sampling from children 5 to 10 years of age in neighboring

populations and based on our experience form these studies, very few if any study-related adverse experiences, as defined below, have occurred or been reported to us.

Venipunctures will be performed on veins in the participants' arms. Upon removal of the needle, localized bleeding is efficiently stopped with a small pressure bandage (ie, a band-aid). Participants may experience some pain as a result of the needle insertion. If the needle punctures the blood vessel, there is a risk of local bleeding that can result in minor hematoma. In order to assure the safety of participants, they will be monitored following blood draw until it can be ascertained the local bleeding has stopped. Parents and/or guardians will be instructed to take children to the study nurse based at Albinama health center in case of localized hematomas or infection at the site of needle injection who will then attend to them and if necessary refer them for further treatment. Venous blood draws will only be done by the study clinician or (pediatric) nurses with special training in drawing venous blood from small children.

As part of the study, sick children diagnosed by the study team will be referred to an IMR-run field clinic or the local health centre for treatment. Children will be treated with Coartem according to new national treatment guidelines.

The participation in this study is thus not associated with any long-term risks for study participants.

2.3.2 Known Potential Benefits

The present study has potential direct benefits for the study participants and general benefits for populations in malaria endemic areas in the Southwest Pacific, Southeast Asia and South America.

For participants a potential benefit is the intensive clinical follow-up resulting in earlier diagnosis and treatment of malaria and other common childhood illnesses. In addition, children with G-6-PD deficient will be diagnosed and then counseled in regards to what drug or food the children should avoid.

For PNG and the wider scientific community the study will provide an in-depth assessment of the burden of malarial infection and disease in childhood in PNG and thereby assist in improving diagnosis and treatment. In particular, the determination of the contribution of long lasting liver stages to the burden of *P. vivax* infections and episodes will assist in re-assessing the potential use of liver stage eradication in the national antimalarial treatment guidelines and in future attempts at local malarial elimination. The close monitoring of the PQ treatment will provide important safety and efficacy data on the use of Primaquine for liver stage eradication in children 5-10 years, while the G-6-PD testing will provide data on the prevalence and severity of G-6-PD deficiency in lowland PNG.

Last but not least, the study will contribute to the development of malaria vaccines and interventions by providing an in-depth assessment of naturally acquired immunity to *P. falciparum* and *P. vivax*. The knowledge thus gained will assist in accelerating field testing of existing vaccine candidates.

3 AIMS & OBJECTIVES

Aim 1: Compare the effect of liver and blood-stage pre-treatment with blood-stage pre-treatment only on the incidence of malarial infections and disease in children 5-10 years of age.

This study aims to determine how pre-treatment with a short acting blood stage or a combination of short-acting blood stage and liver-stage drug influences the risk of subsequent malarial infections and disease in children 5-10 years of age. A 9 month longitudinal cohort study with children randomized to receive Coartem (3d), chloroquine (3d) plus either primaquine (20d) or placebo will be carried out.

Primary Objectives:

- Determine the contribution of long-lasting liver-stages to risk of infection with *P. vivax*.
- Describe the dynamics of acquisition and loss of *P. falciparum* and *P. vivax* infections in semi-immune children

Secondary Objectives:

- Determine the contribution of long-lasting liver-stages to risk of infection with *P. ovale*.
- Determine the contribution of long-lasting liver-stages to risk of clinical disease with *P. vivax* and *P. ovale* in semi-immune children.
- Investigate the relationship between presence of asexual and sexual stage *P. vivax* and *P. falciparum* parasites following radical cure.
- Assess the effect of cumulative prior exposure to malaria on risk of acquiring new malarial infection and risk of developing clinical illness

Assessment of Aims and Objectives:

The ***first primary objective*** will be addressed by determining the time-to-first *P. vivax and P. ovale* infection as well as the incidence of first and only infection with *P. vivax* and *P. ovale* in children that have received pre-treatment with either, blood-stage drugs alone (chloroquine/Coartem plus placebo), or blood-stage plus liver-stage drugs (chloroquine/Coartem plus primaquine) through fortnightly active case detection. By comparing the incidence in both groups it will be possible to determine the contribution of long-lasting liver-stages to the burden of infections under conditions of high transmission intensity.

The ***second primary objective*** will be addressed by genotyping all *P. vivax* and *P. falciparum* infection observed during the two 6-monthly follow-up periods and calculating rates of acquisition and loss as well as average duration of individual infections using methods that allow for imperfect detection and by fitting an immigration-death model to the full sequences of observations similar to the methods used by Falk et al (14). By comparing the dynamics to both *P. falciparum* and *P. vivax* as well as between the two study arms, it will be possible to determine difference between the two most common human malaria parasites in their within-host dynamics and assess the contribution of liver-stage to the dynamics of *P. vivax* infections.

The secondary objectives will be assessed by:

- determining the rates of clinical episodes of *P. falciparum* in children that have received pre-treatment with either, blood-stage drugs alone (chloroquine/coartem plus placebo), or blood-stage plus liver-stage drugs (chloroquine/coartem plus primaquine) through two 6-month follow-up periods.
- Determining the presence of gametocytes using light microscopy and reverse-transcriptase PCR from filter paper blood spot and comparing the prevalence of both asexual and sexual parasites in all blood samples collected.
- studying the associations between increased age and incidence of malarial infections and disease in both treatment arms.

Aim 2: Measure naturally acquired immune responses to both *P. falciparum* and *P.vivax* and investigate their association with subsequent risk of *P. falciparum* and *P.vivax* infection and disease in children 5-10 years of age.

This study aims to ascertain the extent of naturally acquired cellular and humoral immune responses to a range of *P. falciparum* and *P. vivax* blood pre-erythrocyte stage antigens in children 5-10 years age and determine their possible associations with protection against *P. falciparum* and *P.vivax* infections and disease. For that purpose we will measure immune responses in venous samples collected from all children after drug treatment and relate different immune measurements to risk of infection and disease during the 8 months of follow-up.

Primary objectives:

- determine the extent of naturally acquired humoral and cellular immune responses to *P. falciparum* and *P.vivax* in children aged 5-10 years.
- determine the association of immune responses with protection against *P. falciparum* and *P.vivax* infection and disease in children aged 5-10 years.

Secondary objectives:

- evaluate the effect of clearing liver and blood-stage infections vs clearing blood-stage infections only on cellular and humoral immune responses.
- compare cellular and humoral immune responses to *P. falciparum* and *P.vivax* in children aged 5-10 years from this region of PNG with those from children aged 5-14 years in the Mugil region of PNG.

Assessment of Aims and Objectives:

The ***first primary objective*** will be assessed by examining cellular and humoral immune responses using plasma and PBMC from a 10 mL venous bleed collected at the cessation of drug treatment.

The ***second primary objective*** will involve relating immune responses measured at the cessation of drug treatment with the incidence of *P. falciparum* and *P.vivax* infections and disease in the subsequent 8 months as determined under Aim 1.

The secondary objectives will be assessed as follows:

- PBMC and serological assays will be performed on samples acquired after the cessation of the drug treatment regimes to evaluate the impact of clearing liver and blood-stage infections vs clearing blood-stage infections only, on cellular and humoral immune responses.
- PBMC and serological assays performed on baseline samples of the Mugil treatment-re-infection study will be applied to the above mentioned samples in this study to compared cellular and humoral responses in the two cohorts and confirm previous associations between immune responses and protection.

4 STUDY DESIGN

In this study 500-600 children aged 5-10 years will be screened, enrolled and randomized to 2 arms of a longitudinal cohort study. The randomized arms will comprise:

- 250-300 children pre-treated with 3 days Chloroquine (CQ), 4 weeks Primaquine (PQ) and 3 days Coartem (CO)
- 250-300 children pre-treated with 3 days Chloroquine (CQ), 4 weeks Placebo (PL) and 3 days Coartem (CO)

At enrolment all eligible children aged 5-10 years in the study area will be screened for Glucose-6-Phosphate Dehydrogenase deficiency (G-6-PD) and the presence of severe anaemia or malnutrition. Children with less than 60% G-6-PD activity, haemoglobin <5g/dl or severe malnutrition (weight-for-age nutritional Z score [WAZ] <60th percentile) will be excluded from the study. All other children will be randomized to the two treatment arms of the study.

Drug treatment will commence at enrolment. All children will receive CQ on days 1-3. Children will also receive either PQ (0.5 mg/kg) or placebo on days 1-5, 8-12, 15-19 and 22-26 (i.e. Monday to Friday for 4 weeks). During the third week of drug treatment all children will receive CO on days 15-17 (according to national treatment guidelines). Children will be actively monitored for side effects including measurement of haemoglobin levels at days 1, 8, 15 and 22.

Following completion of treatment, children will undergo a clinical examination and a 10 mL venous blood sample will be collected on day 29 (i.e. 26 days after their last dose of CQ, 12 days after their last dose of CO and 3 days after their last dose of PQ or PL.

Subsequently, all enrolled children will be followed using 2-weekly active and continuous passive case detection at a local health centre for 8 months (Week 2-32). Active follow-up consists of morbidity surveillance visits to monitor clinical malaria illness not reported to a health facility, as well as blood sampling for the detection of asymptomatic malaria. These visits will include collection of a medical history of the preceding 2 weeks to determine incidence of malarial infections and bed net usage, a physical examination, measurement of axillary temperature. A 250µl finger prick blood sample will be collected very 2 weeks for the first 12 weeks and 4 weekly thereafter, i.e. weeks 2, 4, 6, 8, 10, 12, 16, 20, 24, 28 and 32.

All blood samples will be assesed for the presence, type, and number of malarial infections (by light microscopy and PCR) and Hb levels. The 10 mL venous blood samples will also be used for the assessment of humoral and cellular immune responses to *Plasmodium* infections (Aim 2). DNA

extracted from venous blood samples and the finger-prick blood samples collected during follow-up will be assessed in molecular parasitology studies (Aim 1).

During the entire study period, a passive case detection system will be maintained at the local Health Centre. Every participating child that presents at the health centre with a febrile illness will be clinically assessed and have a finger prick blood sample taken for determination of malaria infections by a Rapid Diagnostic Test (RDT), two blood slides for microscopy confirmation and HemoCue® for Hb concentration. In accordance with the new national treatment guidelines, only children with a positive RDT will receive Coartem treatment from the attending field Nurse. All the data collected will be recorded in the morbidity forms and the health book of each participating child to be reviewed by the study clinician.

Over the 9-month study duration, each participant will have 38 contacts with the study team (1 for enrolment/screening, 20 for supervision of drug administration, 1 baseline time-points (after drug treatment), 16 active follow-up visits) and will be asked to contribute one 10 mL venous blood sample at the baseline visit and 12 finger prick blood samples (250µL) (see Study Schedule Diagram, Appendix I.) Additional finger prick blood samples may be collected at the time of febrile illness during morbidity follow-ups and passive case detection.

5 STUDY POPULATION

5.1 Selection of the Study Population

Data from both active and passive morbidity surveillance in the Wosera area show that the incidence of both *P falciparum* and *P vivax* malaria is greatest in the 1- to 5-year old age group but remains a significant burden in the 5-10 year old age bracket, during which time the children are acquiring clinical immunity, particularly to episodes of *P. falciparum*.. A recent study in Madang (10) showed that in an area of hyperendemic transmission children of the 6-12 year age group had already acquired a high degree of clinical immunity to *P. vivax* but were still experiencing significant numbers of *P. falciparum* clinical episodes despite similar incidence of new blood stage infections.

This study will thus be done in children 5-10 years of age (±3months). This age group is selected because:

a) Children in this age group have acquired a sufficient degree of immunity to exert a significant amount of control on parasite but are still at significant risk of symptomatic illness with at least *P. falciparum*.
b) An earlier study in a similar age group (5-12) in Madang showed that this age group is thus ideal for determination of immunological correlates of natural acquisition of immunity to *P. falciparum.* A 2nd study in the same age group is now required will allow findings from a similar study north of Madang to be confirmed in a children in a different region of PNG.
c) This will allow for an extension of a previous liver-stage/blood-stage treatment to re-infection study into slightly older children. Together these studies will cover the entire age range (1-10 yrs) from non-immune to highly clinically immune.
d) The lower number of clinical episodes in this age group will allow to study the dynamics of acquisition and loss of individual *P. falciparum* and *P. vivax* infections in semi-immune individual in the absences of frequent drug treatment.

The study will enroll 500-600 children with approximately the same numbers of male and females. All study participants will be ethnic Melanesian.

Children will be recruited from elementary schools in the vicinity of the Albimana health center (see Figure 3 in Section 5.2). Albimana is an ideal study location. It has a similar malaria prevalence rate as the neighboring Ilaita area and is accessible by road all year round.

Before commencing the project, the study team will hold community information meetings and conduct a baseline census in all potential study villages and thereby obtain a list of families who have eligible children. These families will then be contacted and invited to take part in the study.

See Section 6.0 Informed Consent Process for details on how families will be contacted.

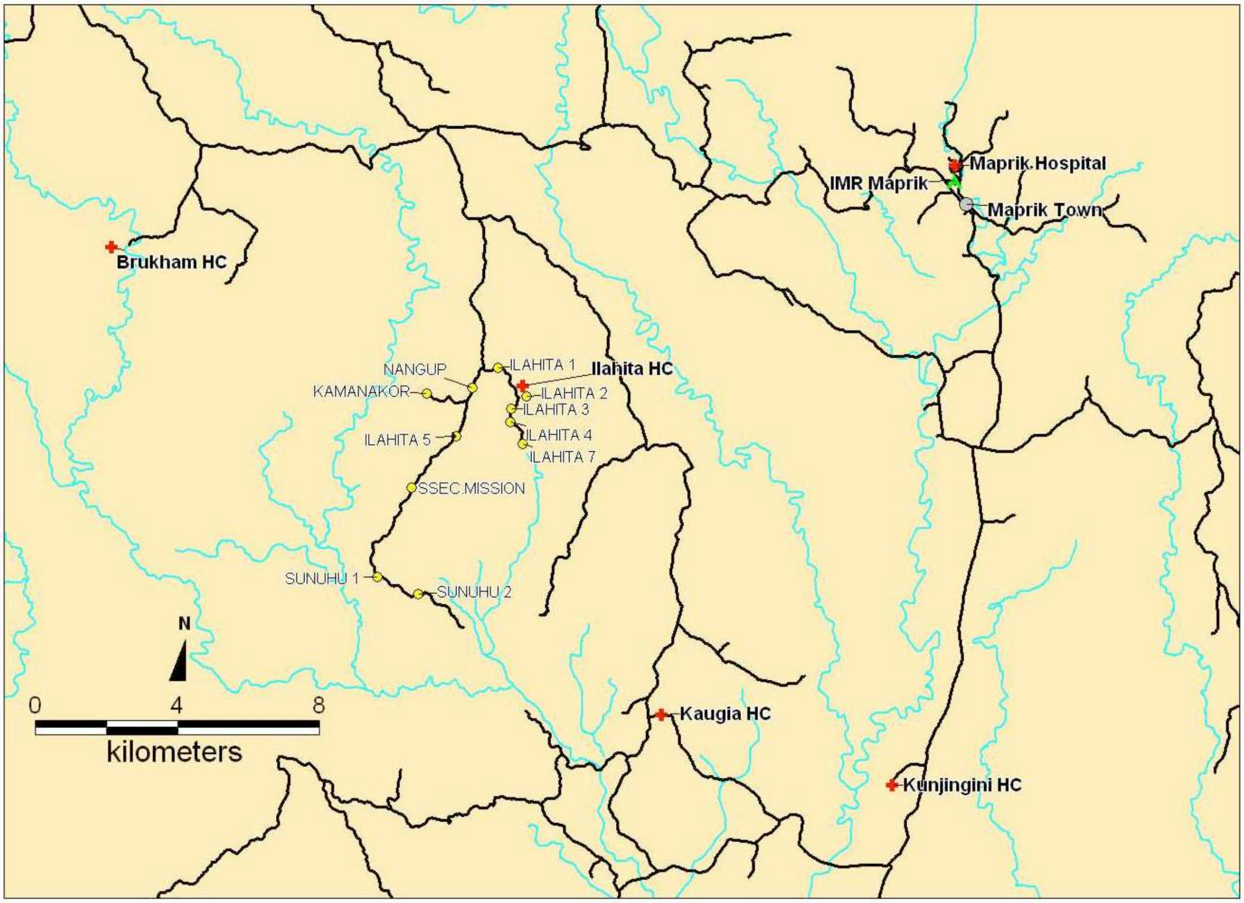

Figure 3. Study location – The Ilahita/Albinama/Wosera area, East Sepik Province, Papua New Guinea

5.2 Inclusion/Exclusion Criteria

Children are eligible for participation in this study if they fulfill the following criteria:

- aged 5-10 years (±3 months in children without known date of birth),

- permanent residents of the area,
- absence of history of hypersensitivity reactions to the pre-treatment drugs.

At enrollment and upon informed consent, all eligible participants (see Section 5.1 above for criteria) will be screened for the following exclusion criteria:

- chronic illness,
- severe malnutrition (weight-for-age nutritional Z score [WAZ] <60th percentile)
- severe anemia (Hb <5 g/dL),
- G-6-PD deficiency (<60% G-6-PD activity)
- permanent disability, which prevents or impedes study participation.

Any 1 or more of the criteria is sufficient to exclude study participation.

Children with moderate to severe anemia (i.e. Hb 5 - 8 g/dL) can be enrolled, but will be treated according to national guidelines with a full course of antimalarials, albendazole and 4 weeks of daily oral iron supplementation. Children with severe malnutrition and severe anaemia (Hb < 5.0g/dlL will be referred to the local health centre for treatment according to national treatment guidelines.

6 STUDY PROCEDURES/EVALUATIONS

6.1 Study Procedures

An overview of the study procedures is provided below and is discussed in greater detail in the following subsections.

6.1.1 Field Study Team

The study will be coordinated and day-to-day activities will be carried out by 2 PNGIMR field study teams with qualified registered nurses. All study staff are trained specifically to conduct informed consent, recruit participants, and organize and conduct the collection of blood and other measurements based on the research collaborators' study protocols. The study coordinator has received appropriate human subjects protections and study conduct training to conduct the study according to Good Clinical Practice (GCP) guidelines. The team will be supervised by a study coordinator and Specialist Physician on and off site. All clinical staff have the necessary registration with PNG nursing and medical boards and are thus allowed to dispense drug treatments.

6.1.2 Enrollment and Screening

Community awareness will be carried out through community leaders, village elders, health authorities, and village Reporters in the study area. An Information session for the parents of participating children will then be organized in the villages in the form of meetings to brief them on the broad aims and objectives of the study. After these meetings, a census of eligible children in the study area will be carried out before a formal informed consent is obtained (see section 6.0) from the parents to enroll their children in the study.

Following these consultations prospective participants will be screened for their suitability for inclusion in the study. After obtaining informed consent from parents or legal guardians, children will be screened for eligibility and their demographic (age, sex) and anthropometric data (weight) recorded and Hb levels determined. In order to prevent treating children with G-6-PD deficiency with primaquine, all children will be tested for G-6-PD deficiency at time of enrollment. All eligible children will be assigned a unique PNGIMR identification number. This number will be used to identify that child for the duration of the study.

Children with reported symptomatic illness will be examined by the study team and treated as required. In children with presumptive malaria illness the illness will be diagnosed on the spot using a rapid malarial diagnostic test (RDT). Children with a positive RDT test and/or and Hb < 8 g/dL will be treated with a full course of Coartem in addition to the albendazole and oral iron supplementation.

6.1.3 Randomisation

Only children with normal G-6-PD status (>60% G-6-PD activity) will be randomised.to the 2 study arms, i.e. CQ/PQ/Coartem, CQ/PL/Coartem. Every child's health (record) booklet will be clearly marked with a study identifier and study identification number. For children without or with a badly damaged health book, new health books will be issued at no cost to the participant. At any study contact, the study team will record all medically relevant data collected during their examination of the child.

6.1.4 Drug Treatment

Drug treatment will at enrolment. All children will receive a course of course of Directly Observed Treatment (DOT) with CQ on days 1-3. Depending on the treatment arm children have been randomized to, they will also receive DOT of either PQ (0.5 mg/kg) or placebo(PL) on days 1-5, 8-12, 15-19 and 22-26 (i.e. Monday to Friday for 4 weeks). The use of placebo (PL) allows both the participants and study teams to be blinded to the treatments arms. During the third week of drug treatment all children will receive Coartem on days 15-17. All drug treatments will be given with food and observed by the study team. If children do not swallow the drugs properly or vomit them within 30 minutes of administration another dose will be administered.

In order for all children to complete treatment at the same time, the following schedule will be implemented for both rounds of drug treatment:

Week 1: Commence CQ/PQ and CQ/PL treatment regime

Week 2: Continue PQ and PL treatment regime

Week 3: Commence Coartem/PQ and Coartem/PL treatment regime

Week 4: Continue PQ and PL treatment regime

Treatment side effects will be monitored by study staff at DOT treatment time points. Hemoglobin will be measured at regular intervals during the treatment period to assure that no children are undergoing a hemolytic reaction to primaquine. Children that show adverse reactions to chloroquine, primaquine or Coartem will be excluded from further pre-treatment and subsequent participation in the study. Parents and/or guardians of study participants will be encouraged to seek

medical care for their children, to report any symptoms of malaria, fever, or illness at the earliest opportunity, and to report any anti-malarial treatments received or used. Parents and/or guardians of study participants will be encouraged to seek medical attention immediately if their child has any sign or symptoms of illness or any other condition that they deem as a threat to their child's well-being. They will be advised of their rights to withdraw from the study at anytime should they wish to do so and no medical care or treatment will be refused by the PNGIMR field staff as a form of penalty.

6.1.5 Baseline measurements and sample collection

Baseline measurements and venous blood collection (10 mL) will be conducted 4 weeks after enrollment and 3 days after the final dose of the PQ and PL drug regime. During the baseline visits the PNGIMR field study team will conduct the following tasks:

- Assess the child's health status including
  - body temperature
  - symptoms of malaria or other infections
  - spleen size
- Interview the parent and/or guardian and record:
  - history of child's bed net use in 2 weeks prior,
  - child's history of febrile illness/malaria in 2 weeks prior,
  - child's history of malaria treatment in 2 weeks prior (except treatments given by study),
- Check the child's health records booklet for recent health center visits and record applicable information,
- Collect 10 mL venous blood sample,
  - Prepare thin and thick blood smears,
  - Prepare filter paper blood spot
  - Archive samples for laboratory analyses (PCR-typing plus auxiliary immunology),

If the participant has symptoms of clinical malaria he/she will be diagnosed in the field using a rapid diagnostic test (RDT) for malaria and HemoCue® for Hb concentration. For children excluded from undertaking RDT tests, blood smears will be read overnight for malaria diagnosis. Upon positive RDT, or positive blood smear or in the case of moderate-to-severe anemia (Hb <8.0 g/dL) the study team will initiate treatment with Coartem according to national treatment quidelines.

6.1.5.1 Participant Database and Study Visit Activities

After enrollment, the child's baseline information will be entered into a participant's database that will provide scheduling information for the surveillance activities every 2 weeks. The participants'

database will be updated every 2 weeks to reflect the status of the cohort through indicators that distinguish children under active follow-up versus those censored from follow-up for various reasons. The participant database will serve to schedule active surveillance visits for the study children. Using the baseline fixed information on the demographics of the participant and the dynamic information on the follow-up status of the child, active surveillance forms will be pre-printed from the participants database with study identification numbers, family information, and measurements and information to be collected during the study visit.

6.1.6 Active Surveillance

Following the collection of the 10 mL venous blood sample the study team will contact each participating child every 2 weeks and conduct a brief clinical evaluation of each child during a clinic held at the participant's school.

The study team will specifically perform the following tasks:

- Record axillary body temperature of the child,
- Assess the child for symptoms of malaria infection,
- Determine if the child is suffering from symptomatic malaria (or clinical signs and symptoms of anemia),
- Check the fingers of the child for redness or signs of irritation from lancet finger punctures.
- Record information relating to:
    - history of bed net use for the study participant since the previous visit,
    - participant's history of illness since previous visit,
    - history of malaria treatment for the study participant since previous visit,
- Check the child's health records booklet for recent health center visits and record applicable information,
- Inform the child's teacher and school administration of the next follow-up visit and the reasons for the tasks to be performed.

If the participant is judged to be suffering from malaria illness at the time of the morbidity surveillance visit, finger prick blood samples will be collected for determination of parasitaemia and Hb levels. The presence of malarial parasites will be diagnosed in the field using RDT. For children excluded from undertaking RDT tests, blood smears will be read overnight for malaria diagnosis. Upon positive RDT or positive blood smear or in the case of moderate-to-severe anemia (Hb <8.0 g/dL) the study team will initiate treatment as per national standard treatment guidelines.

In the first 12 weeks of follow-up, a 250 µL finger prick blood sample will be collected at 2 weekly morbidity surveillance visits. Thereafter a finger prick sample will be collected every 4 weeks only.

The blood samples will be used to produce blood smears for assessing blood-stage malaria infection, type of *Plasmodium* parasite(s), and level of parasitaemia. A portion of this sample will be collected in a microtainer for further analysis (i.e., PCR/LDR-based diagnosis of malaria infection and for immunological studies). A few drops of blood will be collected on a filter paper for detection of gametocytes.

In total the team will conduct 16 follow-up visits that will include 11 finger prick blood sampling time points.

All enrollment, baseline and active follow-up visits will be done by 2 mobile teams in the study communities. A 3rd team will help during the intensive DOT pre-treatment phase of the study. Each school will be visited on the same days over a 2-week period. Symptomatic children detected during the study clinics will be referred to the Albinama health centre or Maprik Hospital.

6.1.7 Passive case detection

The schools in the study are within less than 10km (i.e. up to 3 hours walk) of Albinama health centre. The health centre has both in-patient facilities and is staffed with a health extension officer and several nurses and community health workers. The health centre staff routinely assesses patients for malaria and upper respiratory tract infections (the 2 most common reasons for seeking treatment). As at all other PNG health centres, attendance and admittance log books are kept.

The study team will coordinate with the health centre nursing staff for the collection of illness and treatment histories of study participants on special Health Centre Surveillance Forms. The health centre staff will be requested to screen incoming patients who are between 5-10 years of age in order to determine if they are study participants. If the health centre staff identifies the incoming patient as a study participant, the nursing staff will refer the patient to a health centre based study nurse. The nurse will take detailed history, fill in the Health Centre Surveillance form, do a finger prick blood sample to obtain blood smears and for determination of infecting malaria species, measure Hb and confirm the presumptive diagnosis using RDT, unless the child has received malaria treatment in the previous 2 week period. For children excluded from undertaking RDT tests, blood smears will be read overnight for malaria diagnosis. In the case of a positive RDT or positive blood smear or, a Hb <8.0g/dL, the child will be treated with a locally available appropriate antimalarial treatment and iron supplementation by the study nurse.

Results of RDT and Hb measurement will be passed onto health centre staff. For children with negative RDT the health centre staff will be advised to refine the diagnosis for other febrile illnesses and treat accordingly.

All health centre visits will be recorded in the child's health booklet, which will be cross-checked at the active morbidity surveillance time points.

6.1.8 Assessment of Participant Health Status

Standard anthropometric measurements, body temperature, and Hb concentration (in children with RDT confirmed malarial infections and/or clinical signs of anaemia) will be taken as discussed and will be recorded on a preprinted CRF (the Study Team Visit Form) at the time of measurement. At the time of the study visit, the Nursing Officer will review the child's growth measurements, body temperature, and reported symptoms (if any). The Nursing Officer will consider the following in his evaluation of the child's health status:

Table 2. Checklist for Assessment of Participant Health Status

**Measurement or Health Indicator**		
Growth Measurements	Weight for age	Below 60th percentile
Axillary Temperature		≥37.5°C
**Symptoms of Illness**		
Fingers (previous blood draw puncture sites)	Redness, irritation, pus, swelling	
Present Clinical Symptoms of Illness	Chills<br>Sweating<br>Cough<br>Diarrhea<br>Convulsions	
Reported Symptoms of Illness	Fever<br>Chills<br>Sweating<br>Headache<br>Earache<br>Cough<br>Diarrhea<br>Abdominal Pain<br>Convulsions	

The parents and/or guardians of the participant will be informed if their child's growth measurements and temperature are indicative of a health risk. If at the time of the study visit, the child is ill, has any of the signs or symptoms listed above, has measurements indicating that the child may be malnourished or is at risk of malnutrition, or may require clinical attention, the Nursing Officer will initiate treatment locally or recommend transfer to Albinama health center or Maprik Hospital. At the end of Study Team Visits, the field study team will provide transportation for ill study participants to the Albinama health center at no cost.

6.1.8.1 Blood Smear Confirmation of RDT-based Diagnosis

Blood smears from all symptomatic children made during the study visits by the field study team will be read overnight by the Microscopist at the PNGIMR's Maprik field station to confirm RDT diagnosis. Should smear results show the presence of malarial parasites in children with negative RDT results, the study team will search out the children and initiate treatment with Coartem.

Blood slides from asymptomatic children will be read retrospectively.

Results from all smears will be entered onto the study's microscopy results form and subsequently entered into the study database.

6.1.8.2 Mobile IMR Clinics

Consistent with its function as the research arm of the PNG Ministry of Health, the PNGIMR is fully licensed to dispense treatment to sick people in its areas of operation, irrespective of their participation in research studies.

If parents and/or guardians of study participants prefer for their child to be treated by the local health center rather than the mobile IMR clinic, the study will assist with transport and cover outpatient consultation fees (upon presentation of voucher only).

6.1.8.3 Diagnosis and Treatment

All participants diagnosed with presumptive malaria by health center staff will be referred to the health centre based IMR nurse. The IMR nurse will take detailed history, fill in the Health Center Surveillance form, do a finger-stick blood sample to obtain blood smears, measure Hb and confirm the presumptive diagnosis using a RDT, unless the child has received malaria treatment in the previous 2 week period. . For children excluded from undertaking RDT tests, blood smears will be read overnight for malaria diagnosis. In the case of a positive RDT or positive blood smear or, an Hb <8.0 g/dL the child will be treated with a locally appropriate antimalarial treatment and iron supplementation by the IMR nurse.

Results of RDT and Hb measurement will be passed on to health center staff. For children with negative RDT the health center staff will be advised to refine the diagnosis for other febrile illnesses and treat accordingly.

The PNG Institute of Medical Research will be fully responsible for maintaining the presence of a fully register nursing practitioner at Albinama Health Centre and for all treatments dispensed through the IMR nurse.

6.1.9 Health Center Surveillance Records

The PNGIMR field study team will coordinate with the health center for the collection and, if necessary, extraction of specific health information on study participants. In order to assist the health center with the passive morbidity surveillance, a study nurse will be based at the health center during all out patient clinic hours. In addition, rudimentary morbidity surveillance will be conducted if participants attend aid posts or community health clinics surrounding the Albinama health centre. Data from the Health Center and Aid Post Surveillance Forms also are entered into a Health Center Surveillance Database at regular intervals and maintained by the PNGIMR.

6.1.9.1 Health Record Booklets

All participants in PNGIMR studies are recommended to purchase and carry a health records booklet when seeking medical care at health centers. The health records booklet provides information as to the immunizations, illnesses, and treatments an individual has had. Additionally, the health records booklets for participants in collaborative research studies are marked with a brightly colored sticker. The sticker alerts health care providers at the health centers that the individual is participating in a research study.

Each of the health records booklets of study participants will be marked with a sticker denoting participation in this study. In the event a participant of the study does not have a health records booklet, a booklet will be provided at no cost to the parents and/or guardians of the participant and marked with a unique sticker showing study participation. Ilahita health center and surrounding Aid Post staff will be informed of the study and the type of sticker denoting participation.

The field study team will review each participant's health records booklet at the regularly scheduled study visits, record all health care visits on the Study Team Visit Form, and coordinate with the health center at least every 2 weeks to ensure that extracts of the Health Center Surveillance Forms have been obtained.

6.2 Laboratory Evaluations

6.2.1 Laboratory Evaluations/Assays

6.2.1.1 Diagnosis of Malaria Infection by Blood Smear

Two thick and two thin smears will be prepared on each participant in the study at every follow-up visit (or on an as needed basis) by PNGIMR staff who have extensive experience in field preparation of blood smears. Evaluation of the blood smear is performed by examining a total number of oil immersion fields to include 200 leukocytes, with the assumption that mean leukocyte

count is 8000/μL blood. Results are expressed as the number of asexual parasites per μL blood. These methods are described in the Field and Laboratory Procedures Manual.

6.2.1.2 Sample Separation and DNA Extraction

All 250μL finger stick blood sample collected will be separated into plasma and cell pellets and frozen and stored separately. Blood from 10 mL venous blood draw will be separated into plasma, peripheral blood mononuclear cells (PBMCs) and red blood cell (RBC) fractions using standard protocols. Fractions from 10 mL venous samples will be aliquoted and frozen as required. Detailed protocol for sample separation will be described in the laboratory SOPs.

Deoxyribonucleic acid extraction will be performed using the QIAmp 96 blood extraction protocol (QIAGEN). This approach enables DNA extraction from 192 samples in 2 to 3 hours and has been used successfully to PCR amplify both human- and malaria parasite-specific amplicons for genotypic and species specific diagnostic assays, respectively.(15),(16) Because DNA extraction methodologies are based upon starting with whole blood, preliminary protease treatment will lyse leukocytes, erythrocytes and *Plasmodium* parasites. Therefore, purified genomic DNA contains a mixture of human and parasite DNA. The protocol for DNA extraction will be in accordance with the procedures set forth in the standard operating procedures (SOPs).

6.2.1.3 Diagnosis of Malaria Infection by Polymerase Chain Reaction/Ligase Detection Reaction

As each individual's genomic DNA preparation contains human and parasite DNA, blood samples can be used directly to perform species-specific PCR/LDR-based diagnosis of *Plasmodium* infection.(16). The protocol for the PCR/LDR-based diagnosis of malaria infection will be in accordance with the procedures set forth in the SOPs. In all samples positive by PCR/LDR we will determine the presence of *P. falciparum* and *P. vivax* gametocytes using a novel reverse transcriptase-PCR assay using dried filter paper blood spots.

6.2.1.4 Host Genetic Typing

All children will be typed for known red blood cell polymorphisms that are known to alter risk for malarial infections. The Duffy antigen typing and Band 3 polymorphism will be done by PCR-based methods described earlier.(17, 18) Glycophorin C (Gerbich phenotypes) and α^+-thalassemia polymorphisms will be assessed using PCR from already published methods with some modifications ((19, 20) and Pascal Michon, unpublished data). In addition, we will genotype children for a series of genetic loci related to host immune response (including but not restricted to *TLRs, DCIRs, NKRs, KIRs, HLA, cytokine, chemokines and Fc receptor polymorphisms*).

At least 50 μL of extracted DNA are needed to conduct both parasite typing and host genetic assays.

6.2.1.5 Parasite Genotyping

All PCR-positive *P. falciparum* and *P. vivax* infections will be genotyped using two capillary electrophoresis based PCR fragments sizing assays developed in the laboratory of Dr. Ingrid Felger at Swiss Tropical Institute in Basel Switzerland (14, 21). Both assays are currently being transferred to the PNGIMR malaria molecular laboratories in Goroka and Yagaum.

6.2.1.6 Immunological evaluations

The in-depth immunological and molecular epidemiology studies for this project will be conducted in collaboration with a number of investigators including: Dr Louis Schofield and Dr James Beeson, Walter and Eliza Hall Institute of Medical Research, Australia, Dr Hernando del Portillo and Dr Pedro Alonso, Centre de Recerca en Salut Internacional de Barcelona, Spain.

Detailed supplementary protocols outlining objectives, methodologies and outcomes for the cellular and humoral immunology components of this study are being prepared and will be submitted to the relevant institutional ethical review boards, in addition to the PNG Medical Research Advisory Committee.

6.2.2 Specimen Collection, Preparation, Handling and Shipping

6.2.2.1 Instructions for Specimen Preparation, Handling, and Storage

6.2.2.1.1. Blood Collection

Baseline Bleed

In order to have sufficient blood for both the measurements detailed in this protocol as well as those in the supplementary immunology studies, a venous blood sample of approximately 10 mL will be collected at baseline. The sample will be collected in heparinized tubes and separated into red cell, white cell and plasma fractions. The fractions will be frozen as required for laboratory analyses.

Drawing venous blood samples from small children poses considerable challenges and needs to be done by specially trained staff. All samples will thus be drawn by PNGIMR staff with the appropriate medical qualifications, registrations and training to draw venous blood samples from small children.

We will use butterfly needles of 23 gauge and 10 mL heparinized vacutainers. The use of 10 mL vacutainers will result in an effective blood draw of 8-10 mL venous blood. Residual blood in the tube attached to the butterfly needle will be used to measure Hb levels and make blood smears and filter paper spots.

The exclusion of severely anemic children (Hb <5 g/dL) assures that the drawing of 10 mL venous blood is of no potential harm to the participants.

Follow-up and Morbidity Surveillance

Finger prick blood samples of approximately 200 -250 µL will be collected every two weeks for the first 12 weeks and 4 weekly thereafter from each child participating in the study. Additional samples will be taken from symptomatic individuals at both active and passive morbidity surveillance visits. Blood samples will be obtained by the finger-stick method. This sample will provide adequate blood for the preparation of thin/thick blood smears to detect the presence of malaria parasites, type of malaria parasites, and level of parasitaemia. At baseline and the final visit, part of the sample will also be used to determine Hb concentration. The samples collected in microtainers will be processed into cell pellet (for DNA preparation, PCR/LDR-based diagnosis of malaria parasites and Duffy genotyping) and plasma (for immunological investigations).

Preparation of Blood Smears

The preparation of thin and thick blood smears will be in accordance with the procedures set forth in the SOPs.

Determining Hemoglobin Concentration

Hemoglobin concentration will be determined by using a HemoCue® device (HemoCue, Ångholm, Sweden) in the field and will be expressed as grams of Hb per deciliter. The instrument measurements shall be made to the nearest tenth. These machines are routinely cleaned and calibrated to ensure accurate measurement.

6.2.2.2 Specimen Shipment

At regular intervals, blood smears, and blood samples will be transported via road and air (Air Nuigini) from the PNGIMR's Maprik field station to the PNGIMR's Madang field office. Delivery and receipt of blood smears, and blood samples will be confirmed by both e-mail and telephone. Details for storing and shipping blood smears and blood samples are outlined in the SOPs.

Upon receipt of these items in Madang, the blood samples will be delivered to the PNGIMR lab manager. Blood smears will be delivered to the PNGIMR's Madang Microscopy Lab for reading to obtain diagnostic confirmation and for archiving.

7 STUDY SCHEDULE

The study schedule is summarized in Table 3. A graphic representation of study activities is given in Appendix I.

Table 3. Summary of scheduled study activities

	Time	Activities
**Community information**	Start 3-4 months prior to Enrolment	- Community and school information meetings- Population Census in village schools
**Screening & Enrollment**	4 weeks prior to Baseline	- Assessment of eligibility<br>- Individual informed consent<br>- G-6-PD testing
**Randomisation**	4 weeks prior to Baseline	- Randomisation of G-6-PD normal children to 2 arms
**Drug Treatment**	4 weeks prior to Baseline	- CQ/PQ/CO and CQ/PL/CO treatment regimes follow identical pattern of administration
**Baseline**	Week 0	- Baseline data collection and 10 mL venous blood sample collection
**Morbidity follow-up (32 weeks)**	Week 2-32	- 2 weekly active morbidity follow-up-Continuous passive case detection-250µl finger pricks at 2, 4, 6, 8, 10, 12, 16, 20, 24, 28 and 32 weeks.

7.1 Community Information

The study team will conduct a number of community information meetings in communities that express interest in participating in the study. In communities that agree to participate a baseline

census will be conducted in order to obtain a list of families who have eligible children (ie, 5 to 10 years of age (±3 month) and are permanently residing in study villages). These families will then be contacted and invited to specific community information meetings (*tok save* in Melanesian Pidgin) where the detailed study plans will be presented to both the parents and/or guardians of eligible children and other interested community members. Families who are interested in enrolling their child in this study will be encouraged to bring their children to the enrollment & pre-screening visit, where a PNGIMR field study team's Nursing Officer will re-assess the child for eligibility. See Section 14.3 for further details of the consent process.

7.2 Enrolment/Prescreening & Randomisation

Upon presentation at the enrollment/screening visit, the PNGIMR study team will confirm the eligibility of participants (see Section 5.2 above for eligibility and exclusion criteria) and administer written informed consent (see Section 14.3 for details of consent procedures).

In the event an eligible child is excluded from the study, the PNGIMR field study team will record the reason for exclusion in the study log book. Children diagnosed with severe malnutrition or severe anemia will be referred to nearest health center or (conditional on parental or guardian consent) admitted to the nearest hospital for treatment. All children will receive (preventative) treatment for anaemia unless medically contraindicated.

All children that have normal G-6-PD activity will be randomized to the different treatment groups

Details of the procedures of enrollment/prescreening visit and randomization are given in Section 6.1.

7.3 Drug treatment & Baseline Visit

After randomization, drug treatment will be started in both arms of the study. A baseline visit including a detailed health assessment and a 10 mL venous blood collection will be conducted at the cessation of the drug treatment regime.

Details of the procedures of drug treatment and the baseline visit are given in Section 6.1.

7.4 Follow-up and Final Visits

7.4.1 Follow-up Visits

Following enrollment the participants will be followed up actively for malaria morbidity (for details see Section 6.1.3.2). A finger prick blood sample will be collected every 2 weeks for the first 12 weeks and 4 weekly thereafter. A schematic of the follow-up design is given in Appendix I.

Children found to have developed severe anemia or severe malnutrition during follow-up will be referred to the health center for appropriate treatment. They may be suspended from participation in the study by the study physician. However, in order to monitor and assist their progress, they will be invited to attend 2 weekly morbidity follow-up clinics held by the study team.

7.5 Early Termination Visit

In case of an early termination, the date and reason for termination will be noted in the participants' database. No specific early termination visit will be done.

7.6 Criteria for Discontinuation or Withdrawal of a Subject

Parents and/or guardians may withdraw their child at any time from the participations in this study.

A participating child will be discontinued from the study if 1 of the following criteria are fulfilled:

- the participant moves a away from the selected study villages for a period longer than 2 months
- the participant develops a chronic illness that impedes continuation in study. Children developing severe anemia (Hb <5 g/dL) or severe malnutrition (WAZ <60%) may be discontinued by the study physician if medically indicated.

Participants who are discontinued because of acute or chronic illness may continue to consult the study team nurses during 2 weekly morbidity visits for health problems, if they wish to do so.

8 ASSESSMENT OF OUTCOME MEASURES

8.1 Specification of the Appropriate Outcome Measures

8.1.1 Primary Outcome Measures

- Time-to-first *P. vivax and P. falciparum* infections by LM and PCR in the 32 week follow-up period. (Aim 1)
- Incidence of first or only *P. vivax and P. falciparum* infection by LM and PCR during the 32 weeks of follow-up. (Aim 1)
- Incidence of clinical *P. falciparum and P. vivax* episodes during the 32 weeks of follow-up. (Aim 1 & 2)
- Incidence of first or only clinical *P. falciparum and P. vivax* episode during the 32 weeks of follow-up. (Aim 1 & 2)

Clinical *P. falciparum* and *P. vivax* malaria is defined as an axillary temperature ≥37.5º(or a history of fever lasting more than 2 days in duration) together with concurrent *P. falciparum* and/or *P. vivax* parasitaemia.

8.1.2 Secondary Outcome Measures

- Incidence of clinical *P. malaria*e and *P. ovale* episodes during the 32 weeks of follow-up. (Aim 1)
- Time-to-first *P. malariae and P. ovale* infections by LM and PCR in the 32 week follow-up period. (Aim 1)
- Incidence of first or only *P. malariae* and *P. ovale* infection by LM and PCR during the 32 weeks of follow-up. (Aim 1)
- Incidence of first or only clinical *P. malaria*e and *P. ovale* episode (Aim 1)

Clinical episodes of *P. malaria*e and *P. ovale* malaria are defined as an axillary temperature ≥37.5ºC (or a history of fever lasting more than 2 days in duration) together with concurrent *P. malaria*e or *P. ovale* parasitaemia.

Outcome measures for immunological studies will be described in separate immunology sub-protocols.

9 SAFETY ASSESSMENT AND REPORTING

This study will treat all children with common anti-malarials with well known safety profiles. Although generally well tolerated in non-G-6-PD deficient children, treatment with primaquine may be associated with mild side effects. The study will therefore actively monitor children for side effects during treatment

As described previously, the PNGIMR has conducted an earlier cohort study in the same population that included careful monitoring of adverse events. Based on our experience from that study, while SAEs occurred regularly none of them were study-related adverse experiences, as defined below..

9.1 Definition of Adverse Event (AE)

Adverse events that may occur from study related activities, such as pre-treatment with chloroquine, artesunate and primaquine, the drawing of blood (an overly deep finger-stick or infection or celluitis at the site of the finger-stick of venipuncture). Only study related AEs will be reported in an annual report to the IRB.

9.2 Definition of Serious Adverse Event

A serious adverse event (SAE) includes any untoward medical occurrence that:

- Results in death during the study period;
- Is life threatening (defined as a subject at immediate risk of death at the time of the event);
- Requires in-patient hospitalization or prolongation of existing hospitalization;
- Results in persistent or significant disability or incapacity or required intervention to prevent permanent impairment or damage;
- Any other important medical event that may not result in death, be life threatening, or require hospitalization may be considered an SAE when, based upon appropriate medical judgment, the event may jeopardize the subject and may require medical or surgical intervention to prevent 1 of the outcomes listed above.

9.3 Reporting Procedures

All SAEs will be reported to the PNG IMR IRB within 3 working days. Adverse events will be reported annually in summary fashion.

9.3.1 Serious Adverse Event Detection and Reporting

Severe adverse events may be detected either at the 2-week regular health check or at any time during passive morbidity surveillance at Albinama Health Center or surrounding Aid Posts. Staff will be trained to recognize SAEs and will be instructed to report them immediately to the study coordinator or the health center based IMR nurse. In addition, parents and/or guardians of participants will be encouraged to report any serious medical problems of their child to the study team as quickly as possible.

For all events meeting the above described definition of Serious Adverse Events, the completion of the PNGIMR Serious Adverse Event report form is required.

The SAE Form will be reviewed by the study physician, who may request additional information or carry out further investigations.

The study clinician will complete an SAE Report Form within the following timelines:

All deaths and life-threatening events, whether related or unrelated, will be recorded on the SAE Report Form and sent by fax within 24 hours of site awareness of the death or life-threatening event to the study clinician and principal investigators.

Serious adverse events, other than death and life-threatening events, and regardless of the relationship, will be reported via fax by the site within 72 hours of site awareness of the event.

9.3.2 Type and Duration of the Follow-up of Subjects after Adverse Events

All SAEs/AEs will be followed until satisfactory resolution or until the Study Clinician deems the event to be chronic or the patient to be stable.

9.4 Halting Rules

The sponsor and/or IRB can however stop the study at any time, if there are overriding concerns for the safety of study participants.

10 CLINICAL MONITORING STRUCTURE

As a self-sponsored study, no external monitoring of study activities will be preformed. In order to assure the safety of participants and integrity of study databases, the study will however follow Good Clinical Practice and quality assurance guidelines as outlines the IMR GCP – guidelines.

The study investigators are committed to providing access to the standard level of care (ie, therapy with approved agents according to the guidelines of the PNG Ministry of Health and the World Health Organization [WHO]) for participants. Children with illnesses that cannot be treated by field clinics will be referred the Albinama Health Center or Maprik Hospital where they will be treated according to PNG standard treatment guidelines.

11 STATISTICAL CONSIDERATIONS

The analyses of this study will follow standard approaches for cohort studies. Time-to-event outcomes will be analysed using survival analysis, incidence rates by Poisson regression.

11.1 Study Outcome Measures

11.1.1 Definitions of Malaria Infections

Symptomatic *Plasmodium* Infection

For diagnostic purposes, a symptomatic *Plasmodium* infection is defined as any type of *Plasmodium* parasitemia observed by blood smear and/or a positive rapid diagnostic test, plus an axillary temperature ≥ 37.5°C or a history of febrile illness in the last 48 hours.

However, for analytical purposes we will use 2 different definitions for symptomatic *Plasmodium* infections: a) any type of *Plasmodium* parasitemia observed by blood smear and/or PCR/LDR diagnostic assay, plus an axillary temperature ≥37.5°C or a history of febrile illness in last 48 hours b) a parasite density of >5000/µL for *P. falciparum* and/or >500/µL for non-*falciparum*, plus an axillary temperature ≥ 37.5°C or a history of febrile illness in the last 48 hours.

Asymptomatic Blood-stage *Plasmodium* Infection

An asymptomatic blood-stage *Plasmodium* infection is defined as any type of *Plasmodium* parasitemia observed by blood smear or PCR-LDR confirmation with an axillary body temperature less than 37.5°C and no history of febrile illness in last 48 hours.

11.1.2 Primary Outcome Measures

11.1.2.1 Incidence of clinical *P. falciparum* or *P. vivax* episodes during the 32 weeks of follow-up

The comparison of the incidence of clinical *P. falciparum* and *P. vivax* episodes between the 2 study arms is a primary objective of the study and will be evaluated by assessing differences in both the incidence of *P. falciparum P. vivax* episodes over 32 weeks of follow-up and the incidence of first or only *P. falciparum* / *P. vivax* episodes

Children will be considered ‘at risk’ until they reached the endpoint of interest, withdrew, were lost to follow-up or completed the study. For clinical and molecular endpoints children were censored on the last day before the first of two consecutively missed active follow-up visits

11.1.2.2 Incidence of first or only *P. falciparum or P. vivax* infection

The analyses of difference in incidence of first or only *P. falciparum* / *P. vivax* infections will follow that approach for analyses of first or only *P. falciparum* / *P. vivax* episodes, with the difference that children will be considered at risk only until they experience the first *P. falciparum* / *P. vivax* infection.

11.1.2.3 Time to first *P. falciparum* / *P. vivax* infection

For the analyses of time to first *P. falciparum* / *P. vivax* infection children will be at risk until they either experience a *P. falciparum* / *P. vivax* infection or they are treated with antimalarial drug for another malarial infection. At the time of the first antimalarial treatment, the child will be censored from analysis.

11.1.3 Secondary Outcome Measures

Analyses of secondary outcomes (i.e. incidence of *P. malariae* or *P. ovale* clinical episodes or time to first *P. malariae* or *P. ovale* infections) will follow the same approaches as those employed for analyses of *P. falciparum* and *P. vivax* infections and morbidity, as described above.

11.2 Sample Size Considerations

11.2.1 Primary Outcome Measures

No estimates for time to first *P. vivax* infection are available for the study community however, in the above-mentioned treatment time-to-reinfection study in children 6-12 years of age in neighbouring Madang province the median time re-infection was 54 and 118 days for PCR and light microscopically detected *P. vivax* infections, respectively. Assuming a 50% decrease in time to first new *P. vivax* infections following LLIN distribution then a sample size of 200 children per arm be sufficient to find a reduction of 30% (hazard ratio 0.70) and 34% (hazard ratio 0.66) of PCR and LM-detectable *P. vivax* infections in the CQ/PQ/CO compared to the CQ/PL/CO group with a power of 90%. This sample size was increase by 25% to account for loss of follow-up and by 5% for children that receive less than 14 days of directly observed primaquine treatment/

In a recently completed cohort study in this region, the incidence of clinical *P. falciparum* malaria in children aged 1-3 years was estimated at 2.6 episodes per child per year (any density). Although no estimates for the incidence of clinical *P. falciparum* malaria in children aged 5-10 years in this region, in a recent treatment time-to-reinfection study in children 6-12 years of age in neighbouring Madang province, the incidence was estimated at 1.7 episodes per child per year (any density). The recent distribution of long-lasting insecticide treated bednet (LLIN) will have reduced this incidence by 60%.

Thus, assuming an incidence of 0.5 episode of *P. falciparum* per child per year (any density in this cohort of similarly aged children, a sample size of 500 children will thus be sufficient to detect 20% decrease in incidence between the two treatment arms with a power of 90%.

11.2.2 Secondary Outcome Measures

The analyses for secondary *P. falciparum* infection outcomes and *P. vivax* malaria outcomes are comparable to those described for *P. vivax* infection and *P. falciparum* episodes above. The relative scarcity of *P. malariae* and *P. ovale* infections and morbidity in the proposed study age group means that the study will lack power to find any but large differences between the two arms (hazard ratio < 0.5).

11.3 Analysis Plan

11.3.1 Primary Outcome Measures

11.3.1.1 Incidence of clinical *P. falciparum* episodes during follow-up

This is a primary objective of the study and will be evaluated by assessing differences in the risk of *P. falciparum* clinical malaria with in both treatment arms.

The analysis strategy for these outcomes is centered on different variations of Poisson regression. The outcome variables are total number of *P. falciparum* episodes (over 32 weeks of follow-up) and incidence of 1st or only episode. Exposure will be defined as the total risk time per child as outlined above. Analyses will be adjusted for possible covariates including infection status at baseline, sex, age, host genetic traits, and nutritional state of child.

The analyses will be done both for episodes defined as axillary temperature ≥37.5ºC plus any parasitemia or axillary temperature ≥37.5ºC plus *P. falciparum* >5000/µL.

11.3.1.2 Incidence of first or only *P. vivax* infection

The analyses approach to this objective is identical to the one for incidence of 1st or only *P. falciparum* episode. Analyses will include incidence of 1st or only episode detectable by PCR or light microscopy as well as infections exceeding different density cut-offs.

11.3.1.3 Time-to-first *P. vivax* infection

Time to first infection will be analyzed using standard survival analysis, including log rank test for the comparison of survival curves and cox regression for modeling survival times. Time at risk will be defined as as outlined above. Cox regression analyses will be adjusted for possible covariates include infection status at baseline (i.e. before start of treatment), sex, age, host genetic traits, and nutritional state of child. Analysis will include analyses of time to 1st *P. vivax* only infection detectable by PCR or light microscopy as well as infections exceeding different density cut-offs.

11.3.2 Secundary Outcome Measures

Analyses for secondary *P. falciparum*, *P. vivax, P. malariae* and *P. ovale* outcomes follow identical approaches as those outlined above for *P. vivax* infections and *P. falciparum* morbidity.

Statistical analyses will be done using STATA, SAS and BUGS statistical software.

12 ACCESS TO SOURCE DATA/DOCUMENTS

Each participating site will maintain appropriate medical and research records for this study, in compliance with Section 4.9 of ICH E6 GCP and institutional requirements for the protection of confidentiality of subjects. Each site will permit authorized representatives of the IMR IRB to examine (and when required by applicable law, to copy) clinical records for the purposes of quality assurance reviews, audits and evaluation of the study safety and progress.

Source data are all information, original records of clinical findings, observations, or other activities in a study necessary for the reconstruction and evaluation of the study.

13 QUALITY CONTROL AND QUALITY ASSURANCE

This study will be conducted according with the procedures outlined in this protocol.

In order to assure both compliance with the protocol and assure the quality of the data collected local quality control procedures will be put in place. The different tasks to be performed in field and lab will defined in specific standard operating procedures (SOPs). Adherence to both protocol and SOPs will monitored via regular spot checks on collection of primary field data, checks of all forms by study coordinator or designee upon receipt from field teams, double reading of all blood slide collected and regular data audits following 2nd entry of all databases. In addition, the PI will regularly perform internal audits to assess completeness and accuracy of study files, CRFs, source documents, and ethical standards of study operations.

All specimens collected as well as their transfer between different sites will be recorded in specimen log book. Similarly, transfer of CRFs between sites will be recorded in a CRF log. The site laboratory investigators will regularly check lab books and documents to assure compliance with laboratory SOPs.

The detailed quality control and quality assurance procedures will be set out in specific standard operating procedures (SOPs) for quality management.

14 ETHICS/PROTECTION OF HUMAN SUBJECTS

14.1 Declaration of Helsinki

The investigator will ensure that this study is conducted in full conformity with the current revision of the Declaration of Helsinki, or with the International Conference for Harmonisation Good Clinical Practice (ICH-GCP) regulations and guidelines, whichever affords the greater protection to the subject.

14.2 Institutional Review Board

The project will be reviewed by the following Institutional Review Boards:

PNG Institute of Medical Research Institutional Review Board (IMR IRB)

PNG Medical Research Advisory Committee (MRAC).

14.3 Informed Consent Process

Following standard practice for PNGIMR field studies, the informed consent process starts well prior to enrollment of participants and involves both community and individual consent.

Upon funding approval, the study was discussed with senior community members employed by the PNGIMR as community relations officers to assess both feasibility and community acceptability of different study design features and field procedures. Comments and suggestions by the community relations' officers were integrated into the final study protocol.

As a first step in the informed consent process, the PI and community liaison staff will hold meetings with community leaders/elders and elementary school teachers in all potential study villages. At these meetings study objectives and procedures as well as risk and possible benefits for the individual participants and the community will be discussed in detail. The study brochure (see Attachment III) will be presented to the community leaders, but no written consent sought.

Following consent by community leaders and school officials to include their village into the study, the study team will conduct a school census and identify eligible study participants. Parents and/or guardians with eligible children will subsequently be invited to study information meetings (*tok save* in Melanesian Pidgin) held in each participating elementary school. At these meetings, the study team will describe the purpose and significance of the study, the procedures to be followed, the risks and benefits of participation, and state that participation in the study is voluntary and that

declining to participate will not reduce the level of, or access to, health care for the child or family. At the same time the parents and/or guardians will presented with the informed consent documents.

Many people in Papua New Guinea, especially those with limited literacy skills are reluctant to sign lengthy documents. In order to accommodate this, the study will use 2 distinct informed consent documents: a detailed study brochure and a shorter informed consent signature form. The study brochure contains all the necessary information for informed consent in easily understandable language. The informed consent signature form contains shortened summary of the study information and a signature page. Both forms are included in Appendices III and IV. All informed consent documents will be approved by the PNG MRAC and overseas IRBs.

The *tok saves* will be held in Melanesian Pidgin and parents and/or guardians of the prospective participant will receive consent documents in the language in which the parents and/or guardians are most conversant (Melanesian Pidgin or English).

At the end of the *tok saves,* a date and location for the enrollment/baseline study visits are set and interested parents and/or guardians are asked to bring their child(ren) together with the informed consent signature form. Extra copies of informed consent documents will be available at the enrollment for parents and/or guardians that have misplaced and forgotten their documents. Prior to signing the informed consent form, the parents and/or guardians will be queried for any uncertainties regarding the study and their child's participations and the study team will answer all questions the parents and/or guardians may have.

The signature form may be signed by 1 parent or guardian (normally the father) prior to the enrollment/baseline visit if the parents will not accompany the child to the enrollment/baseline visit. If both parents and/or guardians are illiterate, the parent and/or guardian accompanying the child to the enrollment visit will be asked to mark the signature form and an independent witness will be asked to document the informed consent process by signing the form. A copy of the informed consent signature form will be given to the subjects for their records.

Only children whose parents and/or guardians have signed the informed consent signature form will be enrolled in the study.

In the event the parents and/or guardians of the participants decline to participate, the PNGIMR field study team will record the reason in the study log book.

The parents and/or guardians may withdraw consent for their child's participation at any time throughout the course of the study.

At any time of the study, the study team will be available to answer questions of parents and/or guardians of participants regarding any aspect of the present study.

Samples from this study will be used for in-depth immunology studies outlined in a separate immunology sub-protocol. Consent for use of samples for these studies will be integrated into the above consent process

14.4 Subject Confidentiality

The confidentiality of participant information will be maintained at all times. Participant information will be identified by the study identification number and/or blood sampling code and serial number as appropriate. Blood samples will be tagged using the appropriate unique blood sampling codes and serial numbers.

In order to protect confidentiality, any data or sample that leaves PNG will be fully anonymized. All analysis datasets will not contain any personal identifier or other variable that allow identification of individual participants.

Equally, no personal information or data that allow identification of individual participants will be included in any reports or scientific publications.

The study monitor or other authorized representatives of the sponsor may inspect all documents and records required to be maintained by the Investigator, including but not limited to, medical records (office, clinic or hospital) and pharmacy records for the subjects in this study. The clinical study site will permit access to such records.

14.5 Future Use of Stored Specimens

Specimens collected in this study will be used for in-depth immunological and parasitological studies to be outlined in separate sub-protocols. In particular they will be used for determination of naturally acquired immune responses to *P. falciparum* and *P. vivax* vaccine candidates and selected non-vaccine antigens and their association with subsequent risk of *P. falciparum* and *P. vivax* infection and disease in children 5-10 years of age (See Section 3, Aim 2 above).

All residual specimens will be maintained at the PNGIMR. Should any residual specimen remain from laboratory analyses done at collaborating overseas laboratories, as mandated by PNG law, they remain the exclusive property of the PNGIMR (as the custodian of the State of PNG), even if not immediately repatriated.

As part of the informed consent process permission, parents and/or guardians of participants will be asked if they grant permission to use residual specimens of their child for future malaria research studies. In line with PNG guidelines, this is sufficient to allow the use of sample for future malaria

studies, if the studies and the use of samples has been approval by the IMR IRB and the PNG Medical Research Advisory Council.

15 DATA HANDLING AND RECORD KEEPING

15.1 Data Management Responsibilities

The primary data will be collected by field and laboratory staff at the different sites.

Upon receipt of CRF and source documents from the field team, the study coordinator (or his/her designee) will crosscheck all CRFs for completeness, validity, and legibility. Upon crosscheck, all CRFs and laboratory worksheets will be photocopied. Originals will be maintained in the PNGIMR's field research office in Maprik until completion of the field work, copies will be sent to designated PNGIMR data entry unit in Madang.

All data from CRFs and laboratory worksheets in PNG will be entered into a custom made study database using DMSys (SigmaSoft) clinical trial data management software. This software meets all requirements of ICH GCP E6 and regulatory agency for data management in clinical research studies.

All data will be double entered with first entry will be done immediately after receipt of CRF and worksheet copies from the PNGIMR field station in Maprik. Second entry will be done by a different data entry clerk once a batch of first entry is completed. All data entry will be done by specifically trained data entry personnel. Queries for discrepancies, missing values, out-of-range and internal consistency will be done by the data base manager on a weekly basis.

Access to the study database will be limited to the data entry personnel, data managers of the PNGIMR and collaborating institutions.

Detailed data management procedures will be described in the Data Entry and Data Management standard operating procedures. Regular data audits will be conducted to assure quality of data and adherence to SOPs.

15.2 Data Capture Methods

Source documents in this study will consist of the following:

- The Field study logbook relating to study enrollment and continued participation in cohort;
- Case report forms (CRFs) completed by the field study team during study visits (whether scheduled or non-scheduled) with each participant;
- Health Center logbooks;

- Health Center Surveillance form abstracts documenting illness and treatment histories for participants who seek care from participating health centers;
- Children's personal health books
- Microscopy laboratory worksheets documenting the evaluation of blood smears;
- Laboratory notebooks documenting DNA extraction and laboratory assays

The study coordinator and site investigator (for laboratory data) will check all CRFs prior to passing the forms on to data entry. If corrections need to be made, the incorrect entry will be crossed out with a single line and the correct information will be printed adjacent to it. The correction must be initialed and dated by designated, qualified study staff. Any requested information that is not obtained as specified in the protocol should have an explanation noted on the CRF as to why the required information was not obtained.

15.3 Timing/Reports

Data entry and review will be ongoing activities during the entire field study period. Data will be fully cleaned and locked prior to any data analysis. No intermediate data analysis will be conducted.

15.4 Study Records Retention

Hard copies of study records will be maintained for at least 5 years following completion of the study.

Electronic copies of all frozen databases will be kept indefinitely at the PNGIMR in Goroka.

15.5 Protocol Deviations

The protocol will be adhered to for the entire duration of the study. In particular, no deviations from enrollment and exclusion criteria are permitted.

Adherence to protocol will be monitored by the study coordinator. In addition, protocol violations (eg, exclusion criteria) will be assessed regularly as part of the QC/QA procedures.

Should protocol deviations occur, the study supervisor will inform the principal investigator of the time and nature of the deviations. The PI will then inform the IRBs and if required, amendments to the protocol will be sought.

A protocol deviation is any noncompliance with the clinical trial protocol, GCP, or Manual of Procedures requirements. The noncompliance may either be on the part of the subject, the investigator(s), or the study staff. As a result of deviations, corrective actions should be developed by the site and implemented properly.

It is the responsibility of the site to use continuous vigilance to identify and report deviations within 5 working days to the study coordinators, who will maintain a log of protocol deviation log. All deviations from the protocol must be addressed in the subject's CRF. The study coordinator will take the necessary steps to prevent re-occurrence of similar deviations.

.

16 PUBLICATION POLICY

All results obtained from this study will be submitted to peer-reviewed biomedical journals for publication.

Summary results will be given back to parents and community on a yearly basis.

17 LITERATURE REFERENCES

1. Mendis K, Sina BJ, Mechesini P, Carter R. The neglected burden of *Plasmodium vivax* malaria. Am J Trop Med Hyg. 2001;64:97-106.

2. Ozen M, Gungor S, Atambay M, Daldal N. Cerebral malaria owing to Plasmodium vivax: case report. Annals of tropical paediatrics. 2006 Jun;26(2):141-4.

3. Kochar DK, Saxena V, Singh N, Kochar SK, Kumar SV, Das A. Plasmodium vivax malaria. Emerging infectious diseases. 2005 Jan;11(1):132-4.

4. Genton B, D'Acremont V, Rare L, Baea K, Reeder JC, Alpers MP, et al. Plasmodium vivax and mixed infections are associated with severe malaria in children: a prospective cohort study from Papua New Guinea. PLoS Med. 2008 Jun 17;5(6):e127.

5. Tjitra E, Anstey NM, Sugiarto P, Warikar N, Kenangalem E, Karyana M, et al. Multidrug-resistant Plasmodium vivax associated with severe and fatal malaria: a prospective study in Papua, Indonesia. PLoS Med. 2008 Jun 17;5(6):e128.

6. Barcus MJ, Basri H, Picarima H, Manyakori C, Sekartuti, Elyazar I, et al. Demographic risk factors for severe and fatal vivax and falciparum malaria among hospital admissions in northeastern Indonesian Papua. Am J Trop Med Hyg. 2007 Nov;77(5):984-91.

7. Chareonviriyaphap T, Bangs MJ, Ratanatham S. Status of malaria in Thailand. Southeast Asian J Trop Med Public Health. 2000;31:225-37.

8. Luxemburger C, Thwai KL, White NJ, Webster HK, Kyle DE, Maelankirri L, et al. The epidemiology of malaria in a Karen population on the western border of Thailand. Trans R Soc Trop Med Hyg. 1996 Mar-Apr;90(2):105-11.

9. Chaves SS, Rodrigues LC. An initial examination of the epidemiology of malaria in the state of Roraima, in the Brazilian Amazon basin. Rev Inst Med Trop Sao Paulo. 2000;42:269-75.

10. Michon P, Cole-Tobian JL, Dabod E, Schoepflin S, Igu J, Susapu M, et al. The risk of malarial infections and disease in Papua New Guinean children. Am J Trop Med Hyg. 2007 Jun;76(6):997-1008.

11. D'Ombrain MC, Robinson LJ, Stanisic DI, Taraika J, Bernard N, Michon P, et al. Association of early interferon-gamma production with immunity to clinical malaria: a longitudinal study among Papua New Guinean children. Clin Infect Dis. 2008 Dec 1;47(11):1380-7.

12. Stanisic DI, Richards JS, McCallum FJ, Michon P, King CL, Schoepflin S, et al. Immunoglobulin G subclass-specific responses against Plasmodium falciparum merozoite

antigens are associated with control of parasitemia and protection from symptomatic illness. Infect Immun. 2009 Mar;77(3):1165-74.

13. Kaneko A, Taleo G, Kalkoa M, Yamar S, Kobayakawa T, Bjorkman A. Malaria eradication on islands. Lancet. 2000 11/4/2000;356(9241):1560-4.

14. Falk N, Maire N, Sama W, Owusu-Agyei S, Smith T, Beck HP, et al. Comparison of PCR-RFLP and Genescan-based genotyping for analyzing infection dynamics of Plasmodium falciparum. Am J Trop Med Hyg. 2006 Jun;74(6):944-50.

15. Zimmerman PA, Woolley I, Masinde GL, Miller SM, McNamara DT, Hazlett F, et al. Emergence of FY*Anull in a Plasmodium vivax-endemic region of Papua New Guinea. Proceedings of the National Academy of Sciences USA. 1999;96(24):13973-77.

16. McNamara DT, Thomson JM, Kasehagen LJ, Zimmerman PA. Development of a Multiplex PCR-Ligase Detection Reaction Assay for Diagnosis of Infection by the Four Human Malaria Parasite Species. JCM. 2003;submitted.

17. Jarolim P, Palek J, Amato D, Hassan K, Sapak P, Nurse GT, et al. Deletion in erythrocyte band 3 gene in malaria-resistant Southeast Asian ovalocytosis. ProcNatlAcadSciUSA. 1991 12/15/1991;88(24):11022-6.

18. Zimmerman PA, Woolley I, Masinde GL, Miller SM, McNamara DT, Hazlett F, et al. Emergence of FY*A(null) in a Plasmodium vivax-endemic region of Papua New Guinea. Proc Natl Acad Sci U S A. 1999 11/23/1999;96(24):13973-7.

19. Patel SS, Mehlotra RK, Kastens W, Mgone CS, Kazura JW, Zimmerman PA. The association of the glycophorin C exon 3 deletion with ovalocytosis and malaria susceptibility in the Wosera, Papua New Guinea. Blood. 2001 12/1/2001;98(12):3489-91.

20. Chong SS, Boehm CD, Cutting GR, Higgs DR. Simplified multiplex-PCR diagnosis of common southeast asian deletional determinants of alpha-thalassemia. Clin Chem. 2000 Oct;46(10):1692-5.

21. Koepfli C, Mueller I, Marfurt J, Goroti M, Sie A, Oa O, et al. Evaluation of Plasmodium vivax Genotyping Markers for Molecular Monitoring in Clinical Trials. Journal of Infectious Diseases. 2009;199:1074-80.

18 SUPPLEMENTS/APPENDICES
