# Supplementary material for: Strategies for Understanding and Reducing the Plasmodium vivax and Plasmodium ovale Hypnozoite Reservoir in Papua New Guinean Children: A Randomised Placebo-Controlled Trial and Mathematical Model
Source: PLoS Med. 2015 Oct 27;12(10):e1001891. doi: 10.1371/journal.pmed.1001891 (PMC4624431; doi:10.1371/journal.pmed.1001891)
Supplement: S3 Text — (PDF) [file pmed.1001891.s006.pdf]

Detailed description of *P. vivax* transmission model

Malaria control strategies based on the treatment of at-risk populations will not only protect treated individuals, but also the wider community due to the population-level reduction in malaria transmission. Accounting for this community-level protection requires an understanding of malaria transmission dynamics. The dynamics of *P. falciparum* transmission have been well-described using classical Ross-MacDonald compartmental deterministic models [1,2]. Here, this approach is extended to incorporate relapse infections characteristic of *P. vivax*.

S1 Fig shows a schematic representation of a compartmental model of *P. vivax* transmission and the associated system of differential equations. Humans can be in one of four states: S_0, fully susceptible; I_0, infected with blood-stage parasites; I_L, infected with blood-stage and liver-stage parasites; and S_L, infected with liver-stage parasites. Mosquitoes can be in one of two states: S_M, not infectious (i.e. sporozoite negative); and I_M, infectious (i.e. sporozoite positive). Full definitions of all parameters are given in Supplementary Table S1. Individuals will be exposed to new infectious bites at a rate $\lambda = mabI_M$which will cause new blood-stage infections (if not already infected) and new liver-stage infections (if not already carrying hypnozoites). Individuals carrying hypnozoites can relapse at rate f and naturally clear the hypnozoite reservoir at rate γ (e.g. due to the death of hepatocytes). The expected number of relapses per primary infection can be calculated as$h = f/\gamma$.

Although the model described in S1 Fig captures the key drivers of the dynamics of *P. vivax* transmission, it is a simplified representation subject to a number of limiting assumptions. Most notably, there is no heterogeneity or seasonality in transmission, no treatment of symptomatic episodes, no super-infection, no age structure and no acquisition of natural immunity. Incorporation of these factors would change the quantitative predictions of the model, but most likely not its qualitative behaviour. The model can be simplified by removing the compartments for infection with liver-stage hypnozoites (S_L and I_L) and hence capture the dynamics of *P. falciparum* transmission [2]. In particular, we assume the parameters for the *P. falciparum* and *P. vivax* models are the same, except for the relapse parameters, which apply only to the *P. vivax* model, and the number of mosquitoes per human m. Although quantities such as the duration of blood-stage infection may differ between *P. vivax* and *P. falciparum* for the purposes of this qualitative analysis they are assumed equal here. In addition *P. vivax* or *P. falciparum* gametocytes are not explicitly

accounted for. It is assumed that individuals infected with either asexual *P. vivax* or *P. falciparum* parasites are capable of transmitting to mosquitoes. Furthermore, we do not account for the effects of primaquine on clearance of gametocytes

Interventions

A potential strategy for malaria control is to administer combinations of anti-malarial drugs to entire populations. Schizonticidal drugs such as chloroquine (CQ), artemether-lumefantrine or dihydroartemisinin-piperaquine (DHA-PIP) can be used to clear *P. falciparum* or *P. vivax* blood-stage infections. *P. vivax* liver-stage infection with hypnozoites can only be cleared with an 8-aminoquinolines such as primaquine or tafenoquine (TQ). In addition to clearing parasites, drugs can provide a period of prophylactic protection where new infections are prevented. Primaquine and tafenoquine cannot be given to G6PD-deficient people [3]. Both are pro-drugs that need to be metabolised by the cytochrome P450 (CYP) 2D6 enzyme to be effective [4,5] and people with low 2D6 activity will fail primaquine treatment. In PNG ~5% of people are expected to be moderately to highly deficient (<40% activity [6]) and <5% low CYP 2D6 metabolisers [7].

Two delivery strategies are considered:

- **Mass drug administration (MDA):** individuals in a population are treated regardless of infection status. The proportion of individuals treated is referred to as the coverage.
- **Mass screen and treat (MSAT):** individuals in a population are tested for the presence of blood-stage parasites with an appropriate diagnostic and treated only if they test positive. The proportion of individuals tested is referred to as the coverage.

For both of these strategies we assume two treatment rounds (each covering 80% of the population) spaced 6 months apart. Importantly, we assume no correlation of coverage between treatment rounds, i.e. the probability of being treated in round 2 is independent of treatment status in round 1. If targeted for treatment with blood-stage drugs (DHA-PIP or CQ), it is assumed that individuals in the I_0 compartment move to the S_0 compartment, and individuals in the I_L compartment move to the S_L compartment. These individuals are thenprotected from reinfection for a period of time due to prophylaxis. Treatment with a combination of hypnozoiticidal drugs (PQ or TQ) and blood-stage drugs is assumed to move all individuals to the S_0 compartment and provide a period of prophylactic protection.

S2 Fig shows the prevalence of blood-stage *P. vivax* and *P. falciparum* parasites following two rounds of either MDA or MSAT predicted by the deterministic transmission model.

Stochastic implementation

The model described by the equations in S1 Fig can be implemented in a stochastic framework to capture the natural variation that may occur in finite populations due to the chance nature of infectious events., and the possibility of elimination. At time t the number of humans and mosquitoes in each compartment is $\left\{S_0^t, I_0^t, I_L^t, S_L^t, S_M^t, I_M^t\right\}$. During time step Δt stochastic transitions of individuals between compartments will result in an updated state of the system at time $t + \Delta t$: $\left\{S_0^{t+\Delta t}, I_0^{t+\Delta t}, I_L^{t+\Delta t}, S_L^{t+\Delta t}, S_M^{t+\Delta t}, I_M^{t+\Delta t}\right\}$.

Consider individuals moving in and out of the I_0 compartment. Individuals recover from I_0 to S_0 at rate r, and move from I_0 to I_L through infection at rate λ. The total number of individuals leaving compartment I_0 at time t will be given by a Binomial distribution $I_0^- = B\left(I_0^t, (\lambda + \mathrm{r})\Delta t\right)$. The I_0^- individuals moving out of I_0 will move to S_0 or I_L according to competing hazards determined by a Binomial distribution as follows: $\left\{I_0 \rightarrow S_0, I_0 \rightarrow I_L\right\} = B\left(I_0^-, \left\{\frac{r}{\lambda+r}, \frac{\lambda}{\lambda+r}\right\}\right)$. The number of individuals in compartment I_0 at time $t + \Delta t$ is then given by $I_0^{t+\Delta t} = I_0^t - I_0^- + \left(I_L \rightarrow I_0\right)$. The stochastic transitions between other compartments are similarly defined.

References

1. Smith D, McKenzie F. Statics and dynamics of malaria infection in Anopheles mosquitoes. Malar J. 2004;3-13.

2. Smith DL, Battle KE, Hay SI, Barker CM, Scott TW, McKenzie FE. Ross, Macdonald, and a Theory for the Dynamics and Control of Mosquito-Transmitted Pathogens. PLoS Pathog. 2012;8: e1002588. doi:10.1371/journal.ppat.1002588

3. Howes RE, Battle KE, Satyagraha AW, Baird JK, Hay SI. G6PD deficiency: global distribution, genetic variants and primaquine therapy. Advances in Parasitology. 2013 Elsevier Ltd. pp. 133–201. doi:10.1016/B978-0-12-407826-0.00004-7

4. Bennett J, Pybus, B, Yadava A, Tosh D, Sousa J, McCarthy W, et al. Primaquine Failure and Cytochrome P-450 2D6 in Plasmodium vivax Malaria. N Engl J Med. 2013;369: 1381–2. doi:10.1056/NEJMc1309899

5. Marcsisin SR, Sousa JC, Reichard GA, Caridha D, Zeng Q, Roncal N, et al. Tafenoquine and NPC-1161B require CYP 2D metabolism for anti-malarial activity : implications for the 8-aminoquinoline class of anti-malarial compounds. Malar J. 2014;13: 1–9.

6. Betuela I, Rosanas-Urgell A, Kiniboro B, Stanisic DI, Samol L, de Lazzari E, et al. Relapses contribute significantly to the risk of Plasmodium vivax infection and disease in Papua New Guinean children 1-5 years of age. J Infect Dis. 2012;206: 1771–80. doi:10.1093/infdis/jis580

7. Ahsen N Von, Tzvetkov M, Karunajeewa HA, Gomorrai S, Ura A. CYP2D6 and CYP2C19 in Papua New Guinea : High frequency of previously uncharacterized CYP2D6 alleles and heterozygote excess. Int J Mol Epidemiol Genet. 2010;1: 310–319.
